# Facile Synthesis of a Stable Side‐on Phosphinyne Complex by Redox Driven Intramolecular Cyclisation

**DOI:** 10.1002/chem.201905750

**Published:** 2020-08-07

**Authors:** Helge Lange, Henning Schröder, Elisabeth Oberem, Alexander Villinger, Jabor Rabeah, Ralf Ludwig, Klaus Neymeyr, Wolfram W. Seidel

**Affiliations:** ^1^ Institut für Chemie Universität Rostock Albert-Einstein-Str. 3a 18059 Rostock Germany; ^2^ Institut für Mathematik Universität Rostock Ulmenstraße 69 18057 Rostock Germany; ^3^ Leibniz-Institut für Katalyse e.V. Albert-Einstein-Straße 29a 18059 Rostock Germany

**Keywords:** alkyne complex, cyclisation mechanism, frustrated Lewis pair, non-innocent ligand, phosphinyne complex

## Abstract

Alkyne complexes with vicinal substitution by a Lewis acid and a Lewis base at the coordinated alkyne are prospective frustrated Lewis pairs exhibiting a particular mutual distance and, hence, a specific activation potential. In this contribution, investigations on the generation of a W^II^ alkyne complex bearing a phosphine as Lewis base and a carbenium group as Lewis acid are presented. Independently on potential substrates added, an intramolecular cyclisation product was always isolated. A subsequent deprotonation step led to an unprecedented side‐on λ^5^‐phosphinyne complex, which is interpreted as highly zwitterionic according to visible absorption spectroscopy supported by TD‐DFT. Low‐temperature ^31^P NMR and EPR spectroscopic measurements combined with time‐dependent IR‐spectroscopic monitoring provided insights in the mechanism of the cyclisation reaction. Decomposition of the multicomponent IR spectra by multivariate curve resolution and a kinetic hard‐modelling approach allowed the derivation of kinetic parameters. Assignment of the individual IR spectra to potential intermediates was provided by DFT calculations.

## Graphical abstract

**A sought‐after combination** of a phosphine as Lewis base and a carbenium centre as Lewis acid at a W^II^ alkyne complex turned out to be stable as a diradical. Intramolecular cyclisation and subsequent deprotonation provided a side‐on complex of a λ^5^‐1‐phospha‐2,3‐didehydrofluoranthene, which can be denoted as λ^5^‐phosphinyne.

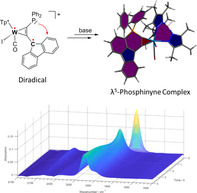

## Introduction

Arynes are highly reactive 1,2‐didehydroarene intermediates that can undergo various transformations in organic syntheses of specific arenes.^1^ The very elusive arynes can be trapped by metal coordination in side‐on complexes, which serve as proof of identity and model case as well as for preparative purposes.^2^ Aryne complexes are usually formed from suitable precursors at a metal template. Typical preparative approaches comprise thermal elimination of hydrocarbons from phenyl metal derivatives,^3^ metal‐assisted reductive elimination of halogen or triflate and boronic ester substituents^4^ or metathesis following double deprotonation.^5^

**Figure 1:**
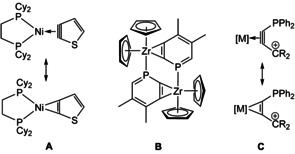
Heteroaryne complexes: thiophyne complex **A**, phosphinyne complex **B** and targeted side‐on complexes of phosphine carbenium substituted alkynes **C**.

Heteroaryne complexes represent an interesting version of aryne complexes, because such compounds have an additional functionality available for example, for tuning of electronical properties or for expanding heteroarenes by cycloaddition reactions.^6^ However, preparative access to heteroaryne complexes has been proven challenging due to the competing coordination via the heteroatom. Recently, Nishii, Miura and co‐workers presented first thiophyne complexes **A** (Figure 1), which contain an even more strained five‐membered aryne ring.^7^ The only structurally characterized example of a phosphinyne (phosphabenzyne) complex, published by Mattey and Le Floch, is the dimeric zirconocene complex **B**, in which a λ^3^‐phosphinyne acts as a bridging ligand in a κ^1^‐*P*‐η^2^‐*C*,*C’* coordination mode.^8^ Hence, λ^5^‐phosphinynes are attractive goals for the selective formation of side‐on complexes. While the coordination chemistry of λ^3^‐phosphinines is well established and used in catalysis,^9^ λ^5^‐congeners show limited metal coordination, because the P‐atom is blocked. Known examples show either η^5^‐coordination of M(CO)_3_ units (M=Cr, Mo, W)^10^, ^11^ or Pd/X‐addition (X=Cl, alkyl) at a pincer type λ^3^‐phosphinine.^12^

In this context, our research is focused on alkyne complexes bearing heteroatom substituents. Alkyne complexes of W^II^ with two donor centres like thiolates, amides or phosphines are suitable platforms for the formation of polynuclear complexes.^13^, ^14^, ^15^ In regard of a metal‐template‐based phosphinyne complex synthesis we got interested in the combination of a phosphine and a carbenium substituent at the coordinated alkyne (**C**). In addition, this assembly seemed attractive as a potential frustrated Lewis pair (FLP).

**Scheme 1:**
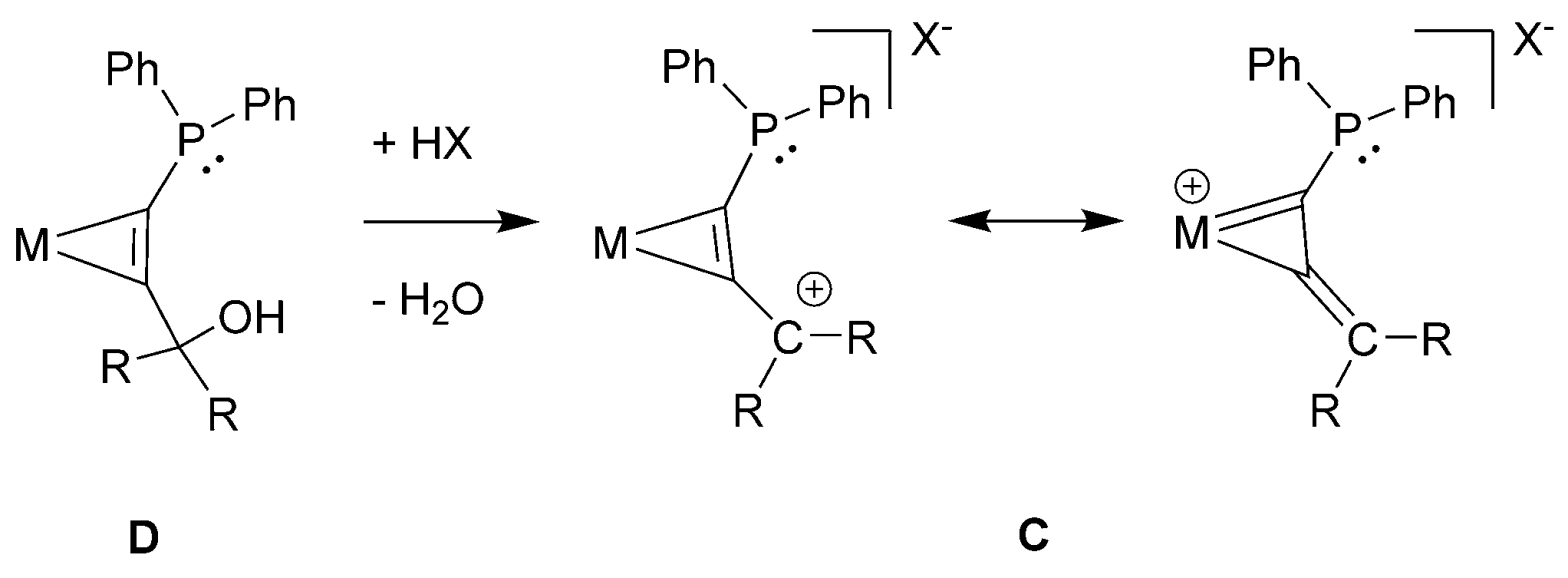
Proposed generation of an intramolecular phosphine carbenium combination at a coordinated alkyne.

Since the discovery of the remarkable activation potential of combinations of sterically hindered boranes and phosphines in 2006 by Stephan and co‐workers, the concept of frustrated Lewis pairs (FLPs) has developed to an essential innovation in inorganic chemistry.^16^ The proof of catalytic activity of these species for example, in polar double‐bond hydrogenation^17^ underscore the potential of FLPs for metal‐free homogeneous catalysis. Group 14 elements were implemented either by N‐heterocyclic carbenes as Lewis base^18^ or by using electron poor allenes as Lewis acid into FLP chemistry.^19^ Müller and co‐workers succeeded in dihydrogen activation by triarylphosphines and silylium cations, the latter being obtained in an elegant substituent transfer reaction.^20^ However, Stephan and co‐workers reported that the lighter homologous CPh_3_^+^ (trityl) cation react with sterically encumbered phosphines by nucleophilic attack of phosphines at *para*‐position of one trityl based phenyl group. Depending on a sigmatropic rearrangement, different phosphonium salt derivatives were isolated. A constraint of flexibility between the phosphine and the carbenium centre by a metal coordinated alkyne could keep the Lewis pair at distance. In addition, conjugation with the metal was believed to stabilize the carbenium centre (Scheme 1). Alternatively, an intramolecular cyclization would offer a facile avenue to phosphinyne complexes.

In this conjunction, recent reports by the research groups of Stephan and Müller emphasized the importance of radical reactions following a preceding single electron transfer (SET) in FLP chemistry.^21^ Frustrated radical pairs [Mes_3_P^**⋅**^]^+^[^**⋅**^B(C_6_F_5_)_3_]^−^ have been proven in phosphine borane combinations.^22^ A similar behaviour has been demonstrated by Müller and co‐workers for related silylium and trityl ion phosphine combinations. Silylium ion phosphine pairs show redox equilibria, while the coexistence of sole trityl and phosphinyl radicals in solution was nicely shown by EPR spectroscopy.^23^

In this contribution we report on the formation of covalently linked phosphine carbenium ion combinations in a W^II^ alkyne complex. Comprehensive reactivity studies show that an intramolecular cyclisation reaction prevails over FLP type activation processes. The final product represents an unprecedented side‐on aryne complex of a λ^5^‐phosphinyne. The intramolecular cyclisation reaction is, depending in the substituents used, slow enough to trace intermediates spectroscopically and to clarify the reaction mechanism and in particular, the role of open‐shell states for the phosphinyne complex formation.

## Results and Discussion

**Scheme 2:**
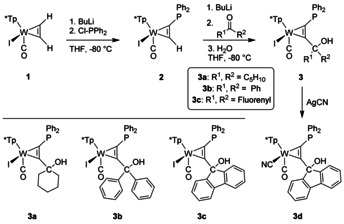
Synthesis of η^2^‐C,C’ phosphino propargyl complexes with iodide and cyanide as co‐ligands.

Dehydration of a suitable phosphino‐η^2^‐*C*,*C*’‐propargyl complex **D** caused by strong acids should lead to the generation of a metal stabilised carbocation **C** (Scheme 1). Due to the propensity of phosphine groups to metal coordination direct preparative use of suitable free alkynes is not feasible, making a stepwise assembly of the ligand at the metal template necessary. According to Scheme 2 we recently established a strategy for the selective introduction of phosphine groups into coordinated acetylene in [Tp*W(CO)I(η^2^‐C_2_H_2_)] **1** (Tp*=hydridotris(3,4,5‐trimethylpyrazolyl)borate).^14^ Deprotonation of **1** with *n*BuLi at −78 °C and subsequent trapping with ClPPh_2_ led to **2**. According to ^1^H and ^31^P NMR spectroscopy (δ_H_=13.4 ppm for the alkyne‐H atom and δ_P_=18.4 ppm) a single isomer was obtained showing the phosphine in the proximity to the pyrazole pocket. The modular character allowed the consecutive introduction of a second electrophile. Deprotonation of complex **2** with *n*BuLi at −78 °C caused the green solution to turn deep blue. Subsequent addition of a ketone resulted within minutes to hours in a green solution, which was quenched with water at low temperatures. After chromatographic purification and recrystallization, products **3 a**–**c** could be obtained in good yields. The cyanide derivative **3 d** was obtained by subsequent metathesis reaction with **3 c** and AgCN. Interestingly, the pure alkyne derivatives with fluorenyl and biphenyl moiety were already known in the literature, but never used as a ligand before.^24^

The ^13^C NMR resonances of the metal coordinated alkynes in **3 a**–**d**, which were detected in the range 204 to 227 ppm, prove the 4 e^−^ donor character of the alkynes.^25^ The influence of substituents at the α‐carbon atom is less noticeable in ^31^P NMR, because the shifts of all compounds were found between 15 and 16 ppm. However, the CO stretching modes in the IR spectrum ranging from 1923 to 1933 cm^−1^ are more indicative for differences at tungsten.

**Figure 2:**
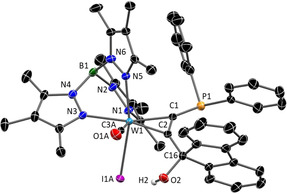
Molecular structure of **3 c** in the crystal (50 % thermal ellipsoids). With the exception of OH hydrogen atoms have been omitted for clarity. Selected bond lengths (Å) and angles (°):W1−C1 2.055(3), W1−C2 2.059(2), W1−C3A 1.972(3), P1−C1 1.785(3), C1−C2 1.309(4), C2−C16 1.515(4), P1−C16 3.866, C2‐C1‐P1 141.0(2), C1‐C2‐C16 140.3(2).

The molecular structures of all complexes **3 a**–**d** were determined by single‐crystal XRD analysis (see Supporting Information). As an example, structure **3 c** is depicted in Figure 2. The molecular structures share most structural features among each other and with related examples.^25^, ^26^ Even the substitution of I^−^ (**3 c**) by CN^−^ (**3 d**), which is crucial with respect to electronic behaviour (vide infra), causes only a slight contraction of the W−C1/C2 bond lengths, which is on the significance limit. As with all derivatives, the hydroxyl group is not directed to the phosphine, but in the opposite direction. The distances between C16 and P1 amount to about 3.87 Å, which is comparatively large in relation to the 2.18–2.20 Å in covalently linked borane phosphine‐based FLPs.^27^

**Scheme 3:**
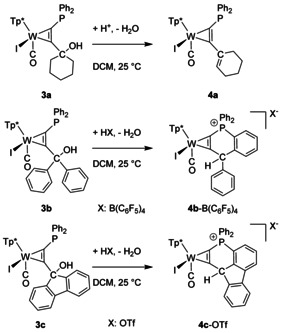
Reaction of complexes **3 a**–**c** with one equivalent of a strong acid.

**Figure 3:**
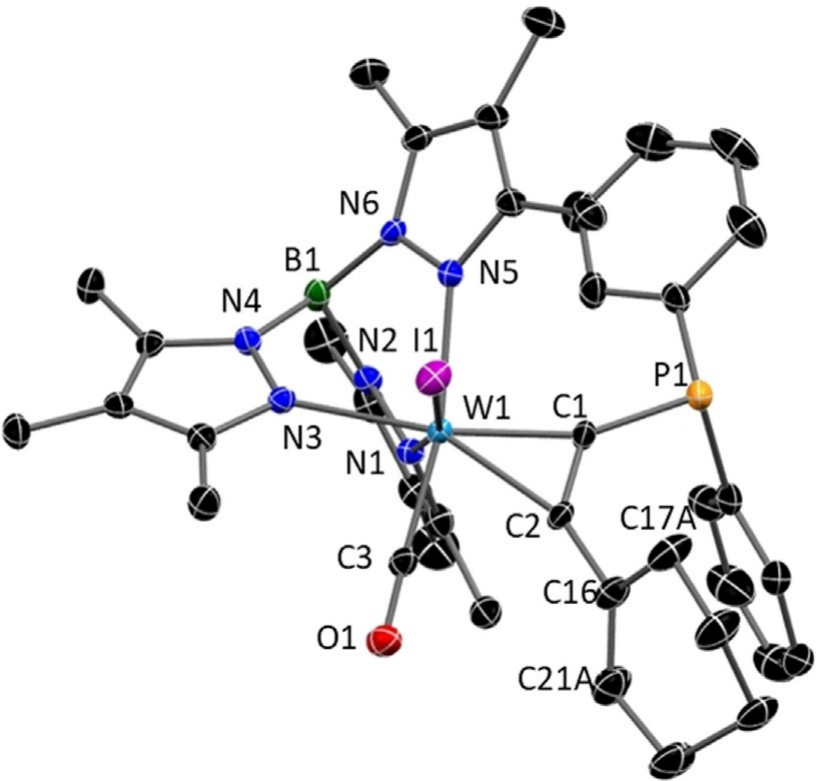
Molecular structure of **4 a** in the crystal (50 % thermal ellipsoids). Hydrogen atoms omitted for clarity. Selected bond lengths (Å) and angles (°): **4 a**: W1−C1 2.035(4), W1−C2 2.047(4), W1−C3 1.952(4), P1−C1 1.795(4), C1−C2 1.322(6), C2−C16 1.468(5), C16−C21A 1.336(17), C16−C17A 1.510(15), C2‐C1‐P1 129.7(3), C1‐C2‐C16 136.5(4).

For the generation of a carbenium group we tested different acids of weakly coordinating anions. Besides HBF_4_⋅Et_2_O and triflic acid (HOTf) the Brookhart type acid [H(Et_2_O)_2_][(B(C_6_F_5_)_4_] were applied successfully.^28^ When added dropwise, the colour changed from dark green over red to finally light green. According to our observations the red solution showed prolonged lifetime with increasing size of the anion. The spontaneous dehydration after protonation even at low temperature was shown by the cyclohexanol derivative **3 a**, which however yielded neutral **4 a** bearing a cyclohexene residue (Scheme 3). The crystals of **4 a** do not contain an anion and the molecular structure shows a typical double bond C16−C21A of 1.336 Å (Figure 3). The diphenyl derivative **3 b** was chosen to preclude this process and to provide an improved stabilization of the positive charge at the carbenium centre. After several hours at room temperature IR spectroscopy indicated a final product **4 b**‐B(C_6_F_5_)_4_, exhibiting a CO band at increased frequency of 1975 cm^−1^(**3 b**: 1933 cm^−1^).

**Figure 4:**
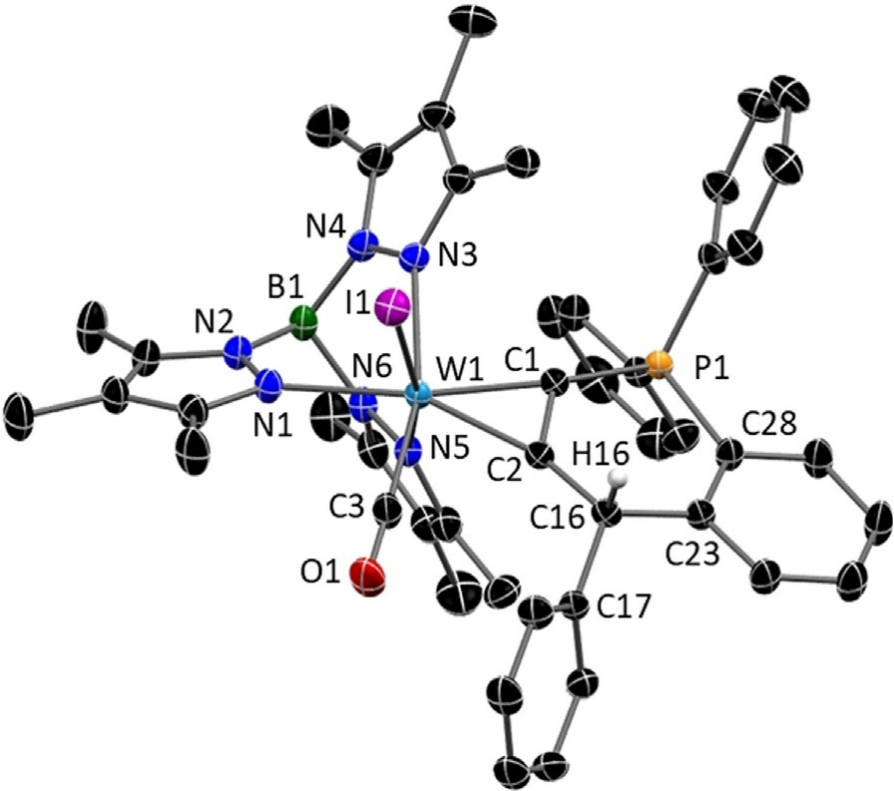
Molecular structure of **4 b**‐B(C_6_F_5_)_4_ in the crystal (50 % thermal ellipsoids). Hydrogen atoms and anion have been omitted for clarity. Selected bond lengths (Å) and angles (°): **4 b**‐B(C_6_F_5_)_4_: W1−C1 2.0240(17), W1−C2 2.0615(17), W1−C3 1.984(2), P1−C1 1.7440(18), P1−C28 1.7864(18), C23−C28 1.405(2), C16−C23 1.537(2), C2−C16 1.502(2), C1−C2 1.332(2), C2‐C16‐C17 117.21(14), C2‐C16‐C23 112.34(14), C17‐C16‐C23 114.06(14).

A single crystal XRD analysis of **4 b**‐B(C_6_F_5_)_4_ uncovered a cyclic structure including a λ^4^‐phosphonium centre (Figure 4). Apparently the carbenium ion was attacked by the phosphine at one phenyl group in *ortho*‐position followed by a sigmatropic rearrangement. The ^31^P NMR resonance shifted to 10 ppm, and the signal of the transferred H atom was detected in ^1^H NMR at 6.5 ppm as a doublet with ^4^ *J*_PH_=4.7 Hz. According to the molecular structure of **4 b** ^+^ the alkyne coordination at tungsten is largely retained. As a result of the cyclisation, the C1−P1 bond length has decreased from 1.785(3) in **3 b** to 1.744(2) in **4 b** ^+^. Unequivocally, the isolation of **4 b**‐B(C_6_F_5_)_4_ is indicative of the intermediate carbenium/phosphine derivative sought‐after.

**Figure 5:**
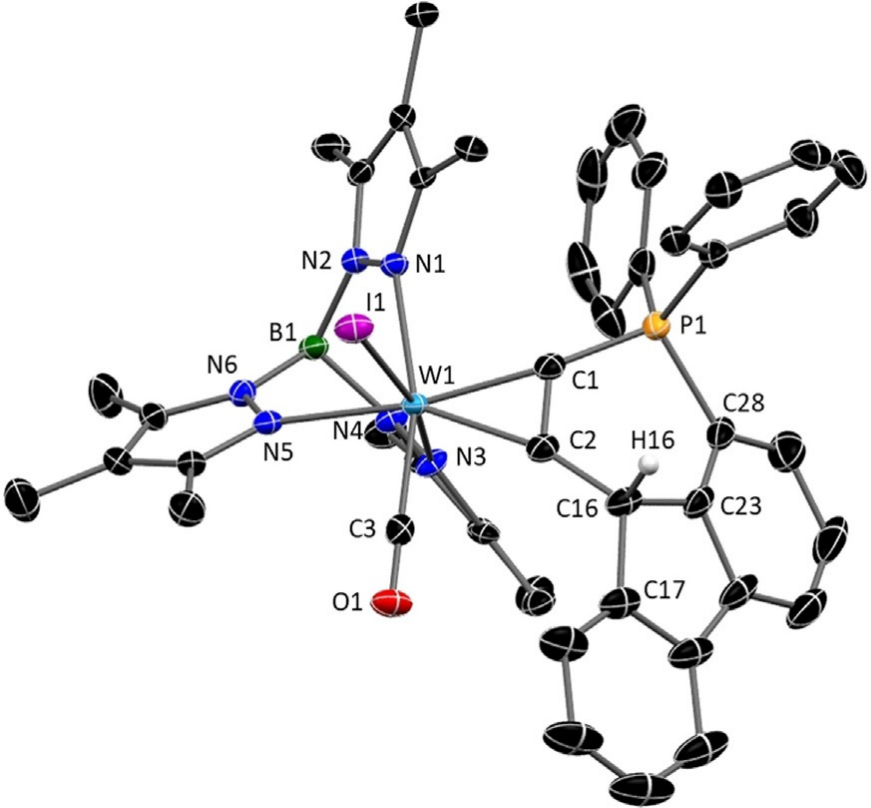
Molecular structure of complex cation **4 c^+^** in the crystal (50 % thermal ellipsoids); hydrogen atoms and anion (OTf^−^) have been omitted for clarity. Selected bond lengths (Å) and angles (°):W1−C1 2.009(4), W1−C2 2.050(4), W1−C3 1.980(4), P1−C1 1.753(4), P1−C28 1.808(4), C23−C28 1.386(7), C16−C23 1.517(7), C2−C16 1.501(6), C1−C2 1.341(6), C2‐C16‐C17 124.8(4), C2‐C16‐C23 107.0(3), C23‐C16‐C17 103.4(4).

In the course of experiments to extend the lifetime of the open phosphine/carbenium Lewis pair, we implemented the fluorenyl derivative **3 c**, because reduced flexibility by additional fixation of the two phenyl rings should impede intramolecular phosphine attack. Accordingly, ^31^P NMR monitoring after addition of [H(Et_2_O)_2_][(B(C_6_F_5_)_4_] to **3 c** revealed a reduced cyclisation rate indeed (vide infra, Figure 8, bottom), but basically a comparable conversion. Thus, single crystal XRD analysis of the final product **4 c**‐OTf disclosed that the cyclic phosphonium compound was formed also in this case (Figure 5, Scheme 3). Essential differences between **4 b** ^+^ and **4 c** ^+^ are evident in their molecular structures in the solid state. While the geometry about the tertiary carbon atom in **4 b** ^+^ (343.6° angular sum about C16H) is roughly tetrahedral, angles of 103.5° to 124.8° about C16H in **4 c** ^+^ reflect a severe distortion towards a planar ring structure.

**Scheme 4:**
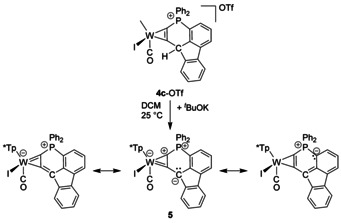
Generation and main resonance structures of complex **5**.

**Figure 6:**
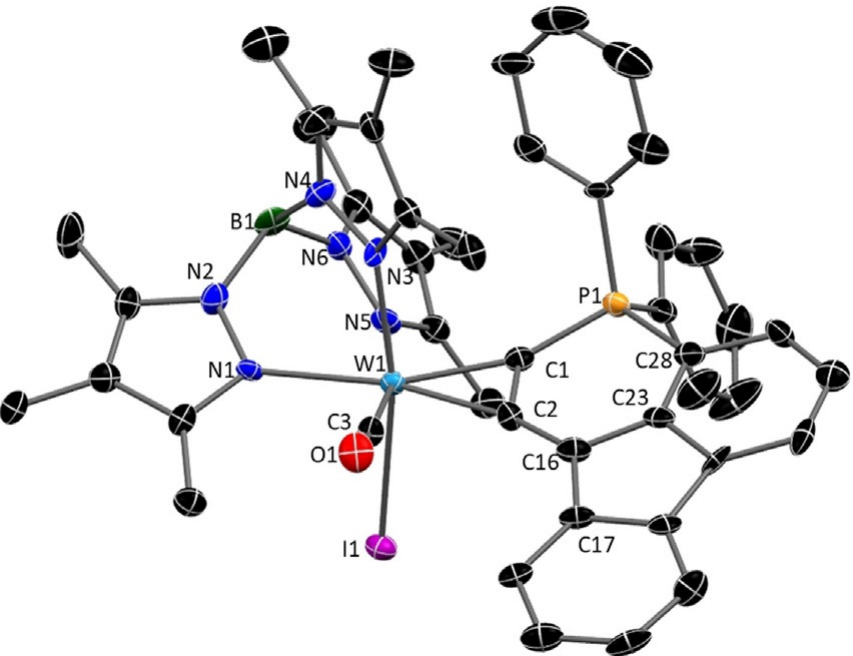
Molecular crystal structure of the neutral complex **5** (50 % thermal ellipsoids). Hydrogen atoms have been omitted for clarity. Selected bond lengths (Å) and angles (°): W1−C1 1.993(10), W1−C2 2.102(9), W1−C3 1.960(8), C1−P1 1.743(10), P1−C28 1.772(10), C23−C28 1.395(15), C16−C23 1.426(15), C2−C16 1.401(13), C1−C2 1.383(13), C2‐C16‐C17 135.4(10), C2‐C16‐C23 116.3(9), C23‐C16‐C17 108.1(9).

In agreement with this distortion, complex **4 c** ^+^ turned out to be acidic enough to yield a stable deprotonation product. Treatment of a yellow solution of **4 c** ^+^ with *t*BuOK (Scheme 4) led to a colour change to deep green, which is accompanied by a change of the CO band from 1975 cm^−1^ for **4 c** ^+^ to 1903 cm^−1^ and a relatively small change of the ^31^P NMR shift from 12.9 ppm to 10.9 ppm. The neutral product **5** is sufficiently stable to allow chromatography and crystallization from CH_2_Cl_2_ solution. The result of the single crystal XRD measurement with **5** is depicted in Figure 6. The molecular structure clearly shows the planarization of the metal coordinated C_5_P ring system induced by a planar environment at C16. The angle sum around C16 amounts to 359.8°, whereas the strongest deviation from 120° applies to C2‐C16‐C17 with 135.4°. The C−C bond lengths within the ring fall between 1.383 and 1.426 Å, which is consistent with the formation of a π‐conjugated C_5_ system. Although, the C1−P1 bond length of 1.743(10) Å comes within the limits between a P−C single bond (1.790 Å) and a P−C double bond (1.661 Å), there is no significant shortening in comparison to **4 c** ^+^.^29^ In contrast, the difference of bond lengths between W1−C1 and W1−C2 has significantly increased going from **4 c** ^+^ to **5**. The latter is clearly reflected in the resonance structures of complex **5** (Scheme 4), which is therefore best described as a side‐on λ^5^‐phosphinyne complex.

**Figure 7:**
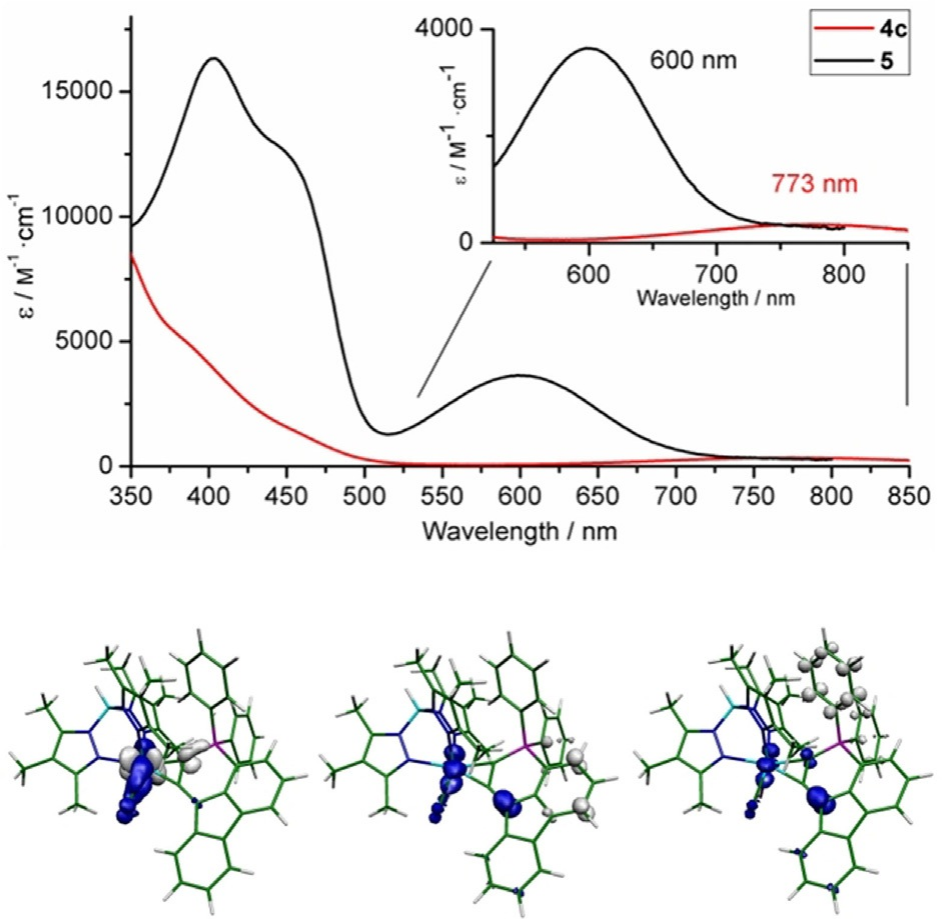
UV/Vis spectra in CH_2_Cl_2_ of **4 c^+^** (red) and compound **5** (black); difference electron density (drop blue, gain white) for the three main visible transitions calculated by TD‐DFT (Figure S44).

Besides the structural data, the spectroscopic parameters are in accordance with such of λ^5^‐phosphinines.^11^ The zwitterionic nature of λ^5^‐phosphinines emphasized already by Dimroth^11^, ^30^ is indicated by the significant decrease of the CO stretching frequency by around 70 cm^−1^ resulting from the deprotonation. As reflected in the resonance structures, the negative charge is rather distributed from the original deprotonation site to the metal centre. Leading resonance structures regarding the W phosphinyne bonding situation depicted in Scheme 4 are derived from a natural resonance theory analysis (Figure S46). This notion is supported by the visible absorption spectra and its assignment based on TD‐DFT calculations. The visible absorption feature, which shifted from 773 nm for **4 c** ^+^ to 600 nm for **5**, exhibited a tenfold increase of the molar extinction coefficient from 353 to 3650 m ^−1^⋅cm^−1^ (Figure 7). This transition exhibits mainly dd‐character, which is significantly mixed with metal to ligand character in **5** explaining the observed absorptivity gain. The two high intensity absorptions at 405 and 450 nm can be assigned to transitions with ligand to ligand mixed with metal to ligand character from the negatively polarized WC_2_C moiety to the positively polarized phosphonium site (see Figure 7 bottom).

### Cyclization mechanism

**Figure 8:**
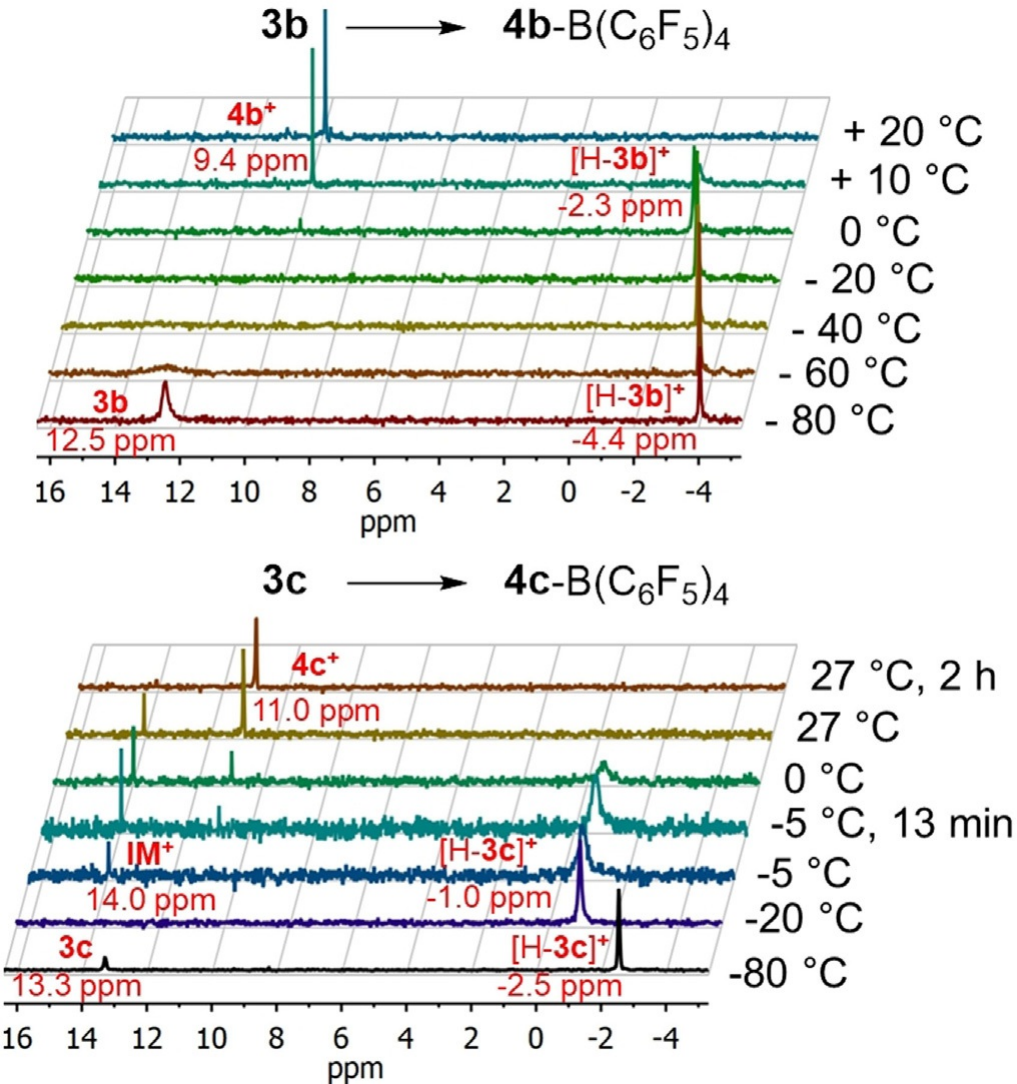
Reaction sequence of **3 b** (top) and **3 c** (bottom) with [H(Et_2_O)_2_][(B(C_6_F_5_)_4_] monitored by ^31^P NMR spectroscopy. Reaction mixtures were warmed stepwise from −80 °C to room temperature. Note the slight temperature shift of species [H‐**3 b**]^**+**^/[H‐**3 c**]^**+**^, which is characterized by a doublet in ^1^H coupled ^31^P NMR spectra ([H‐**3 b**]^**+**^: *δ*=−4.4 ppm, ^1^ *J_PH_*=527 Hz; [H‐**3 c**]^**+**^: *δ*=−2.5 ppm, ^1^ *J_PH_*=501 Hz).

To get insights in the cyclization mechanism the reactions were monitored upon warming by ^31^P NMR spectroscopy (Figure 8). Immediately after addition of acid at −80 °C, new signals at −4.4/−2.5 ppm in addition to the signals of the starting materials **3 b**/**3 c** at 12.5/13.3 ppm were detected, respectively. Measurement of a H‐coupled spectrum of [H‐**3 b**]^+^ revealed a doublet with a coupling constant of 527 Hz for the signal at −4.4 ppm, proving preferential protonation at phosphorus. Temperature‐ and time‐dependent measurements with [H‐**3 b**]^+^ did not allow to identify any signal for potential intermediates besides the signal of product **4 b**‐B(C_6_F_5_)_4_ at 9.4 ppm. In contrast, the related experiment with **3 c** revealed a reduced cyclisation rate (Figure 8, bottom). Consequently, in addition to [H‐**3 c**]^+^ protonated at phosphorus and the final product **4 c** ^+^, an intermediate **IM** could also be detected at 14.0 ppm, which persisted for several hours.

All attempts to trap the intermediate phosphine/carbenium Lewis pair with different substrates such as H_2_, C_2_H_2_, CO_2_ or PhSiH_3_ did not succeed, which underlines the high preference for an intramolecular cyclization. For the diphenyl derivative **3 b** this observation might be explained by the high reactivity of the intermediates. However, a lifetime in the range of hours for the intermediate **IM** detected in the cyclization with the fluorenyl compound **3 c** seemed conflicting, which prompted us to further studies.

**Figure 9:**
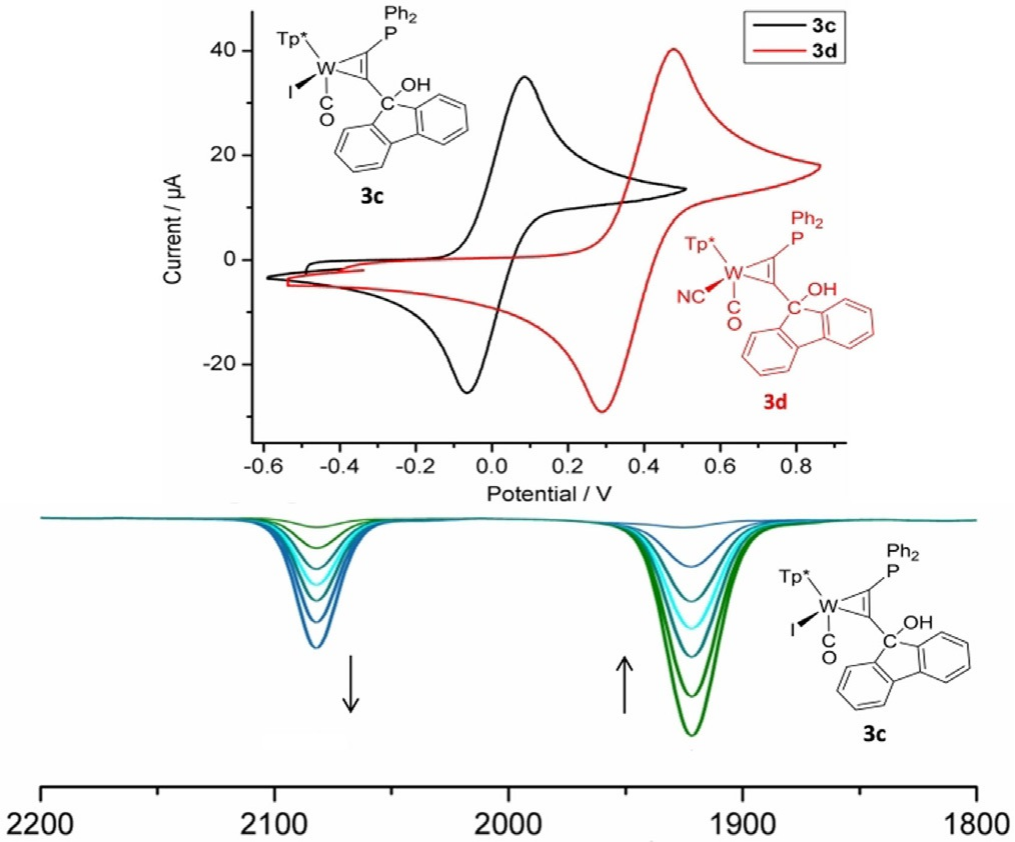
Cyclic voltammetry of complex **3 c** (black) and **3 d** (red) measured in CH_2_Cl_2_ (referenced against ferrocene/ferrocenium); oxidation process W(II/III) for **3 c** at *E*_1/2_=+0.01 V and for **3 d** *E*_1/2_=+0.38 V); IR spectro‐electrochemical measurement confirmed the tungsten based oxidation.

**Figure 10:**
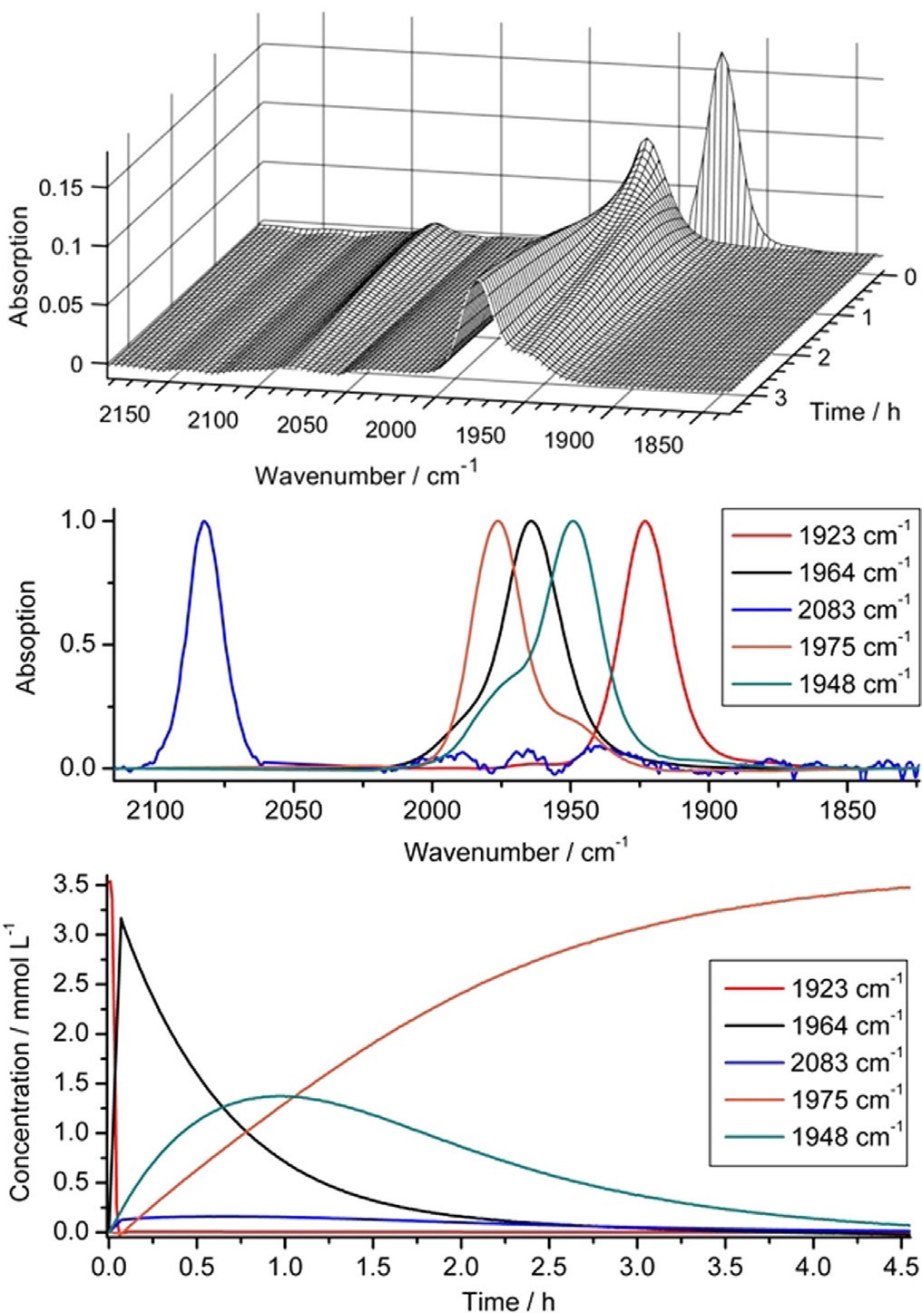
Series of measured IR spectra from the reaction monitoring of **3 c** with [H(Et_2_O)_2_][(B(C_6_F_5_)_4_] in the range of carbonyl vibration (top). Decomposition of the mixture spectra into the pure component spectra (centre) and the corresponding concentration profiles (bottom). The pure component spectra are scaled to a maximum height of 1.

In the first place we performed time‐dependent in situ IR spectroscopy at room temperature. In particular, appearance of CO vibration bands allows conclusions on potential intermediates. However, interpretation of the IR spectra was complicated by overlapping bands for a number of intermediates. In addition to the known band of the starting material **3 c** at 1923 cm^−1^, the band of the product **4 c** ^+^ at 1975 cm^−1^ is clearly visible (Figure 10, top). The essential remaining bands of intermediates could not directly be assigned. Nevertheless, the transient appearance of a separate CO band at 2083 cm^−1^ is indicative of a W^III^ species. This conclusion was proven by an IR spectro‐electrochemical measurement with **3 c**. The W based oxidation is reflected by an increase of the CO vibration from 1923 to 2083 cm^−1^ (Figure 9).

The decomposition of such a series of multicomponent spectra can be done by the multivariate curve resolution method (MCR).^31^ The data shown in top of Figure 10 were stored in a matrix $D\in {\mathbb{R}}^{m\times n}$ on a time×frequency grid. Thus the rows of D contain the spectra at the m measurement times and each of these spectra is taken at n wavenumbers. Let s be the number of chemical components. Then $C\in {\mathbb{R}}^{m\times s}$ is the matrix of the concentration profiles and $S\in {\mathbb{R}}^{n\times s}$ the matrix of the associated pure component spectra. We are interested in determining C and S in a way that $D=CS^{T}$ (representing the Lambert‐Beer law) holds. Here we used a combination of the peak group analysis (PGA)^32^ in order to extract the starting material (1923 cm^−1^) and further a kinetic hard modelling approach to decompose the remaining system.^33^ The extracted information is shown in the centre and bottom plots of Figure 10. The extracted pure component spectra clearly show besides to the band of **3 c** (1923 cm^−1^) and the product **4 c^+^** (1975 cm^−1^), two intermediates (1964 cm^−1^, 1948 cm^−1^). Unexpectedly, together with the W^III^ intermediate detected at 2083 cm^−1^, five different species are indicated; hence one species more as found in ^31^P NMR.

**Scheme 5:**
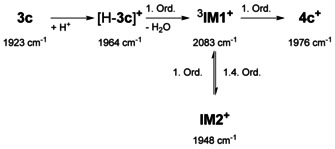
Derived kinetic model that describes the reaction mechanism.

In order to provide a reasonable assignment in consideration of possible concentration‐time curves, these were compared with potential kinetic models. The results showed that linear reaction paths with five components do not optimally represent the measured data. Only the integration of a dead‐end‐state **IM2** ^+^, with one fractional reaction order in equilibrium with **IM1** ^+^ leads to a conclusive agreement (see Scheme 5). Because of unknown intermediate reaction steps numerous plausible reaction models have been studied. The kinetic hard‐modelling approach has been applied for each model (see Supporting Information). The results have been compared regarding the reconstruction error of the spectral mixture data and the error of the model fit. The following model is optimal in terms of these two indicators among all tested models.

The IR band at 1964 cm^−1^ can be assigned to species [H‐**3 c**]^+^, which represents the protonated phosphine detected unequivocally in ^31^P NMR. Compound [H‐**3 c**]^+^ is rapidly formed after proton addition and the change by 41 cm^−1^ is consistent with formation of a terminal phosphonium group. The CO band at 2083 cm^−1^ being indicative of paramagnetic W^III^ is assigned to intermediate ^3^ **IM1** ^+^ after dehydration. Intramolecular oxidation of W^II^ by the terminal carbenium centre could lead to a biradical or triplet state which is NMR silent.^15^, ^34^, ^35^, ^36^

The existence of a tungsten‐based *S=*1/2 system was confirmed by an EPR experiment. The X band spectrum in solution (see Supporting Information, Figure S1) displayed an isotropic hyperfine coupling A_I_=26×10^−4^ cm^−1^ to ^127^I (*I*=5/2, 100 %) centred at <*g*>=2.003, while signs for an additional organic radical were missing. This observation can be explained by dimerization of the biradical species as in the case of the Gomberg radical.^37^, ^38^ The observation of a pure tungsten based *S=*1/2 system in the EPR experiment does not exclude the existence of a monomer/dimer equilibrium, since a real triplet state should be largely EPR silent due to zero field splitting.

**Scheme 6:**
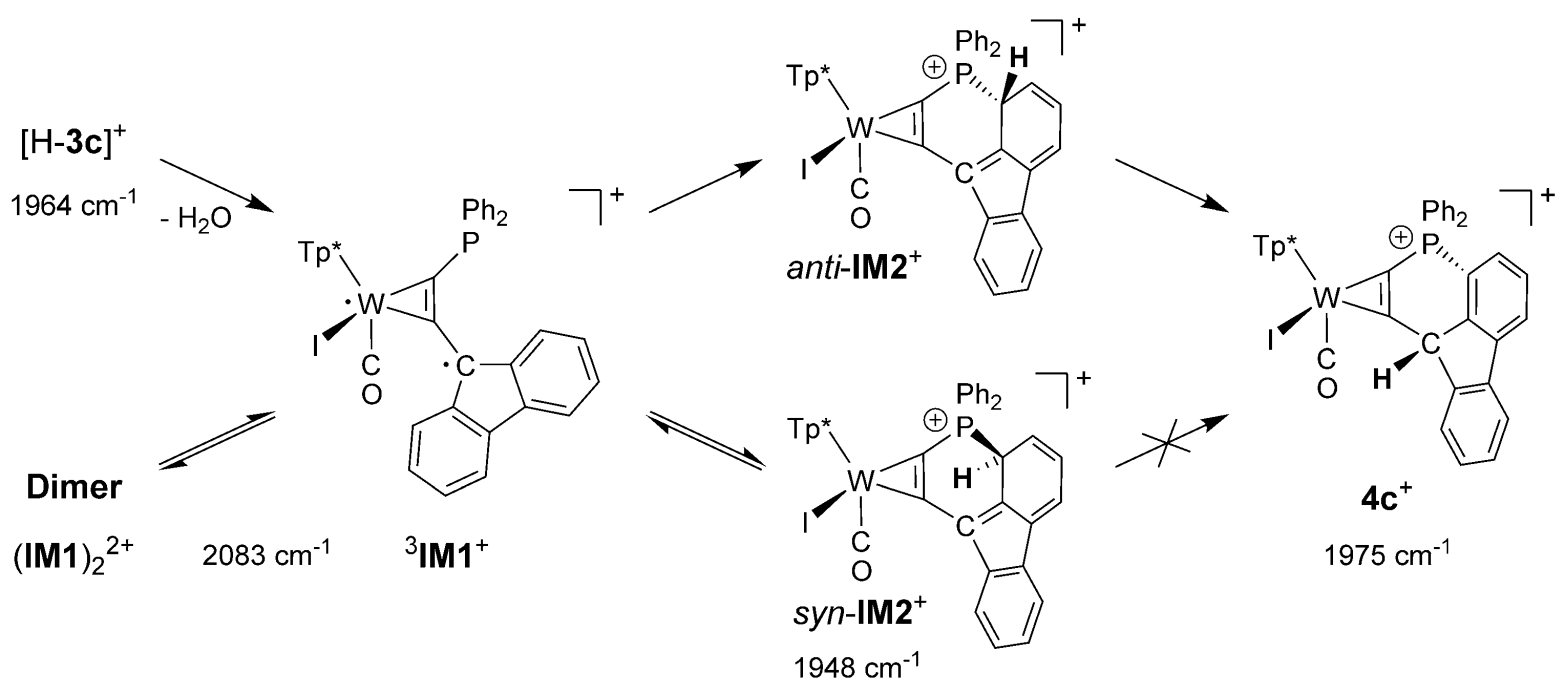
Reaction sequence of [H‐**3 c**]^+^ to **4 c^+^**. After dehydration biradical ^**3**^ **IM1^+^** is at equilibrium with its dimer **(IM1)_2_** ^**2+**^, as confirmed by EPR spectroscopy. Cyclisation of the monomer ^**3**^ **IM1^+^** results two isomers, in which only ***anti*** **‐IM2^+^** can lead to the product by a suprafacial sigmatropic rearrangement. For ***syn*** **‐IM2^+^** the antarafacial rearrangement is impeded (the kinetic dead end).

**Figure 11:**
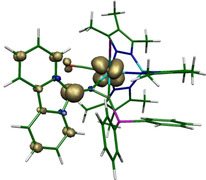
Mulliken spin density distribution of intermediate ^3^ **IM1** ^+^ (CAM‐B3LYP, def2‐TZVP, cut‐off 0.01).

In search for the spin state of the FLP type product after dehydration of [H‐**3 c**]^+^ and for the identity of the intermediates detected in the IR spectroscopic monitoring DFT calculations were carried out. Geometry optimizations for the fluorene derivatives were performed using hybrid functionals without and with long‐range contribution taking into account (PBE0 and CAM‐b3lyp) and TZVP basis sets. Results are compiled in Table 1 with reference to Scheme 6. Comparison of the singlet and triplet state for the FLP derivative **IM1** ^+^ revealed a distinctive higher stability of the triplet state. The Mulliken spin density distribution of this ^3^ **IM1** ^+^ state depicted in Figure 11 is distributed over the central metal (0.95) and the fluorenyl system. This electronic state represents a W^III^ redox state displaying the highest calculated CO frequency.

**Table 1:** DFT calculation results Δ*H/*kcal mol^−1^ and *ν*(CO)/ cm^−1^ for reaction intermediates and *ν*(CO) for the dimer intermediate model ^2^[H‐**IM1**]^+^.

Compound^[a]^	PBE0		CAM‐B3LYP	
	Δ*H* ^[b]^	IR (CO)^[d]^	Δ*H* ^[b]^	IR (CO)^[d]^
^3^ **IM1** ^+^	0	2155	0	2197
^1^ **IM1** ^+^	+8.9		+11.3	
^2^[H‐**IM1**]^+[c]^		2155		
*syn*‐**IM2** ^+^	+1.2	2092	+2.0	2113
*anti‐* **IM2** ^+^	+6.4		+7.1	
**4 c** ^+^	−21.4	2101	−23.2	2122

[a] See Scheme 6, *syn*/*anti*‐position relating to phosphorus and iodide. [b] Sum of total electronic energy (def2‐TZVP) and thermal correction to enthalpy (def2‐SVP). [c] Model for the dimer (**IM1**)_2_^2+^. [d] Without any correction.

Potential intermediates of the observed cyclisation are the direct products of bond formation between the fluorenyl group in 1‐position and phosphorus. Singlet species **IM2** ^+^ was calculated (Scheme 6), provided that the C−P bond formation is accompanied by re‐reduction of W^III^. Resulting from the chiral nature of the W alkyne complex two diastereomeric forms *syn*‐**IM2** ^+^ and *anti*‐**IM2** ^+^ are possible denoting the positions of the phosphine and iodine with respect to the formed cycle. The *syn*‐**IM2** ^+^ isomer is only marginally less stable than the ^3^ **IM1** ^+^ while both *syn*‐**IM2** ^+^ and *anti*‐**IM2** ^+^ are more stable than a singlet FLP state ^1^ **IM1** ^+^. The final sigmatropic rearrangement leading to diastereomerically pure **4 c** ^+^ is clearly exothermic. In addition, as a model system for the dimer (**IM1**)_2_^2+^, which could account for the EPR signal, ^2^[H‐**IM1**]^+^ bearing an added H atom at 3‐position of the fluorenyl rest was calculated. The CO vibration frequencies derived for ^2^[H‐**IM1**]^+^ are almost identical to those of ^3^ **IM1** ^+^, reflecting again the radical character at the fluorenyl moiety in ^3^ **IM1** ^+^. In consequence, a conceivable equilibrium ^3^ **IM1** ^+^/(**IM1**)_2_^2+^ cannot be resolved by IR spectroscopic monitoring. The broken reaction order of 1.4 for the formation of *syn*‐**IM2** ^+^, obtained by the kinetic modelling, can be attributed to the fact that the IR band at 2083 cm^−1^ represents both ^3^ **IM1** ^+^ and (**IM1**)_2_^2+^.

The final product **4 c** ^+^ exhibits iodide and the H atom on the same side of the formed cycle. Hence, formation of **4 c** ^+^ starting with *syn*‐**IM2** ^+^ can be excluded, because an antarafacial shift in the closed ring system is very unlikely. In contrast, *anti*‐**IM2** ^+^ can be converted into **4 c** ^+^ by a suprafacial 1,3‐rearrangement. According to the Woodward–Hoffmann rules this [1,3]‐shift can proceed either by a number of thermic [1,5]‐shifts or a direct [1,3]‐shift driven by light. The fact that the more stable isomer *syn*‐**IM2** ^+^ is unproductive is reflected in the kinetic dead end, while less stable *anti*‐**IM2** ^+^ leads in a fast, non‐rate‐determining step to **4 c** ^+^. This mechanistic conception potentially explains missing FLP activity of the intermediate species despite of their comparatively long lifetime.

With the knowledge of the reaction behaviour of **3 b**/**3 c**, we tried to make target‐oriented changes to the system that would allow the formation of a complex based FLP. By increasing the oxidation potential, the formation of a W^III^ species by the carbenium centre should be prevented. By substitution of the iodide ligand by cyanide we achieved a substantial increase of oxidation stability.^35^ The exchange was carried out with AgCN in ethyl propionate for 24 h under reflux conditions. After purification by column chromatography and recrystallization, the product **3 d** was obtained in good yields (Scheme 2). Cyclic voltammetry displayed a clear change of the redox potential from 10 mV for **3 c** to 380 mV for **3 d** (Figure 9). Complex **3 d** was treated likewise with [H(Et_2_O)_2_][(B(C_6_F_5_)_4_] and the reaction was monitored by in situ IR spectroscopy. By an analogous decomposition of the series of IR spectra with the peak group analysis four components could be separated in the wavenumber range at about 1950 cm^−1^.^32^ The analysis clearly confirmed the absence of W^III^ species in the reaction solution (see supporting information). Nevertheless, even under 50 bar hydrogen atmosphere we could not observe any FLP‐typical reaction. In addition, we have not been able to clarify the final outcome of the reaction.

## Conclusions

We have successfully adapted the dehydration protocol for the synthesis of carbenium ions to an alkyne complex based system. The resulting combination of a phosphine and a transient carbenium centre as vicinal substituents at the W^II^ coordinated alkyne represents formally a frustrated Lewis pair. However, it has been shown by IR and EPR spectroscopy supported by spectroelectrochemistry that an intramolecular electron transfer from W^II^ to the carbenium centre occurs. This conclusion is in accordance with the results of DFT calculations, which prove the higher stability of the W^III^/R_3_C⋅triplet state compared with the W^II^ carbenium singlet state. In agreement with this conclusion, Müller and co‐workers have recently shown that the combination of trityl salts with sterically demanding phosphines result in a redox step as well.^23^ We did not observe any phosphorus based spin density, which is confirmed by the fact that the W^III^ complex [Tp*W(CO)I{η^2^‐C_2_(PPh_2_)_2_}](BF_4_) could be isolated and fully characterized.^34^ Accordingly, the W(II/III) redox potential lies between those of the carbenium ion and the phosphine.

The electron transfer causes an intramolecular cyclisation, involving nucleophilic attack of the phosphine at *ortho*‐position of one electrophilic phenyl‐carbon‐based radical followed by a sigmatropic H atom rearrangement. Temperature‐ and time‐dependent ^31^P NMR investigations disclosed that cyclisation rate depends from the substituent at the propargyl group. According to the molecular structures, the distortion about the α‐alkyl group at the alkyne towards planarity is strongest in the fluorenyl type system **4 c** ^+^. IR‐spectroscopic monitoring with the fluorenyl based system **4 c** ^+^ and decomposition of the multicomponent spectra by multivariate curve resolution proved the existence of intermediates, which are stable at room temperature in the course of hours. In addition, a stringent kinetic interpretation disclosed an equilibrium with a dead‐end intermediate, which serve as an explanation for the lack of any FLP reactivity despite the relatively long lifetime of this intermediate. Our results form a significant basis for future variants and developments of functional FLPs in an alkyne complex scaffold. A stabilization of the carbenium centre by electron‐donating substituents was impeded by the reduced electrophilicity of the respective keto derivatives (tetramethylurea, phenyl(piperidine‐1‐yl)methanone or 4,4'‐dimethoxybenzophenone), which did not react with deprotonated **2**. Eventually, a first example of a side‐on coordinated didehydro‐λ^5^‐phospinine, which can also be denoted as η^2^‐λ^5^‐phosphinyne complex was isolated and fully characterized. The remarkable stability and the substantial difference of the visible absorption behaviour for the Brønsted acid/base pair **4 c** ^+^/**5** provide the basis for the future development of a pH‐indicator for spectral pK_a_ determinations in organic solvents.

## Experimental Section

### Characterization of compounds

**Material and methods**: All operations were carried out in an atmosphere of dry argon using Schlenk and glovebox techniques. Solvents were dried and saturated with argon by standard methods and freshly distilled prior to use. [Tp*W(CO)I(HC_2_H)] (**1**) and [Tp*W(CO)I(Ph_2_PC_2_H)] (**2**) were prepared according to literature methods.^34^ NMR spectra were recorded at 300 K using Bruker Avance 250, 300 or 500 MHz spectrometers. In ^1^H and ^13^C NMR the chemical shifts are internally referenced to the solvent residual peak. The ^31^P/^19^F NMR chemical shifts are referred to H_3_PO_4_ (85 %) and CFCl_3_, respectively. IR‐spectroscopy was conducted on a Bruker Alpha T for pure components or a Bruker Tensor 27 with MCT‐detector for reaction mixtures. UV/Vis‐spectroscopy was conducted on an Agilent Cary 60 spectrophotometer; cyclic voltammetry and spectro‐electrochemical data were acquired on a Princeton Applied Research VersaSTAT 3. The optically transparent thin‐layer electrochemical (OTTLE) cell followed the design of Hartl and Winter.^38^, ^39^ Details on the X‐ray diffraction experiments, on the EPR measurement and the quantum chemical calculations are given in the Supporting Information.

### Syntheses

**Preparation of compounds 3 a**–**c**: A green solution of Tp*W(CO)(I)(Ph_2_PC_2_H) (**2**) (500 mg, 0.56 mmol) in THF (30 mL) was treated dropwise with *n*BuLi (2.5 m solution in *n*‐hexane, 0.3 mL) at −78 °C. The resulting dark‐blue solution was stirred for 5 min and a ketone solved in THF was added (**3 a**: cyclohexanone 1.5 equiv, **3 b** benzophenone 2.0 equiv, **3 c** fluorenone 2.0 equiv). A colour change to green was observed within minutes (**3 a**) and a solution of H_2_O (0.011 mL, 0.56 mmol) in THF was added. In case of **3 b**–**c** a solution of H_2_O (0.011 mL, 0.56 mmol) in THF was added to the dark‐blue solution after 7 h. After warming up, the volatiles were removed in vacuo. The green residue was purified by column chromatography on silica using a 1:1 mixture of petroleum ether and CH_2_Cl_2_ to remove unreacted **2** and ketone, before elution with 100 % CH_2_Cl_2_ yielded product **3 a**–**c**. The solvents were evaporated yielding a green powder (**3 a**: 319 mg, 58 %, **3 b**: 431 mg, 72 %, **3 c**: 426 mg, 71 %). Single crystals suitable for XRD analysis were obtained by slow diffusion of *n*‐pentane into a concentrated solution of **3 a** in CH_2_Cl_2_ or by layering a concentrated solution of **3 b**/**3 c** in CH_2_Cl_2_ with *n*‐pentane.

**3 a**: ^1^H NMR (300 MHz, CDCl_3_): *δ*=7.4–7.3 (m, 2 H, Ar‐*H*), 7.3–7.2 (m, 3 H, Ar‐*H*), 7.1 (t, ^3^ *J*_HH_=7.4 Hz, 1 H, Ar‐*H*), 6.9 (t, ^3^ *J*_HH_=7.0 Hz, 2 H), 6.6 (t, ^3^ *J*_HH_=8.1 Hz, 2 H), 3.7 (d, *J=*2.3 Hz, 1 H, O*H*), 2.8 (s, 3 H), 2.7 (s, 3 H), 2.5–2.3 (m, 2 H; C*H*_2_), 2.2 (s, 3 H), 2.2 (s, 3 H), 2.1 (d, *J=*2.3 Hz, 3 H), 1.9 (s, 3 H), 1.8 (s, 3 H), 1.8 (s, 3 H), 1.7–1.4 (m, 8 H; C*H*_2_), 1.4 (s, 3 H) ppm. ^31^P NMR (121 MHz, CDCl_3_): *δ*=15.2 ppm. ^13^C NMR (63 MHz, CDCl_3_): *δ*=234.4 (W*C*O), 227.7 (d, ^2^ *J*_CP_=4.1 Hz, W*C*_syn_), 204.7 (d, *J=*48.0 Hz, W*C*_anti_), 153.3, 152.3, 148.3, 143.6, 142.3, 140.6 (*C*CH_3_), 137.8 (d, *J*_CP_=7.9 Hz, *ipso*‐Ph), 135.8 (*ipso*‐Ph), 135.3, 134.9, 133.6, 133.2, 129.8, 128.7, 128.5, 127.9, 127.5, 127.4 (Ph‐*C*), 113.6, 113.4, 113.2 (*C*CH_3_), 82.7 (*C*OH) 37.5 (d, *J*_CP_=3.5 Hz), 33.8, 25.3, 22.0, 21.6 (*C*H_2_), 19.0 (d, *J*_PC_=17.5 Hz), 17.7, 15.8, 11.5, 11.3, 10.8, 8.7, 8.4, 8.3 (C*C*H_3)_  ppm. IR (DCM): ṽ=2559 (w, BH), 1926 (s, CO) cm^−1^. Elemental analysis calcd: (%) for C_40_H_51_BIN_6_O_2_PW: C 47.49, H 5.01, N 8.52; found: C 47.59, H 5.017, N 8.50.

**3 b**: ^1^H NMR (300 MHz, CD_2_Cl_2_): *δ*=7.5–7.4 (m, 3 H, Ar‐*H*), 7.3–7.2 (m, 1 H, Ar‐*H*), 7.2–7.0 (m, 8 H, Ar‐*H*), 6.8 (t, *J=*7.5 Hz, 2 H), 6.8–6.7 (m, 2 H), 6.5 (t, ^3^ *J_HH_*=8.0 Hz, 5 H, Ar‐*H*), 5.0 (s, 1 H, O*H*), 2.8 (s, 3 H), 2.6 (s, 3 H), 2.4 (d, *J=*2.6 Hz, 3 H), 2.2 (s, 3 H), 2.2 (s, 3 H), 1.8 (s, 3 H), 1.8 (s, 3 H), 1.5 (s, 3 H), 0.8 (s, 3 H, CC*H*_*3)*_  ppm; ^31^P NMR (122 MHz, CD_2_Cl_2_): *δ*=14.9 ppm; ^13^C NMR (63 MHz, CD_2_Cl_2_): *δ*=(no clear signals were detected for W*C*O, W*C*_syn_, W*C*_anti_ as well as most of Ar‐C due to poor solubility) 153.6, 152.7, 150.6, 150.3, 147.4, 144.6, 143.1, 141.6, 139.1 (*C*Me_3_), 129.4, 129.2, 129.1, 128.8, 128.4, 127.8, 127.6 (*C*CH), 114.3, 114.0, 113.9 (*C*CH_3_), 19.9, 19.5, 18.1, 14.4, 11.6, 11.5, 11.0, 8.7, 8.5 (C*C*H_3)_  ppm. IR (DCM): ṽ=2560 (w, BH), 1933 (s, CO) cm^−1^. Elemental analysis calcd: (%) for C_46_H_49_BIN_6_O_2_PW: C 51.61, H 4.61, N 7.85; found: C 51.41, H 4.60, N 7.83.

**3 c**: ^1^H NMR (300 MHz, CD_2_Cl_2_): *δ*=7.96–7.86 (m, 1 H, Ar‐*H*), 7.39–7.28 (m, 2 H, Ar‐*H*), 7.27–7.21 (m, 4 H, Ar‐*H*), 7.19–7.11 (m, 1 H, Ar‐*H*), 7.0–6.9 (m, 2 H, Ar‐*H*), 6.76–6.63 (m, 4 H), 6.41 (t, ^3^ *J*_HH_=8.2 Hz, 2 H, Ar‐*H*), 6.28 (t, ^3^ *J*_HH_=8.2 Hz, 2 H, Ar‐*H*), 5.33 (s, 1 H, O*H*), 2.88 (s, 3 H, CC*H_3_*), 2.50 (s, 3 H, CC*H_3_*), 2.25 (s, 3 H, CC*H_3_*), 2.20 (d, *J_PH_*=2.5 Hz, 3 H, CC*H_3_*), 2.19 (s, 3 H, CC*H_3_*), 1.86–1.83 (m, 9 H, CC*H_3_*), 1.63 (s, 3 H, CC*H*_*3)*_  ppm; ^31^P NMR (122 MHz, CD_2_Cl_2_): *δ*=15.44 ppm; ^13^C NMR (75 MHz, CD_2_Cl_2_): *δ*=237.32 (d, ^3^ *J*_CP_=1.7 Hz, W*C*O), 218.17 (d, ^2^ *J*_CP_=7.3 Hz, W*C*_syn_), 207.60 (d, ^1^ *J*_CP_=50.8 Hz, W*C*_anti_), 153.6, 152.6 (*C*CH_3_), 150.1, 149.7 (*C*CH), 144.5 (*C*CH_3_), 143.7, 143.1 (*C*CH), 141.4, 140.9, 139.7 (*C*CH_3_), 137.4 (d, ^1^ *J*_CP_=7.4 Hz, *C*_ipso_), 136.4 (d, ^1^ *J*_CP_=3.4 Hz, *C*_ipso_), 133.9–120,7 (Ph‐*C*, 18 signals, partially superimposed), 114.1, 114.0, 113.7 (*C*CH_*3*_), 91.8 (*C*OH*)*, 19.4, 19.1, 17.7, 16.9, 11.5, 11.3, 10.7, 8.6, 8.3 (C*C*H_3)_  ppm. IR (DCM): ṽ=2553 (w, BH), 1923 (s, CO) cm^−1^. Elemental analysis calcd: (%) for C_47_H_49_BCl_2_IN_6_O_2_PW: C 48.94, H 4.28, N 7.29; found: C 49.14, H 4.27, N 7.31.

**Preparation of compound 3 d**: A green solution of **3 c** (150 mg, 0.14 mmol) and AgCN (24.4 mg, 0.182 mmol) in ethyl propionate (20 mL) was heated under reflux for 24 h. After cooling, the crude product was filtered through Celite. The volatiles were removed in vacuo and the blue‐green residue was purified by crystallisation. Layering a concentrated solution of the crude product in CH_2_Cl_2_ with *n*‐pentane yielded blue‐green crystals (70 mg, 50 %), which were suitable for XRD analysis.

^1^H NMR (250 MHz, CD_2_Cl_2_): *δ*=7.9–7.8 (m, 1 H, Ar‐*H*), 7.4–7.2 (m, 6 H, Ar‐*H*), 7.2–7.1 (m, 1 H, Ar‐*H*), 7.0 (br. s, 2 H), 6.7 (br. s, 4 H), 6.4 (br. s, 4 H), 4.8 (d, *J=*1.4 Hz, 1 H, O*H*), 2.9 (s, 3 H), 2.5 (s, 3 H), 2.2 (s, 3 H), 2.2 (s, 3 H), 2.1 (s, 3 H), 1.9 (s, 6 H), 1.8 (s, 3 H), 1.7 (s, 3 H, C*H*3) ppm; ^31^P NMR (101 MHz, CD_2_Cl_2_): *δ*=15.0 ppm; ^13^C NMR (63 MHz, CD_2_Cl_2_): *δ*=236.9 (d, ^3^ *J*_CP_=2.3 Hz, W*C*O), 219.9 (d, ^2^ *J*_CP_=8.5 Hz, W*C*_syn_), 213.0 (d, *J*=48.6 Hz, WC_anti_), 152.9 (CCH), 152.3 (d, JCP=1.1 Hz, WCN), 151.9, 149.4, 148.7 (CCH), 144.3, 144.1, 144.0 (CCH3), 142.0, 141.0, 139.8 (CCH3), 133.5–120.8 (Ph‐C, 20 signals, partially superimposed), 114.0, 113.7 (CCH3), 91.3 (COH), 16.3, 15.7, 15.6, 15.4, 11.6, 11.3, 10.9, 8.6, 8.5 (CCH3) ppm. IR (DCM): ṽ=2556 (w, BH), 2099 (w, CN), 1934 (s, CO) cm^−1^. Elemental analysis calcd: (%) for C_47_H_47_BN_7_O_2_PW: C 58.34, H 4.90, N 10.13; found: C 58.25, H 4.92, N 10.09.

**Preparation of compound 4 a**: A green solution of **3 a** (250 mg, 0.253 mmol) in CH_2_Cl_2_ (15 mL) was treated with HO_3_SCF_3_ (44 μL, 0.51 mmol) solved in CH_2_Cl_2_ (3 mL) and stirred at room temperature. The colour changed from green to yellow and after 72 h back to green. The volatiles were removed in vacuo and the green residue was recrystallized by layering a concentrated solution of **4 a** in CH_2_Cl_2_ with *n*‐pentane (112 mg, 45 %). Single crystals suitable for XRD analysis were obtained in this manner.

^1^H NMR (500 MHz, CDCl_3_): *δ*=7.2 (t, *J=*7.4 Hz, 1 H, Ar‐*H*), 7.1 (t, *J=*7.3 Hz, 1 H, Ar‐*H*), 7.1–7.0 (m, 4 H, Ar‐*H*), 6.8 (dt, *J=*8.0 Hz, *J=*27.8 Hz, 4 H, Ar‐*H*), 6.2 (s, 1 H, olefin), 2.8 (s, 3 H, CC*H_3_*), 2.6 (s, 3 H, CC*H_3_*), 2.4 (s, 1 H), 2.4–2.3 (m, 1 H), 2.3–2.3 (m, 1 H), 2.2 (s, 3 H, CC*H_3_*), 2.2 (s, 3 H, CC*H_3_*), 2.1 (d, *J=*1.4 Hz, 3 H, CC*H_3_*), 1.9 (d, *J=*3.6 Hz, 1 H), 1.9 (s, 3 H, CC*H_3_*), 1.8 (s, 3 H, CC*H_3_*), 1.6 (m, 4 H), 1.6 (s, 3 H, CC*H_3_*), 1.4 (s, 3 H, CC*H*_*3)*_  ppm. ^31^P NMR (121 MHz, CD_2_Cl_2_): *δ*=16.9 ppm. ^13^C NMR: without useful information due to poor solubility. IR (CH_2_Cl_2_): ṽ=2557 (w, BH), 1912 (s, CO) cm^−1^. Elemental analysis calcd: (%) for C_39_H_47_BIN_6_OPW⋅C_0.5_HCl: C 48.37, H 4.89, N 8.68; found: C 47.09, H 4.80, N 8.27.

**Preparation of compounds 4 b**‐B(C_6_F_5_)_4_ **and 4 c**‐B(C_6_F_5_)_4_: A green solution of **3 b/3 c** (50 mg, 0.047 mmol) in CH_2_Cl_2_ (15 mL) was treated with [H(Et_2_O)_2_][B(C_6_F_5_)_4_] (39 mg, 0.047 mmol) solved in CH_2_Cl_2_ (3 mL) and stirred at room temperature for 75 min (**4 b^+^**) or overnight (**4 c^+^**). The colour changed from green to red‐brown to yellow. The volatiles were removed in vacuo and the yellow residue was purified by column chromatography on silica using a 1:1 mixture of petroleum ether and CH_2_Cl_2_ (**4 b‐**B(C_6_F_5_)_4_) or via crystallisation by slow diffusion of *n*‐pentane into a concentrated solution of **4 c‐**B(C_6_F_5_)_4_ in CH_2_Cl_2_. The solvents were removed yielding a yellow powder (**4 c‐**B(C_6_F_5_)_4_: 31 mg, 40 %, **4 c‐**B(C_6_F_5_)_4_: 28 mg, 37 %). Single crystals suitable for XRD analysis were obtained by cooling down of a concentrated solution of **4 b**‐B(C_6_F_5_)_4_ in toluene. In case of **4 c^+^** single crystals suitable for XRD analysis were obtained by using HOTf instead of [H(Et_2_O)_2_][B(C_6_F_5_)_4_] by an analogous procedure.

**4 b**‐B(C_6_F_5_)_4_: ^1^H NMR (250 MHz, CD_2_Cl_2_): *δ*=7.9–7.8 (m, 3 H, Ar‐*H*), 7.8–7.7 (m, 3 H, Ar‐*H*), 7.6 (td, *J=*3.8 Hz, *J=*7.9 Hz, 3 H, Ar‐*H*), 7.4–7.3 (m, 8 H, Ar‐*H*), 6.9 (ddd, *J=*1.2 Hz, *J=*8.4 Hz, *J=*13.4 Hz, 2 H, Ar‐*H*), 6.5 (d, *J=*4.7 Hz, 1 H, WCC‐*H*), 2.6 (s, 3 H), 2.6 (s, 3 H), 2.6 (s, 3 H), 2.2 (s, 3 H), 1.7 (s, 3 H), 1.7 (s, 3 H), 1.5 (s, 3 H), 1.4 (s, 3 H), 0.7 (s, 3 H, C*H*_3)_  ppm; ^31^P NMR (101 MHz, CD_2_Cl_2_): *δ*=9.8 ppm; ^19^F NMR (282 MHz, CD_2_Cl_2_): *δ*=−132.5 (m, br, *o*‐F), −163.3 (t, *J*_F‐F_=20.5 Hz, *p*‐F), −166.9 (t, *J*_F‐F_=19.7 Hz, *m*‐F) ppm; ^13^C NMR (75 MHz, CDCl_3_): *δ*=(no clear signals were detected for W*C*O, W*C*_syn_, W*C*_anti_, due to poor solubility), 153.8, 153.2(*C*CH_3_), 149.0, 148.7, 146.7 (*C*CH_3_), 144.4, 143.5 (*C*CH), 142.3, 136.3 (*C*CH_3_), 135.6 (d, ^1^ *J*_CP_=3.1 Hz, *C*_ipso_), 135.5 (d, ^1^ *J*_CP_=2.0 Hz, *C*_ipso_), 134.7, 134.2, 134.2, 133.8, 133.7, 133.3, 133.2, 132.1, 132.0, 130.7, 130.5, 129.9, 129.8, 129.7, 129.6, 128.8 (Ar‐*C*H), 114.9, 114.2, 113.7 (CCH_3_), 113.6 (CC*C*H), 22.5, 17.9, 17.3, 13.7, 11.5, 10.8, 8.4, 8.4, 8.1 (C*C*H_3)_  ppm. IR (DCM): ṽ=2566 (w, BH), 1975 (s, CO) cm^−1^. Elemental analysis calcd: (%) for C_70_H_48_B_2_F_20_IN_6_OPW: 48.47, H 2.91, N 4.85; found: C 48.45, H 2.92, N 4.84.

**4 c**‐B(C_6_F_5_)_4_: ^1^H NMR (300 MHz, CDCl_3_): *δ*=8.6 (d, *J=*7.4 Hz, 1 H, Ar‐*H*), 8.1 (d, *J=*7.5 Hz, 1 H, Ar‐*H*), 8.0–7.9 (m, 1 H, Ar‐*H*), 7.9–7.6 (m, 10 H, Ar‐*H*), 7.3 (d, *J=*8.0 Hz, 1 H, Ar‐*H*), 6.8–6.6 (m, 2 H, Ar‐*H*), 6.2 (d, *J=*4.1 Hz, 1 H, WCC‐*H*), 2.7 (s, 3 H), 2.4 (s, 3 H), 2.4 (s, 1 H), 2.4 (s, 3 H), 2.3 (s, 3 H), 2.2 (s, 3 H), 1.8 (s, 3 H), 1.7 (s, 3 H), 1.5 (s, 3 H), 0.6 (s, 3 H, C*H*3) ppm. ^31^P NMR (122 MHz, CDCl_3_): *δ*=10.9 ppm; ^19^F NMR (471 MHz, CD_2_Cl_2_): *δ*=−133.1 (d, *J=*15.6 Hz), −163.8 (t, *J=*20.5 Hz), −167.6 (t, *J=*19.1 Hz) ppm. ^13^C NMR: without useful information due to poor solubility. **IR** (DCM): ṽ=2565 (w, BH), 1976 (s, CO) cm^−1^. Elemental analysis calcd: (%) for C_70_H_46_B_2_F_20_IN_6_OPW: 48.47, H 2.91, N 4.85; found: C 48.45, H 2.92, N 4.84.

**Preparation of compound 5**: A green solution of **3 c** (132 mg, 0.12 mmol) in CH_2_Cl_2_ (10 mL) was treated with HOTf (0.013 mL, 0.15 mmol) solved in CH_2_Cl_2_ and stirred for 24 h at room temperature. The colour changed from green to yellow. Subsequently, *t*BuOK (17 mg, 0.15 mmol) was added causing the solution to turn deep green. All volatiles were removed in vacuo and the black residue was purified by column chromatography on silica using a 1:6 mixture of petroleum ether and CH_2_Cl_2_. Single crystals suitable for XRD analysis were obtained by slow diffusion of *n*‐pentane into a concentrated solution of **5** in CH_2_Cl_2_.

^1^H NMR (300 MHz, CDCl_3_): *δ*=8.7 (d, *J=*8.0 Hz, 1 H), 8.3 (ddd, *J=*0.9 Hz, *J=*2.0 Hz, *J=*7.2 Hz, 1 H), 8.2 (dt, *J=*1.1 Hz, *J=*7.7 Hz, 1 H), 7.6 (ddd, *J=*1.2 Hz, *J=*7.1 Hz, *J=*8.2 Hz, 1 H), 7.6–7.5 (m, 1 H), 7.4–7.3 (m, 3 H), 7.3 (s, 2 H), 7.2–7.1 (m, 3 H), 7.1 (td, *J=*3.2 Hz, *J=*7.8 Hz, 2 H), 6.8 (ddd, *J=*1.3 Hz, *J=*8.3 Hz, *J=*12.8 Hz, 2 H), 2.9 (s, 3 H), 2.6 (s, 3 H), 2.4 (s, 3 H), 2.2 (s, 3 H), 1.8 (s, 3 H), 1.8 (s, 3 H), 1.5 (s, 3 H), 1.1 (s, 3 H), 0.8 (s, 3 H) ppm; ^31^P NMR (122 MHz, CDCl_3_): *δ*=12.9 ppm; ^13^C NMR (75 MHz, CDCl_3_): *δ*=(no clear signals were detected for W*C*O, W*C*_syn_, W*C*_anti_, due to poor solubility) 153.7, 152.9, 142.2, 141.4, 141.0, 138.3 (*C*CH_3_), 133.6–119.8 (Ph‐*C*, 24 signals, partially superimposed),117.5 (WC*C*), 113.5, 113.4, 112.6 (*C*CH_3_), 17.8, 17.8, 12.8, 11.5, 11.1, 10.9, 8.5, 8.4, 8.2 (*C*H_3)_  ppm. IR (DCM): ṽ=2551 (w, BH), 1906 (s, CO) cm^−1^. Elemental analysis calcd: (%) for C_46_H_45_BIN_6_OPW: C 52.60, H 4.32, N 8.00; found: C 52.60, H 4.30, N 7.94.

## Conflict of interest

The authors declare no conflict of interest.

## Supporting information

As a service to our authors and readers, this journal provides supporting information supplied by the authors. Such materials are peer reviewed and may be re‐organized for online delivery, but are not copy‐edited or typeset. Technical support issues arising from supporting information (other than missing files) should be addressed to the authors.

Supplementary
