# Supplementary material for: Facile Synthesis of a Stable Side‐on Phosphinyne Complex by Redox Driven Intramolecular Cyclisation
Source: Chemistry. 2020 Aug 7;26(50):11492–502. doi: 10.1002/chem.201905750 (PMC7540294; doi:10.1002/chem.201905750)
Supplement: Supplementary file 1 — Supplementary [file CHEM-26-11492-s001.pdf]

Chemistry–A European Journal

Supporting Information

Facile Synthesis of a Stable Side-on Phosphinyne Complex by Redox Driven Intramolecular Cyclisation

Helge Lange,[a] Henning Schröder,[b] Elisabeth Oberem,[a] Alexander Villinger,[a] Jabor Rabeah,[c] Ralf Ludwig,[a, c] Klaus Neymeyr,[b, c] and Wolfram W. Seidel*[a]

Content

1. EPR measurement of intermediate **(IM1)$_2$$^{2+}$** 2
2. Cyclic voltammetry and spectro-electrochemical measurements 2
3. Crystallographic Details 4
4. Multicomponent mixture spectra analysis with multivariate curve resolution 10
   4.1. Reaction of **3c** with $[H(Et_2O)_2][B(C_6F_5)_4]$ 10
   4.2. Reaction of **3d** with $[H(Et_2O)_2][B(C_6F_5)_4]$ 12
5. Reaction monitoring with ^{31}P NMR spectroscopy 13
   5.1. Reaction of **3b** with $[H(Et2O)_2][B(C_6F_5)_4]$ monitored by ^{31}P NMR 13
   5.2. Reaction of **3c** with $[H(Et2O)_2][B(C_6F_5)_4]$ monitored by ^{31}P NMR 14
6. NMR-Spectra 16
   6.1. NMR-spectra of compound **3a** 16
   6.2. NMR-spectra of compound **3b** 18
   6.3. NMR-spectra of compound **3c** 20
   6.4. NMR-spectra of compound **3d** 22
   6.5. NMR-spectra of compound **4b**-$[(B(C_6F_5)_4]$ 23
   6.6. NMR-spectra of compound **4c**-OTf 27
   6.7. NMR-spectra of compound **5** 29
7. DFT calculation details 31
8. References 49

1. EPR measurement of intermediate $(\mathbf{IM1})_2^{2+}$

The X-band EPR spectrum was recorded on a Bruker EMX CW-micro spectrometer equipped with an ER 4131VT Digital Temperature Control System and an ER 4119HS-WI high-sensitivity optical resonator. The calibration of the *g* value was performed using DPPH (2,2diphenyl-1-picrylhydrazyl) (g = 2.0036±0.00004). A sample of the reaction mixture of **3c** with Jutzi acid in CH_2Cl_2 was filled into a J. Young EPR tube under inert conditions and measured 90 minutes after the start of the reaction at 300 K. Analysis of the hyper fine coupling was performed using the simulation program EPRsim32.[S1]

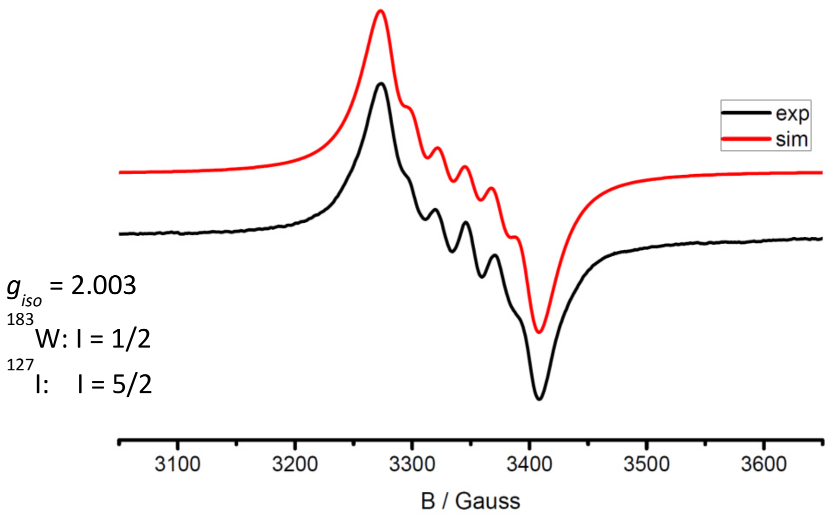

Figure S1. X-band EPR spectrum of reaction mixture **3c** with $[H(Et_2O)_2][(B(C_6F_5)_4]$ at 300 K after 90 min; black = experiment, red = simulation with g_{iso} = 2.003; hyperfine coupling to ^{127}I(S = 5/2, 100%) with A_{iso} = 25 10^{-4} cm^{-1}. Hyperfine coupling to ^{183}W was not accounted for because of restricted resolution.

2. Cyclic voltammetry and spectro-electrochemical measurements

Cyclic voltammetry was performed using a Princeton Applied Research VersaSTAT 3. A three electrode arrangement with a glassy carbon working electrode, a platinum wire counter electrode and an Ag/AgCl in CH_3CN reference electrode and 0.15 M $^{n}Bu_4NPF_6$ as supporting electrolyte was employed. The ferrocene/ferrocenium (Fc/Fc^+) redox couple was used as internal standard.

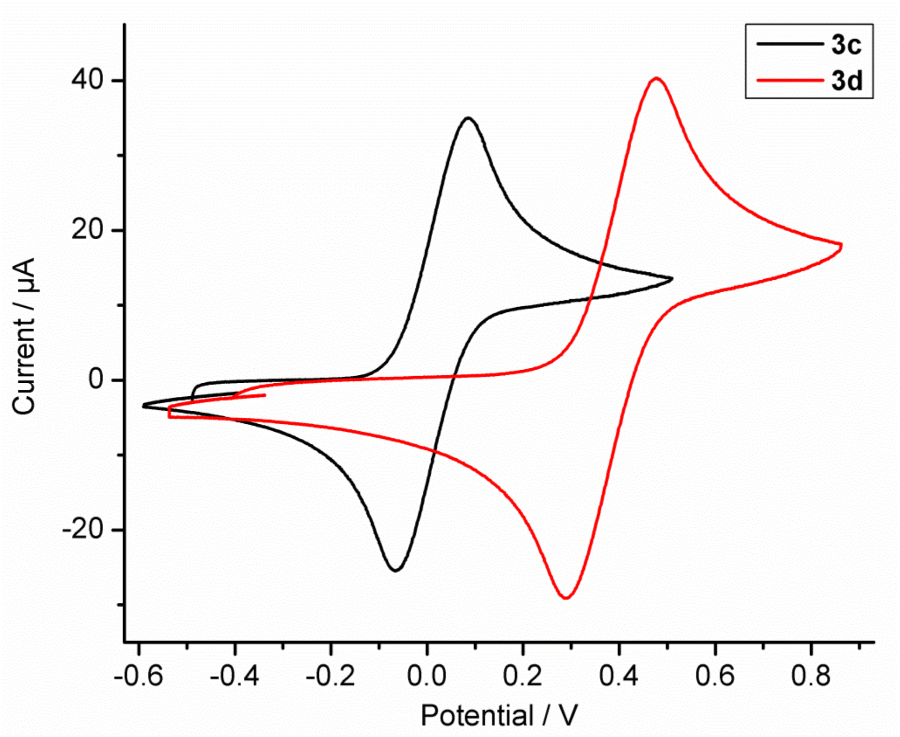

Figure S2. Cyclic voltammetry of complex **3c** (black) and **3d** (red) measured in CH_2Cl_2 (referenced against ferrocene/ferrocenium). **3c** shows a reversible oxidation at $E_{1/2}$ = +0.011 V. **3d** shows a reversible oxidation at $E_{1/2}$ = +0.38 V. Both are putative $W^{II/III}$ redox process. [S2]

Spectro-electrochemical data were also acquired with a Princeton Applied Research VersaSTAT 3 potentiostat. The optically transparent thin-layer electrochemical (OTTLE) cell was home-built. It comprised a Pt working and counter electrode and a thin silver wire as a pseudoreference electrode sandwiched between two CaF_2 windows of a conventional liquid IR cell. The working electrode was positioned in the centre of the spectrometer beam. The build-up followed the design of Hartl and co-workers and modifications of Winter *et al.*[S3]

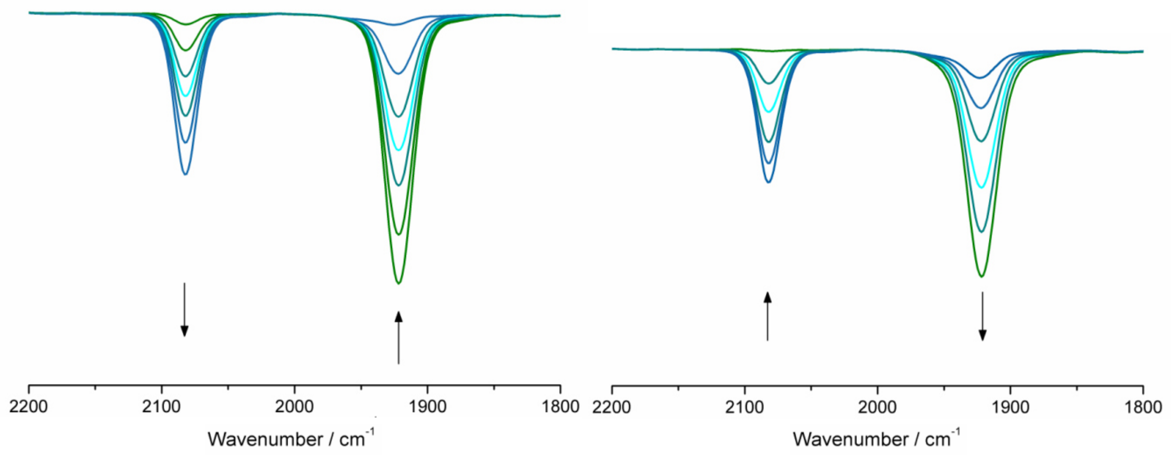

Figure S3. IR spectro-electrochemical measurement of **3c**. Changes of the spectra during oxidation (left) and reduction (right) shows reversible W(II)/W(III) oxidations steps. The CO vibration increases from 1923 cm^{-1} to 2085 cm^{-1}

3. Crystallographic Details

Single crystals suitable for X-ray diffraction analysis were selected in Fomblin YR-1800 perfluoropolyether oil (Alfa Aesar) at ambient temperature and mounted on a glass fibre. During the measurement the samples were cooled to 173(2) K. Diffraction data were collected on a Bruker-Nonius Apex X8, Bruker D8 Quest- diffractometer and a Bruker Kappa Apex II diffractometer using graphite monochromated Mo-K· radiation (λ = 0.71073 Å). Structure solutions were found by direct methods (SHELXS-97)[S4] and were refined by full-matrix least-squares procedures on F_2 (SHELXL-97).[S5] All non-hydrogen atoms were refined anisotropically, and hydrogen atoms were included at calculated positions with fixed thermal parameters.

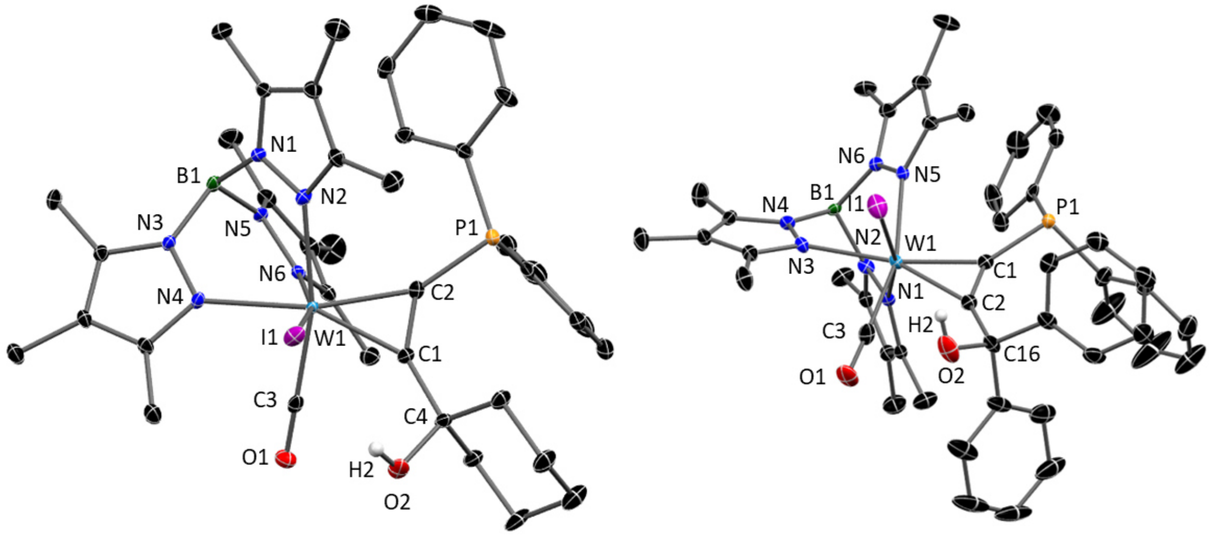

Figure S4. Molecular crystal structure of the complex **3a** (left) and **3b** (right) (50% thermal ellipsoids). Hydrogen atoms have been omitted for clarity. Selected bond lengths (Å) and angles (°): **3a**: P1-C2 1.811(3), C1-C2 1.314(5), C1-C4 1.516(4), W1-C1 2.043(3), W1-C2 2.063(3), W1-C3 1.971(3), C2-C1-C4 141.2(3), C1-C2-P1 134.0(3). **3b** W1-C1 2.063(4), W1-C2 2.067(4) P1-C1 1.795(4), C1-C2 1.316(5), C2-C16 1.529(5), O2-C16 1.452(5), C2-C1-P1 141.3(3), C1-C2-C16 142.2(4).

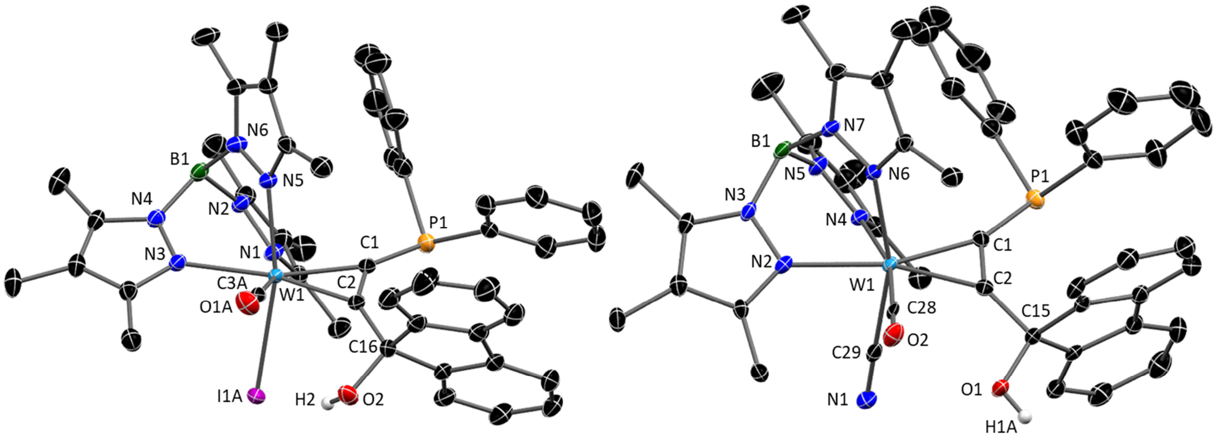

Figure S5. Molecular crystal structure of the complex **3c** (left) and **3d** (right) (50% thermal ellipsoids). Hydrogen atoms have been omitted for clarity. Selected bond lengths (Å) and angles (°): **3c**: P1-C1 1.785(3), C1-C2 1.309(4), C2-C16 1.515(4), W1-C2 2.055(3) W1-C1 2.059(2), P1-C16 3.866, W1-C3A 1.972(3), W1-C1 2.055(3), W1-C2 2.059(2), C2-C1-P1 141.0(2), C1-C2-C16 140.3(2). **3d**: P1-C1 1.805(3), C1-C2 1.322(4), C2-C15 1.510(4), W1-C1 2.046(3), W1-C2 2.048(3), W1-C28 1.975(3), W1-C29 2.132(3) C2-C1-P1 136.0(2), C1-C2-C15 139.0(3).

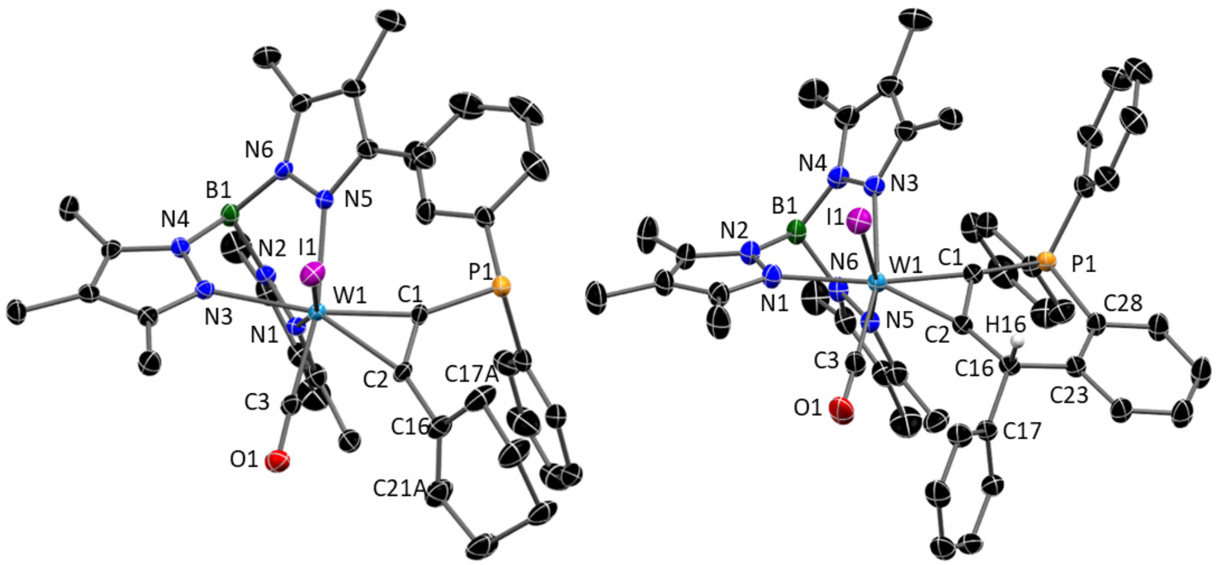

Figure S6. Molecular crystal structure of the complex **4a** (left) and **4b-(B(C_6F_5)$_4$** (right) (50% thermal ellipsoids). Hydrogen atoms and the anion (B(C_6F_5)$_4^-$ of **4b**$^+$ have been omitted for clarity. Selected bond lengths (Å) and angles (°): **4a**: P1 C1 1.795(4), C1 C2 1.322(6), C2 C16 1.468(5), C16-C21A 1.336(17), C16-C17A 1.510(15), C2-C1-P1 129.7(3), C1-C2-C16 136.5(4). **4b**$^+$**:** P1-C1 1.7440(18), P1-C28 1.7864(18), C23-C28 1.405(2), C16-C23 1.537(2), C2-C16 1.502(2), C1-C2 1.332(2), C2-C16-C17 117.21(14), C2-C16-C23 112.34(14), C17-C16-C23 114.06(14).

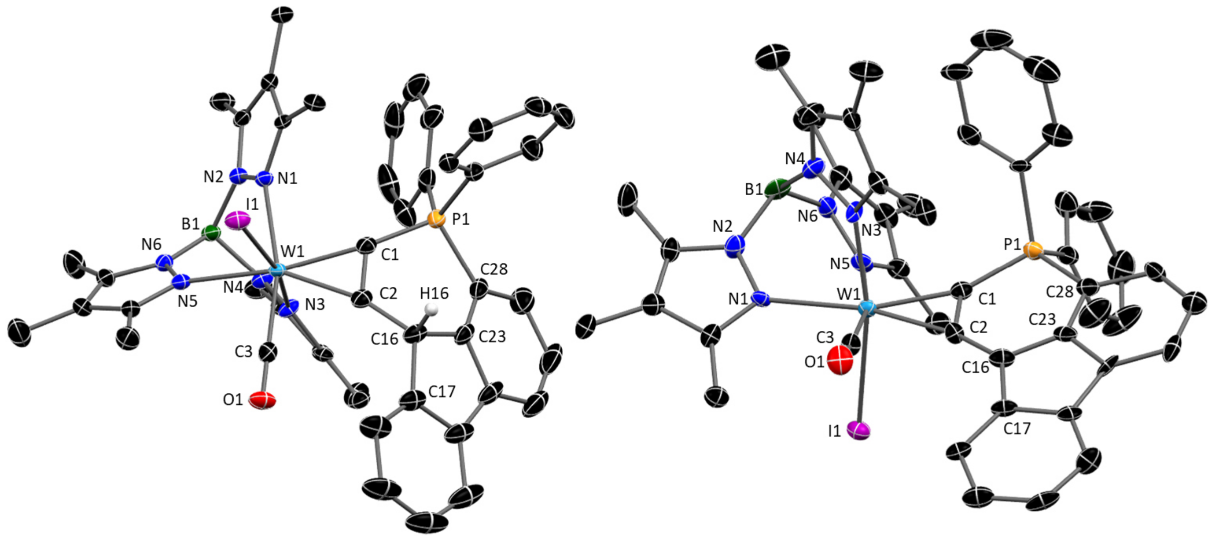

Figure S7. Molecular crystal structure of the complex **4c-OTf** (left) and **5** (right) (50% thermal ellipsoids). Hydrogen atoms and the anion $CF_3SO_3^-$ of **4c$^+$** have been omitted for clarity. Selected bond lengths (Å) and angles (°): **4c$^+$**: P1-C1 1.753(4), P1-C28 1.808(4), C23-C28 1.386(7), C16-C23 1.517(7), C2-C16 1.501(6), C1-C2 1.341(6), C2-C16-C17 124.8(4), C2-C16-C23 107.0(3), C23-C16-C17 103.4(4). **5**: C1-P1 1.743(10), P1-C28 1.772(10), C28-C23 1.395(15), C23-C16 1.426(15), C16-C2 1.401(13), C2-C1 1.383(13), C2-C16-C17 135.4(10), C2-C16-C23 116.3(9), C23-C16-C17 108.1(9).

Table S1. Crystallographic details for **3a**, **3b** and **3c**.

compound	**3a**	**3b**	**3c**
empirical formula	$C_{39}H_{49}BIN_6O_2PW$	$C_{46}H_{49}BIN_6O_2PW$	$C_{46}H_{47}BIN_6O_2PW$, CH_2Cl_2
fw (g mol^{-1})	986.37	1070.44	1153.35
cryst. syst.	monoclinic	monoclinic	monoclinic
space group	P 21/c	P 21/n	P 21/n
a (Å)	10.2536(4)	13.1608(5)	12.2877(11)
b (Å)	17.1296(6)	21.0700(8)	22.488(2)
c (Å)	22.4554(8)	15.4408(6)	16.4961(15)
α (deg)	90	90	90
β (deg)	95.807(2)	96.349(2)	95.703(4)
γ (deg)	90	90	90
V(Å^3)	3923.8(2)	4255.4(3)	4535.8(7)
Z	4	4	4
T (K)	123(2)	123(2)	123(2
ρ (g cm^{-3})	1.670	1.671	1.689
μ (mm^{-1})	3.813	3.524	3.427
measured reflns	122203	136838	85175
indep reflns	14207	12426	16405
reflns with $I>2\sigma(I)$	11122	9085	12434
R_{int}	0.0862	0.1393	0.0674
R1 (F[$I>2\sigma(I)$])	0.0433	0.0449	0.0348
w*R*2 (F^2[all data])	0.0759	0.0673	0.0656
GOF	1.074	1.040	1.030
params	473	533	595
CCDC no.	1953264	1953265	1953270

Table S2. Crystallographic details for **3d**, **4a** and **4b**-$BC_{24}F_{20}$.

compound	**3d**	**4a**	**4b**-$BC_{24}F_{20}$
empirical formula	$C_{47}H_{47}BN_7O_2PW$, 1.895(CH_2Cl_2)	$C_{39}H_{47}BIN_6OPW$	$C_{46}H_{48}BIN_6OPW^+$, $C_{24}BF_{20}^-$, 2.5(C_7H_8)
fw (g mol^{-1})	1128.30	968.35	1962.81
cryst. syst.	triclinic	monoclinic	triclinic
space group	P -1	P 21/c	P -1
a (Å)	11.744(3)	10.3946(2)	14.8637(15)
b (Å)	14.411(3)	17.0819(5)	16.0601(16)
c (Å)	15.520(4)	22.0539(6)	18.2456(18)
α (deg)	92.018(5)	90	72.666(4)
β (deg)	100.353(6)	96.4260(10)	77.615(4)
γ (deg)	108.841(5)	90	86.467(5)
V(Å^3)	2433.2(9)	3891.28(17)	4060.9(7)
Z	2	4	2
T (K)	123(2)	123(2)	123(2)
ρ (g cm^{-3})	1.540	1.653	1.605
μ (mm^{-1})	2.661	3.842	1.920
measured reflns	14872	123568	34575
indep reflns	14872	12254	29362
reflns with $I>2\sigma(I)$	11405	9076	25511
R_{int}	0.0415	0.1076	0.0524
R1 (F[$I>2\sigma(I)$])	0.0378	0.0398	0.0288
wR2 (F^2[all data])	0.0812	0.0703	0.0722
GOF	0.978	1.081	1.018
params	610	506	1124
CCDC no.	1953266	1953267	1953268

Table S3. Crystallographic details for **4c-**CF_3O_3S and **5**.

compound	**4c-**CF_3O_3S	**5**
empirical formula	$C_{46}H_{46}BIN_6OPW^+$, $CF_3O_3S^-$, 2(CH_2Cl_2)	$C_{46}H_{45}BIN_6OPW$
fw (g mol^{-1})	1370.34	1050.41
cryst. syst.	triclinic	orthorhombic
space group	P -1	P 21 21 21
a (Å)	13.5287(12)	11.2493(9)
b (Å)	15.6907(12)	19.2031(15)
c (Å)	15.7435(15)	19.9323(14)
α (deg)	96.623(3)	90
β (deg)	113.751(3)	90
γ (deg)	113.833(2)	90
V(Å^3)	2639.2(4)	4305.8(6)
Z	2	4
T (K)	123(2)	123(2)
ρ (g cm^{-3})	1.724	1.620
μ (mm^{-1})	3.106	3.479
measured reflns	81351	6844
indep reflns	11037	6844
reflns with I>2σ(I)	9145	5536
R_{int}	0.0833	0.0564
R1 (F[I>2σ(I)])	0.0340	0.0505
wR2 (F^2[all data])	0.0680	0.1156
GOF	1.053	1.027
params	777	525
CCDC no.	1953271	1953269

4. Multicomponent mixture spectra analysis with multivariate curve resolution

4.1. Reaction of **3c** with $[H(Et_2O)_2][B(C_6F_5)_4]$

For the reaction monitoring via IR spectroscopy complex **3c** (83.3 mg, 78 µmol) was solved in CH_2Cl_2 (20 mL) and tempered to 20 °C. The stirred reaction mixture was treated with $[H(Et_2O)_2][B(C_6F_5)_4]$ (65 mg, 78 µmol) solved in CH_2Cl_2 (1.5 mL). The reaction mixture was pumped continuously through the IR cell during the addition. A measurement was taken every 30 seconds, with one measurement being the mean of 8 scans. These results in a series of spectra on a $(time \times wavenumber)$ grid from which information about concentration profiles and pure component spectra can be obtained by multivariate curve resolution methods.

For the chemometric analysis of this multicomponent spectral data a kinetic hard-model approach was applied.[S6] A large number of possible reaction mechanisms were analysed (Scheme S2). The reaction orders are optimized within the interval [1,2] if no specifications are given by the assumptions about the reaction system. This can cause broken orders. The results of all reaction mechanisms in Scheme S2 were compared with respect to the reconstruction error of the spectral data and the error of the kinetic model fit. Among all tested reaction mechanisms the one in Scheme S1 leads to optimal results.

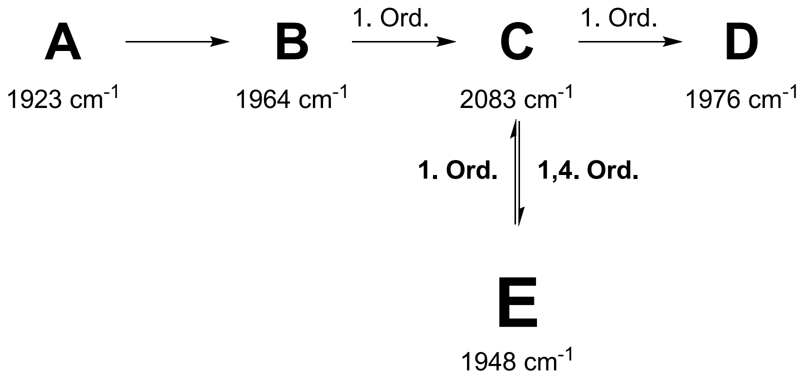

Scheme S1. A reaction mechanism for which optimal reaction rates in terms of small reconstruction and model fit errors can be determined.

A	B	C	D	E	X
1923 cm^{-1}	1964 cm^{-1}	2083 cm^{-1}	1976 cm^{-1}	1948 cm^{-1}	Intermediat in very low concentration

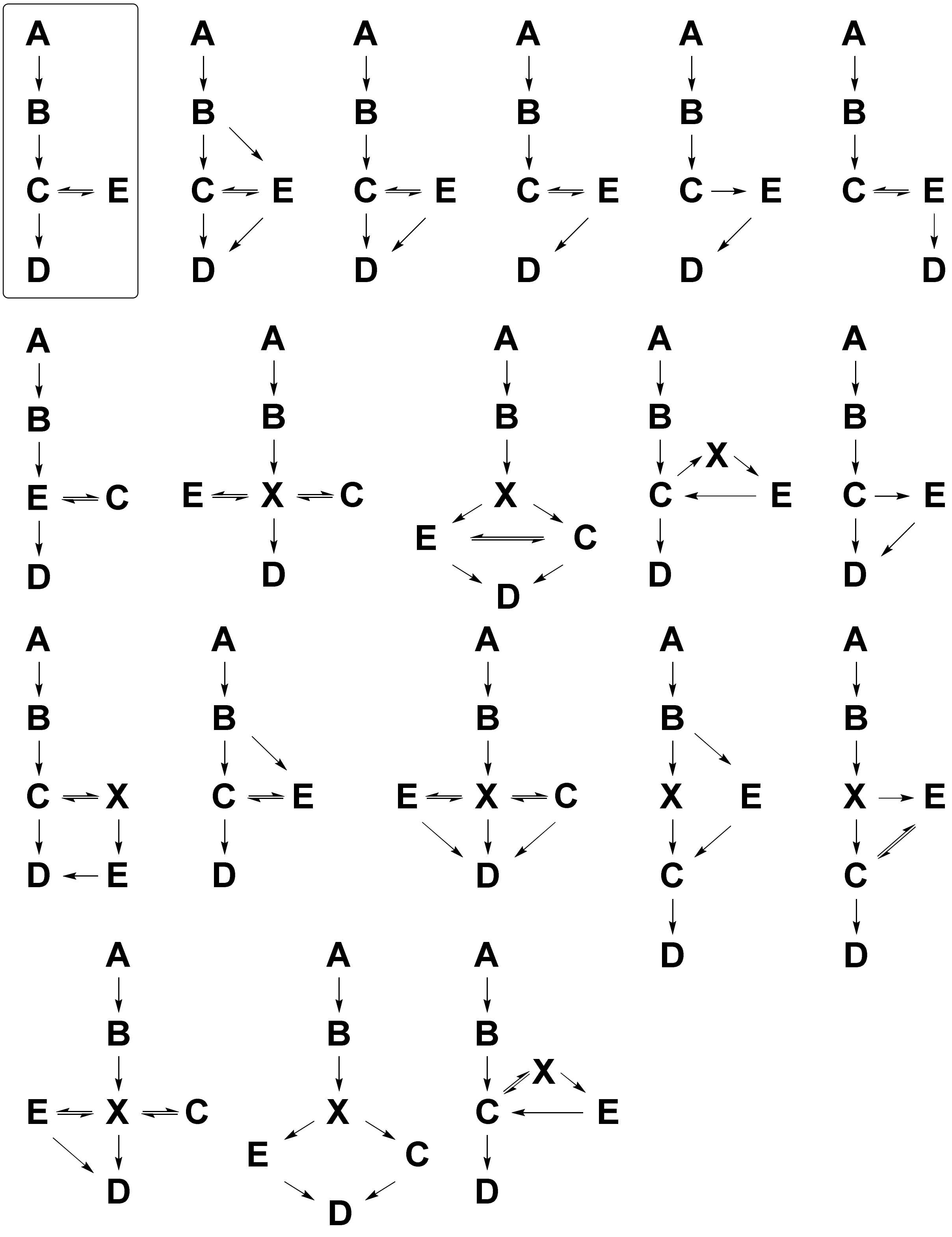

Scheme S2. Analysed reaction mechanism for the reaction of **3c** with Jutzi acid. The model with best fit is marked. The integration of a species **X**, which due to its low concentration does not appear as an

independent band in the IR spectrum, does not lead to a reduction of errors in the kinetic hard-model approach.

4.2. Reaction of **3d** with $[H(Et_2O)_2][B(C_6F_5)_4]$

For the reaction monitoring via IR spectroscopy complex **3d** (30 mg, 31 µmol) was solved in CH_2Cl_2 (17 mL) and tempered to 20 °C. The stirred reaction mixture was treated with $[H(Et_2O)_2][B(C_6F_5)_4]$ (26 mg, 31 µmol) solute in CH_2Cl_2 (1.5 mL). The reaction mixture was pumped continuously through the IR cell during the addition. A measurement was taken every 30 seconds, with one measurement being the mean of 8 scans. Again this results in a series of spectra on a $(time \times wavenumber)$ grid.

The series of spectra was split into two separate date sets. One covers the range for CO vibration around 1950 cm^{-1} and the other the range for the less sensitive CN vibration above above 2100 cm^{-1}. Both data sets were analysed with the peak group analysis.[S7] Pure component spectra and concentration profile have been extracted, see Figure S8. Every CN band can be assigned to a CO band, so that the formation of a W^{III}-species at around 2083 cm^{-1} can be excluded.. It is important to remark that without further assumptions only qualitative but no quantitative concentration-time curves can be obtained by the peak group analysis. Nevertheless, an approximate trend can be extracted. This is shown in particular by the profile for 1933 cm^{-1} (red) and 1992 cm^{-1} (blue) which must be zero from a chemical point of view.

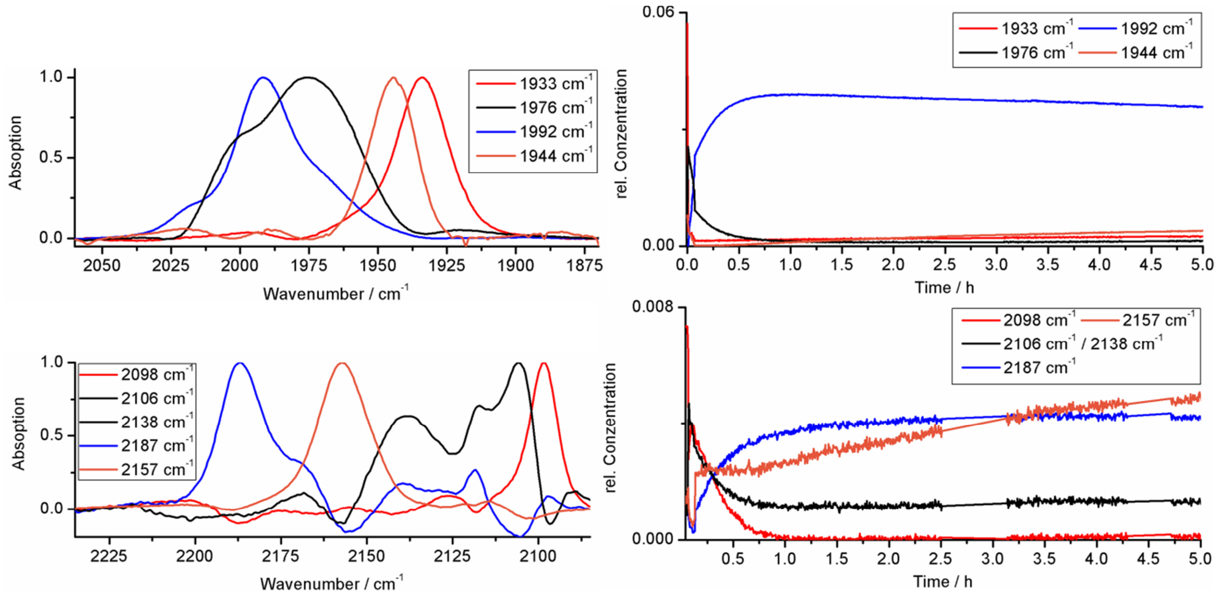

Figure S8. Decompositions of the mixture spectra of the reaction of **3d** with Jutzi acid into the pure component spectra (left) and the corresponding concentration profiles (right). At the top the area around 1950 cm^{-1} is shown, while at the bottom the area over 2100 cm^{-1} is presented.

The unusually broad band at 1976 cm^{-1} and the corresponding bands at 2106 cm^{-1}/2138 cm^{-1}, which formed immediately after the addition of the acid, seem conspicuous. The reason for this is the cyanide ligand, which besides the phosphine can also bind the proton, which leads to two isomers.[S8] The analysis clearly confirms that there was no complete oxidation of the tungsten centre. Instead the positive charge is stabilized by mesomerism between the ligand and the metal. This enables a significantly durable

intermediate with bands at 1992 cm^{-1} and 2187 cm^{-1}. The product has a CO vibration at 1944 cm^{-1} and forms slowly over a period of several days.

It is possible to apply another multivariate curve resolution method, namely a kinetic hard-modelling approach from Section 4.1 for the area around 1950 cm^{-1}. The model in Scheme S3 is used. In comparison to the reaction of **3c** with Jutzi acid it does not contain a dead-end but an equilibrium between the long-lived intermediate $\mathbf{C}_{CN}$ and the product $\mathbf{D}_{CN}$.

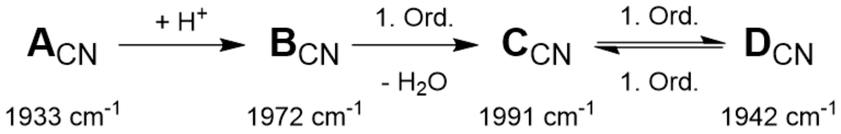

Scheme S3. Derived kinetic model for the reaction sequence of **3d** with Jutzi Acid.

5. Reaction monitoring with ^{31}P NMR spectroscopy

5.1. Reaction mixture after protonation of **3b** with $[H(Et_2O)_2][(B(C_6F_5)_4]$ monitored by ^{31}P NMR spectroscopy

A green solution of **3b** (10 mg, 9.4 µmol) in CD_2Cl_2 (0.5 mL) in a J. Young NMR tube was treated with $[H(Et_2O)_2][B(C_6F_5)_4]$ (7.8 mg, 9.4 µmol) solute in CD_2Cl_2 (0.2 mL) at -80°C. The upper part of the solution colour changed slowly from green to red. The sample was recorded with 128 Scans at different temperature starting at -80 °C using Bruker Avance 500 MHz (Figure S9). The temperature was increased by 20 K after each measurement. At -40 °C a Proton coupled ^{31}P NMR-spectrum was measured and is shown in Figure S10.

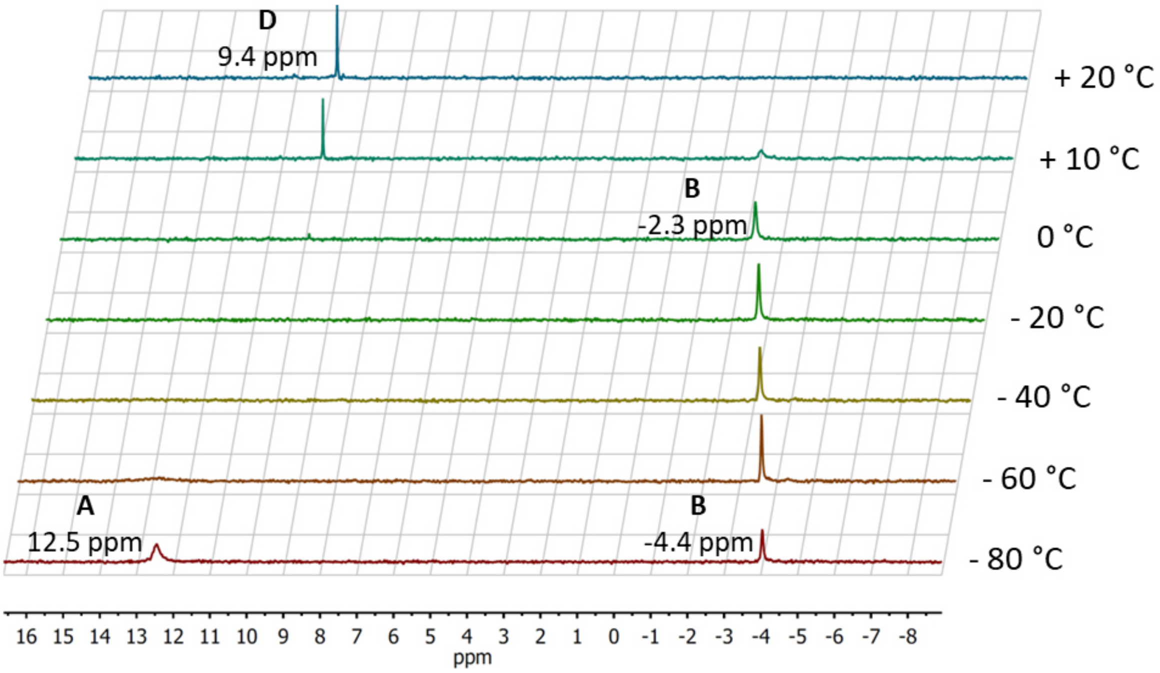

Figure S9. Reaction sequence of **3b** with $[H(Et_2O)_2][(B(C_6F_5)_4]$ in ^{31}P NMR spectroscopy. Reaction mixture was heated stepwise from -80 °C to room temperature.

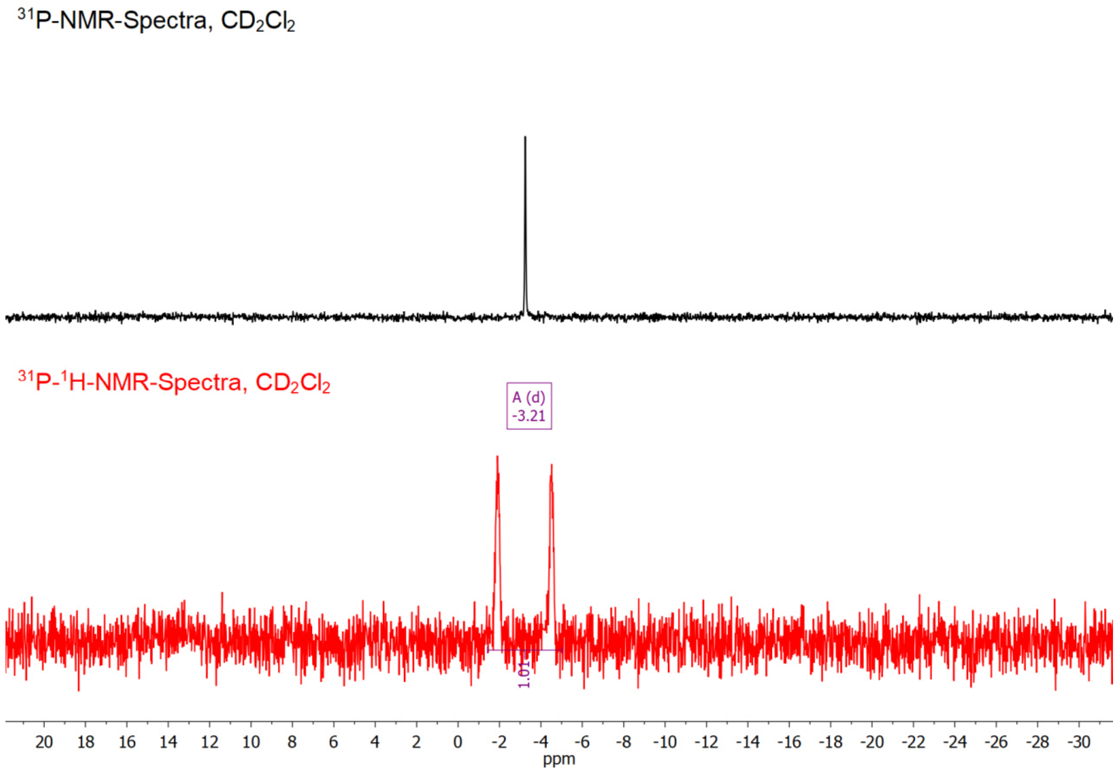

Figure S10. ^{31}P NMR-spectra (top) and ^{31}P-^{1}H-NMR-spectra (bottom) of the reaction **3b** with Jutzi acid at -40 °C. ^{31}P NMR (202 MHz, CD_2Cl_2): δ = -3.2 (d, $^{1}J_{PH}$ = 526.0 Hz) ppm.

5.2. Reaction mixture after protonation of **3c** with $[H(Et_2O)_2][(B(C_6F_5)_4]$ monitored by ^{31}P NMR spectroscopy

A green solution of **3c** (10 mg, 9.4 µmol) in CD_2Cl_2 (0.5 mL) in a J. Young NMR tube was treated with $[H(Et_2O)_2][B(C_6F_5)_4]$ (7.8 mg, 9.4 µmol) solute in CD_2Cl_2 (0.2 mL) at -80°C. The upper part of the solution colour changed slowly from green to red. The sample was recorded with 128 Scans at different temperature starting at -80 °C using Bruker Avance 500 MHz (Figure S11). The temperature was increased by 20 K after each measurement. At -80 °C a Proton coupled ^{31}P NMR-spectrum was measured and is shown in Figure S12.

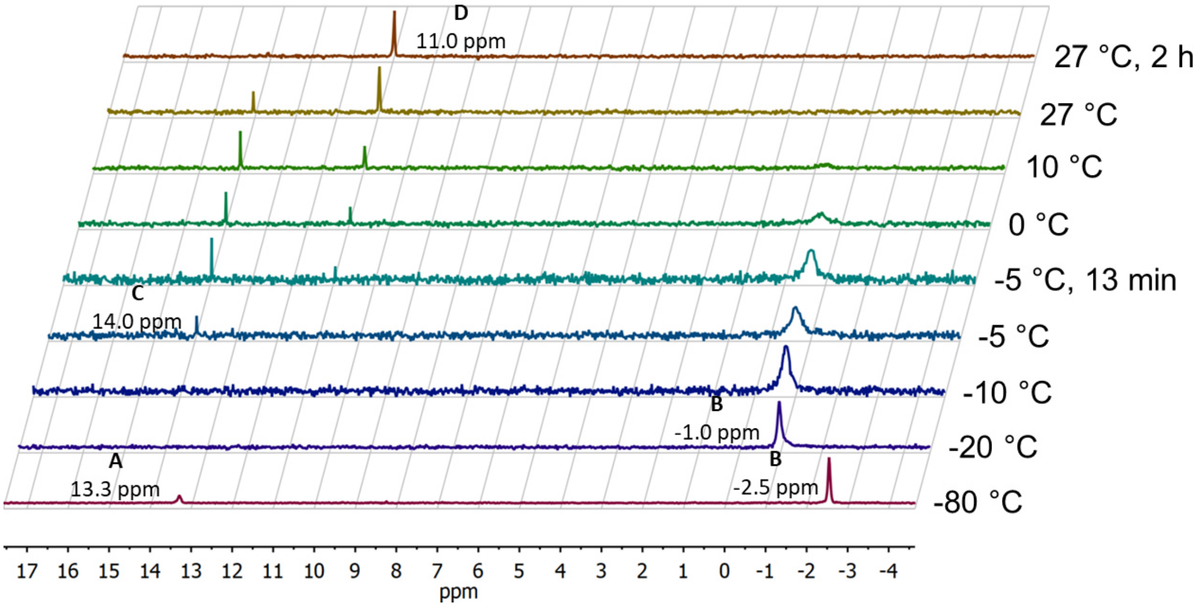

Figure S11. Reaction sequence of **3c** with $[H(Et_2O)_2][(B(C_6F_5)_4]$ in ^{31}P NMR spectroscopy. Reaction mixture was heated stepwise from -80 °C to room temperature. Note the temperature shift of species **B** from -2.5 ppm at -80 °C to -1.0 ppm at -20 °C.

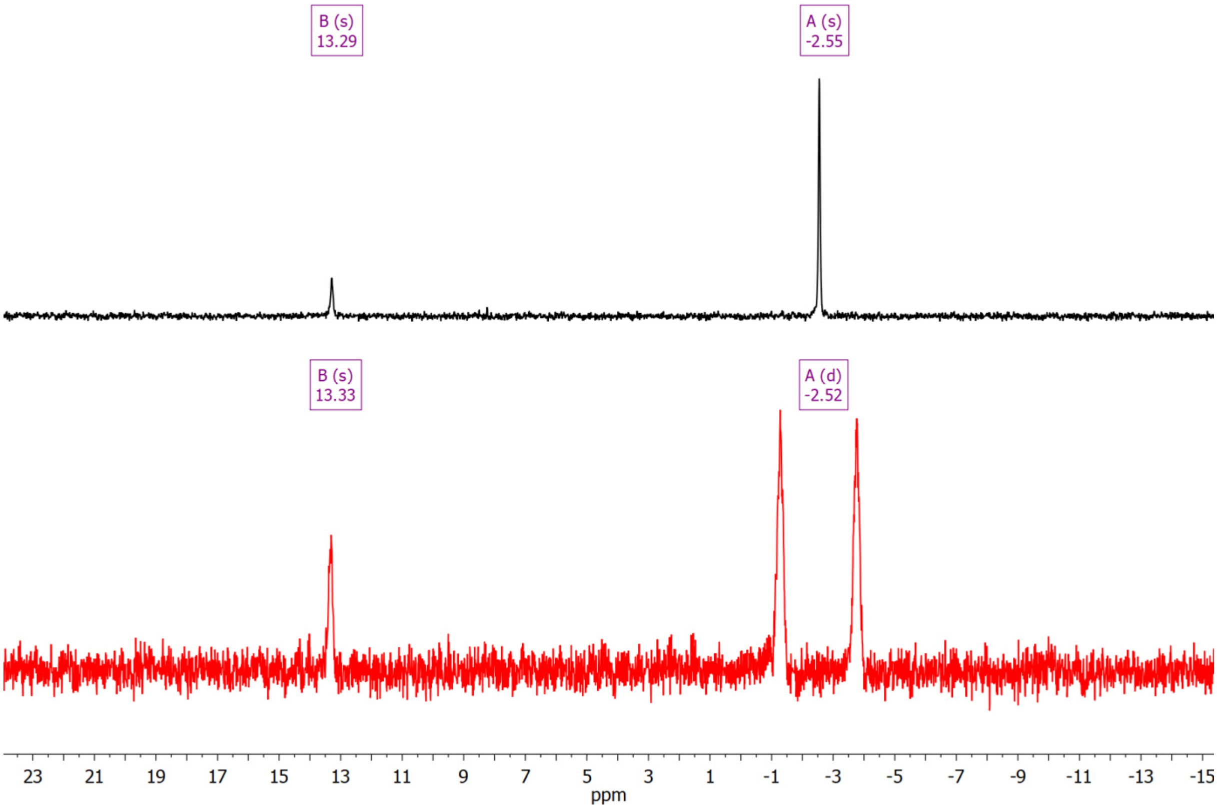

Figure S12. ^{31}P NMR-spectra (top) and ^{31}P-^{1}H-NMR-spectra (bottom) of the reaction **3c** with Jutzi acid at -80 °C. ^{31}P NMR (202 MHz, CD_2Cl_2): δ = -2.5 (d, $^{1}J_{PH}$ = 501.3 Hz) ppm.

6. NMR-Spectra

6.1. NMR-spectra of compound **3a**

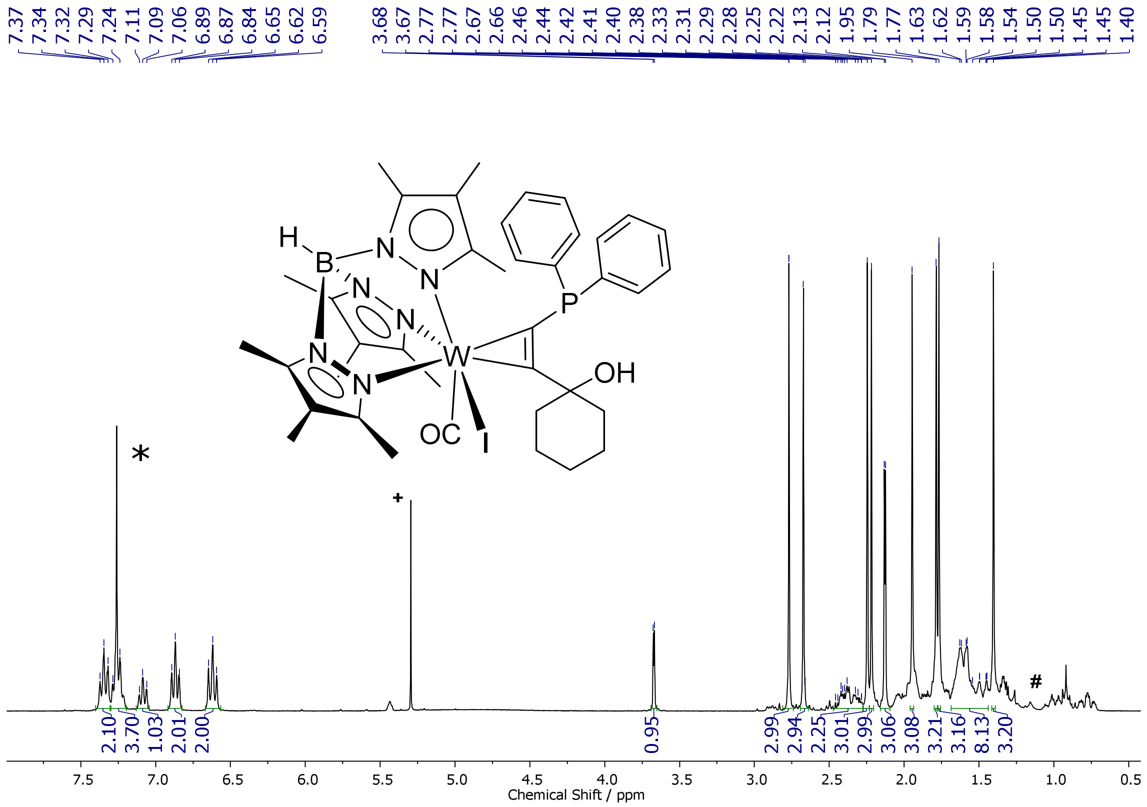

Figure S13. ^{1}H NMR of **3a** in $CDCl_3$ (*); + CH_2Cl_2 # Hexane/Pentane

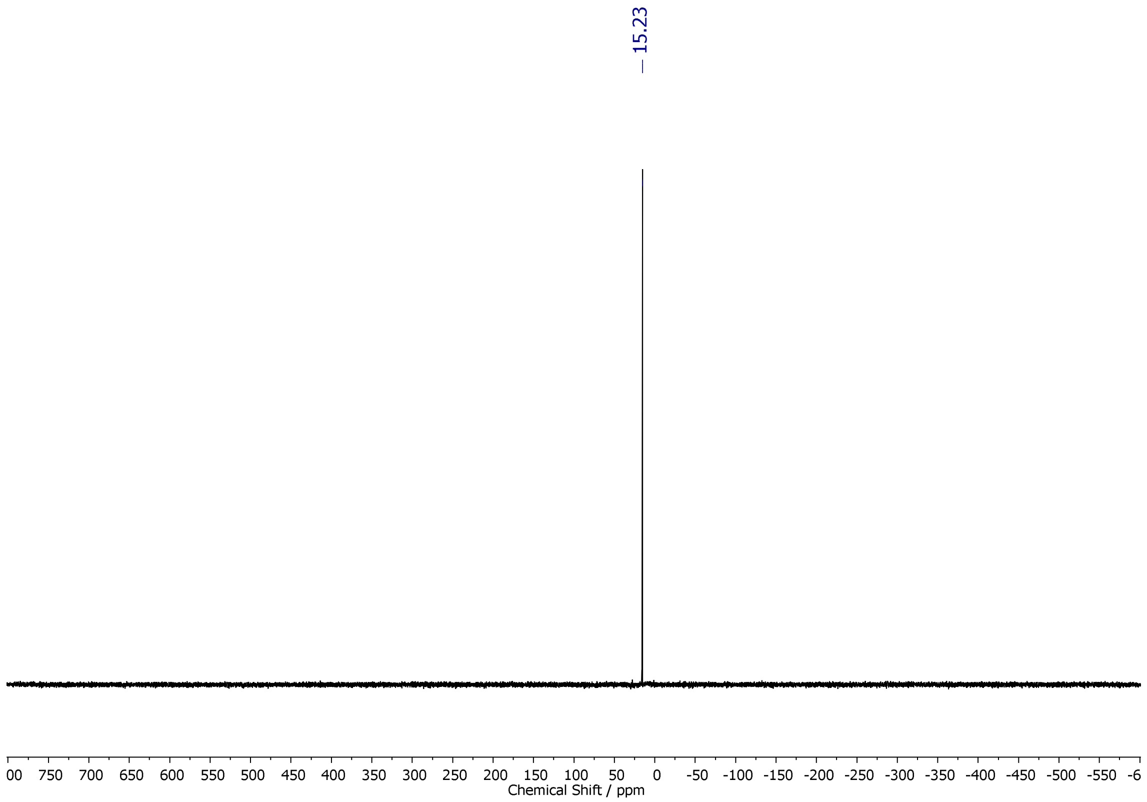

Figure S14. ^{31}P NMR of **3a** in $CDCl_3$

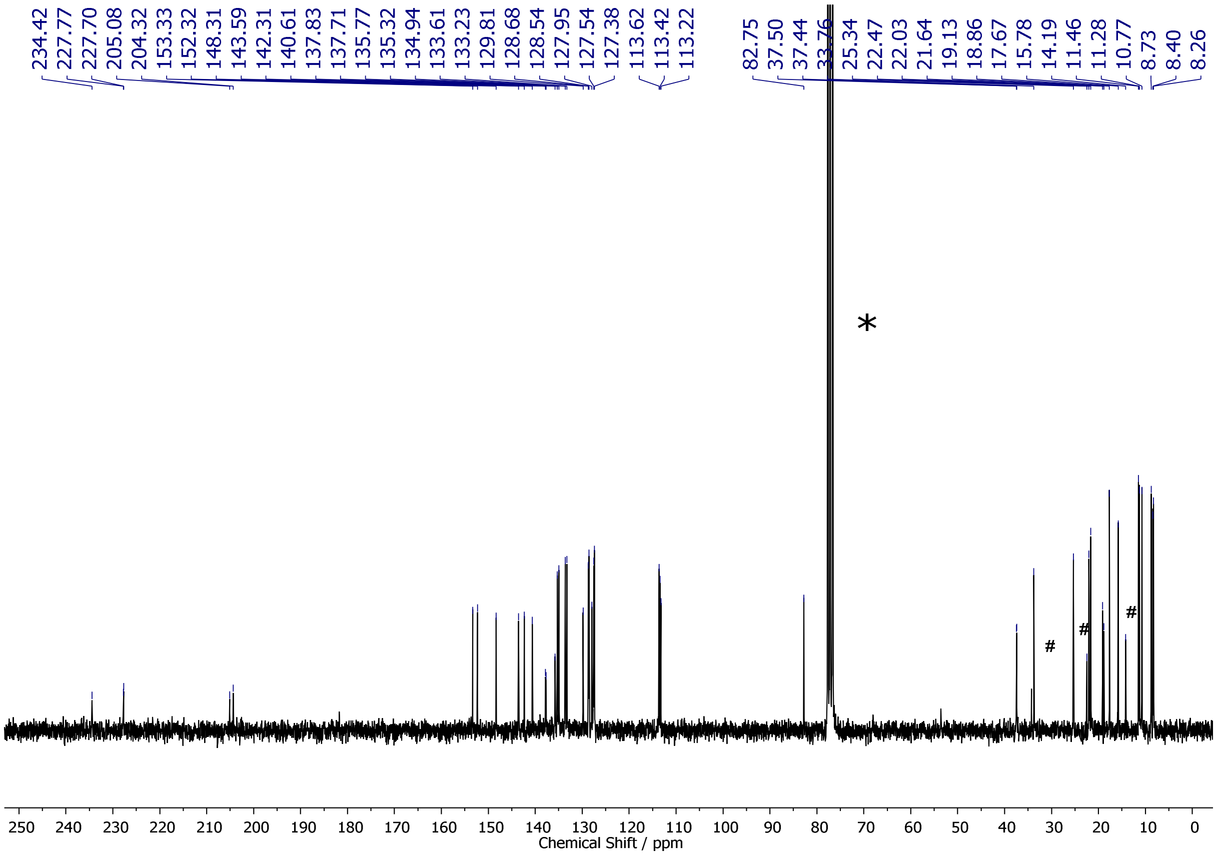

Figure S15. ^{13}C NMR of **3a** in $CDCl_3$ (∗) # Hexane/Pentane

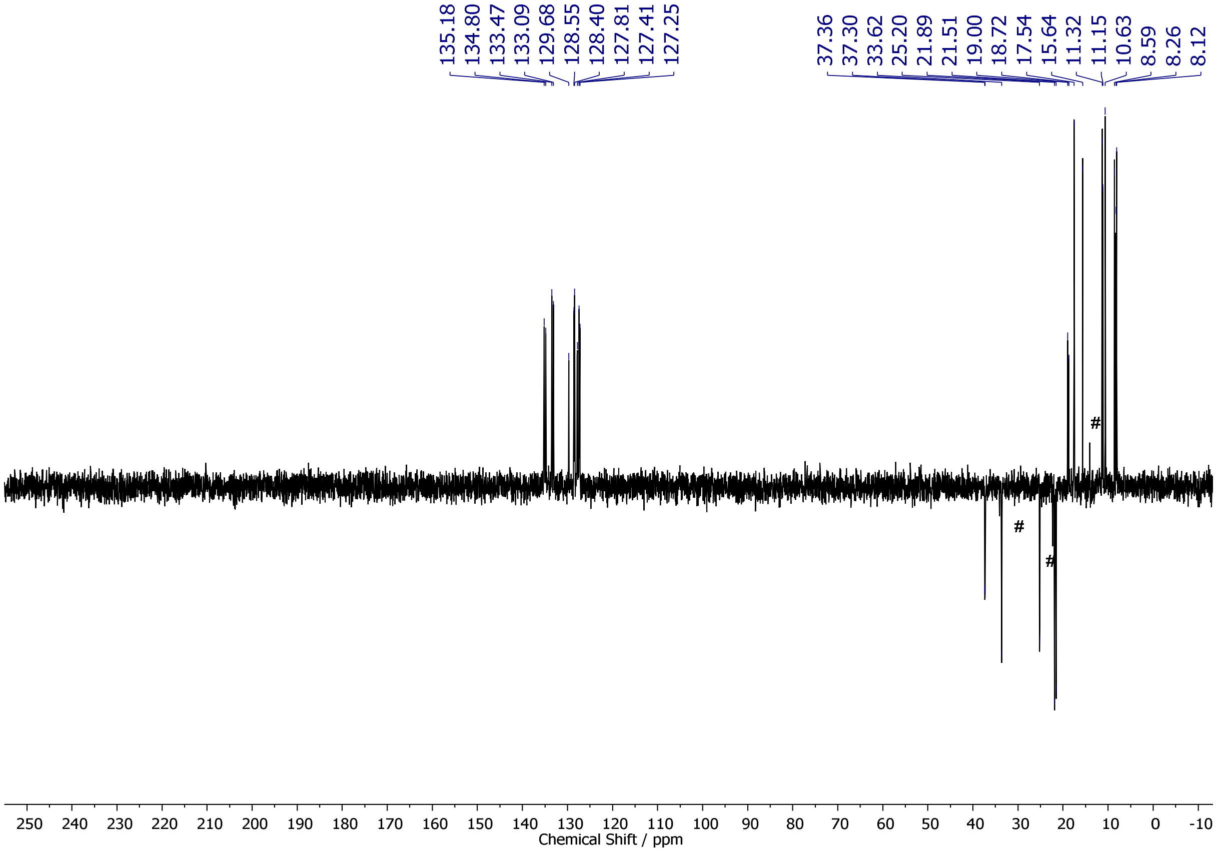

Figure S16. DEPT135 NMR spectrum of **3a** in $CDCl_3$ (∗) # Hexane/Pentane

6.2. NMR-spectra of compound **3b**

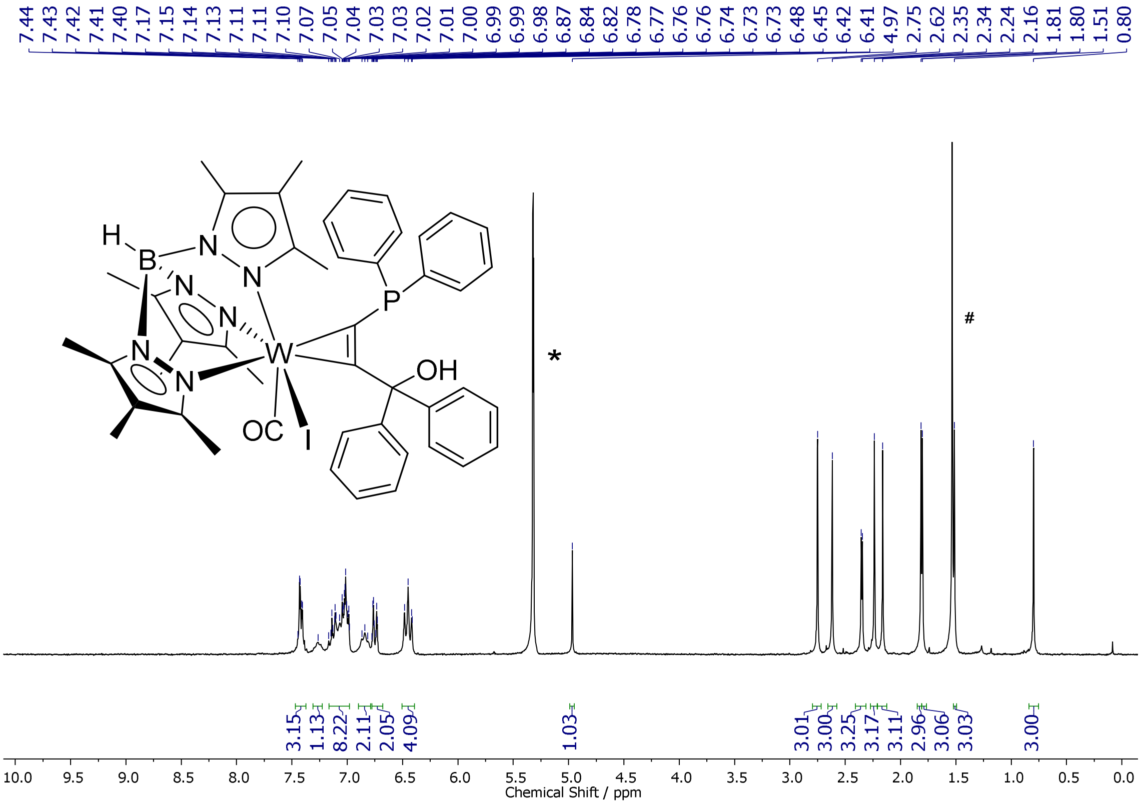

Figure S17. ^{1}H NMR of **3b** in CD_2Cl_2 (∗); + H_2O

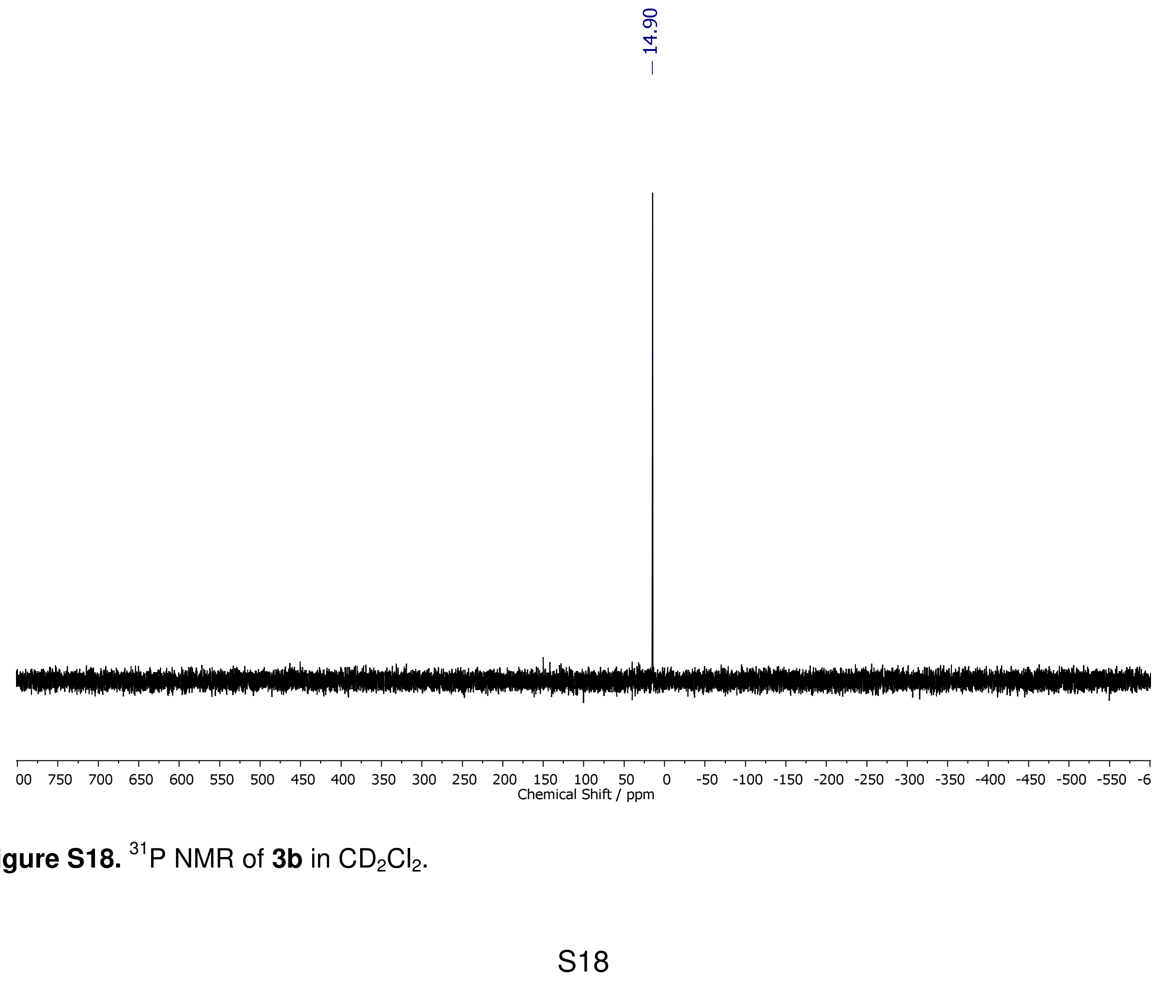

Figure S18. ^{31}P NMR of **3b** in CD_2Cl_2.

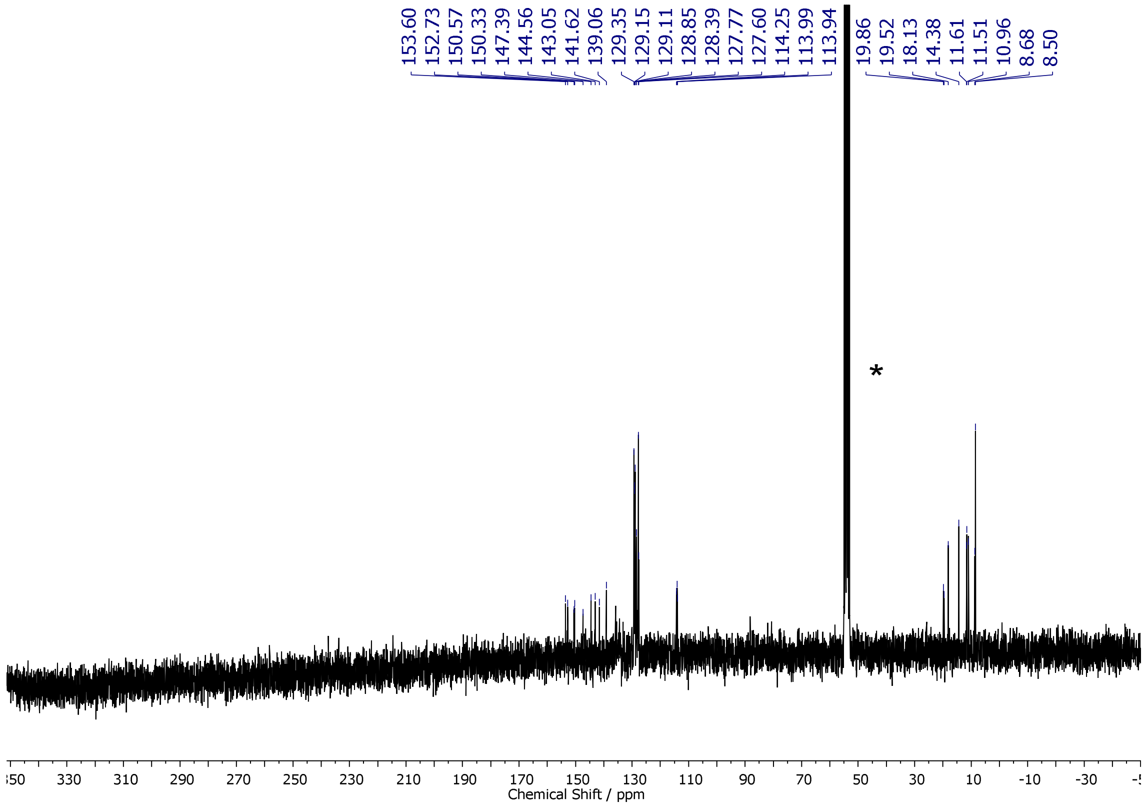

Figure S19. ^{13}C NMR of **3a** in CD_2Cl_2 (*).

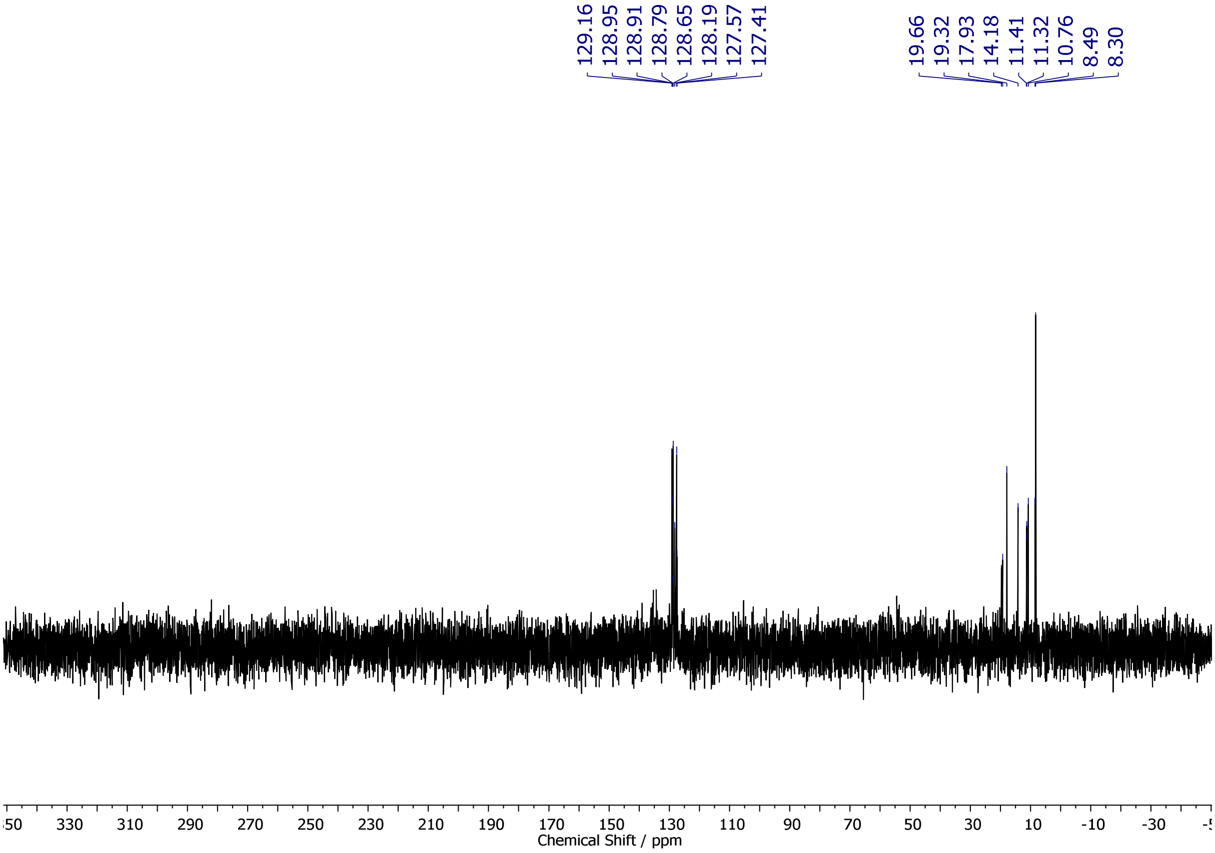

Figure S20. DEPT135 NMR spectrum of **3a** in CD_2Cl_2

6.3. NMR-spectra of compound **3c**

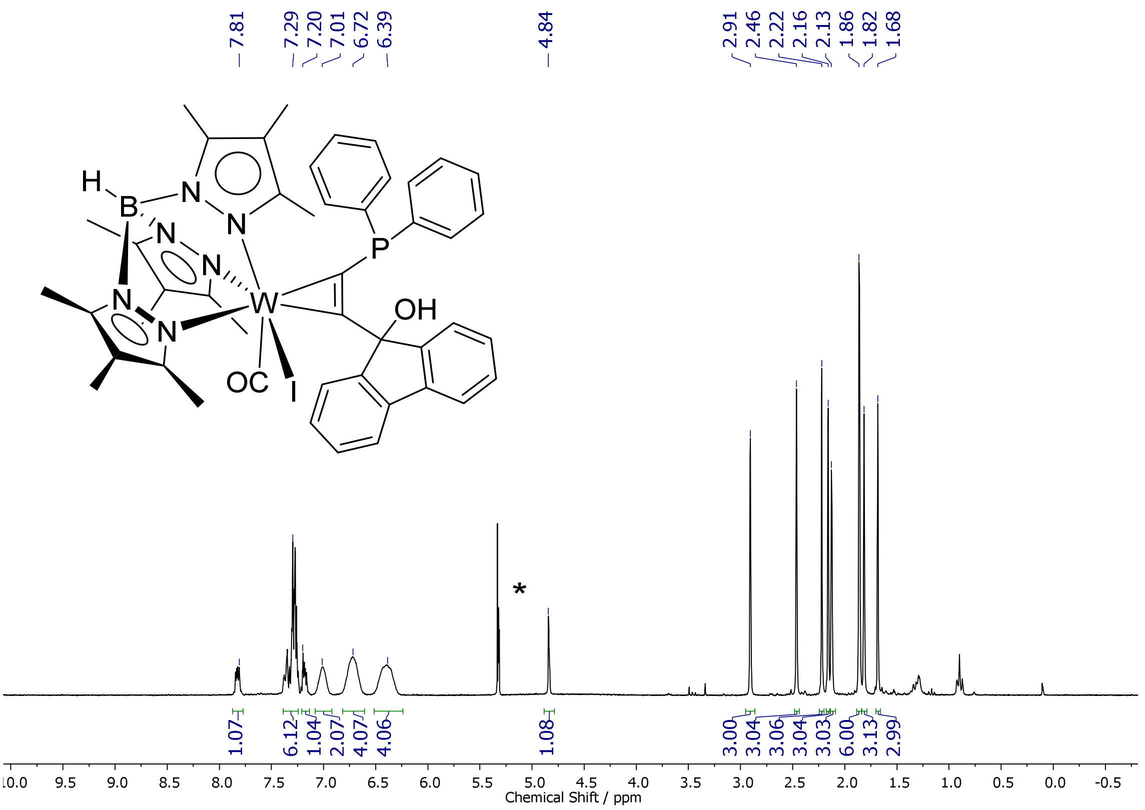

Figure S 21. ^{1}H NMR of **3c** in CD_2Cl_2 (*).

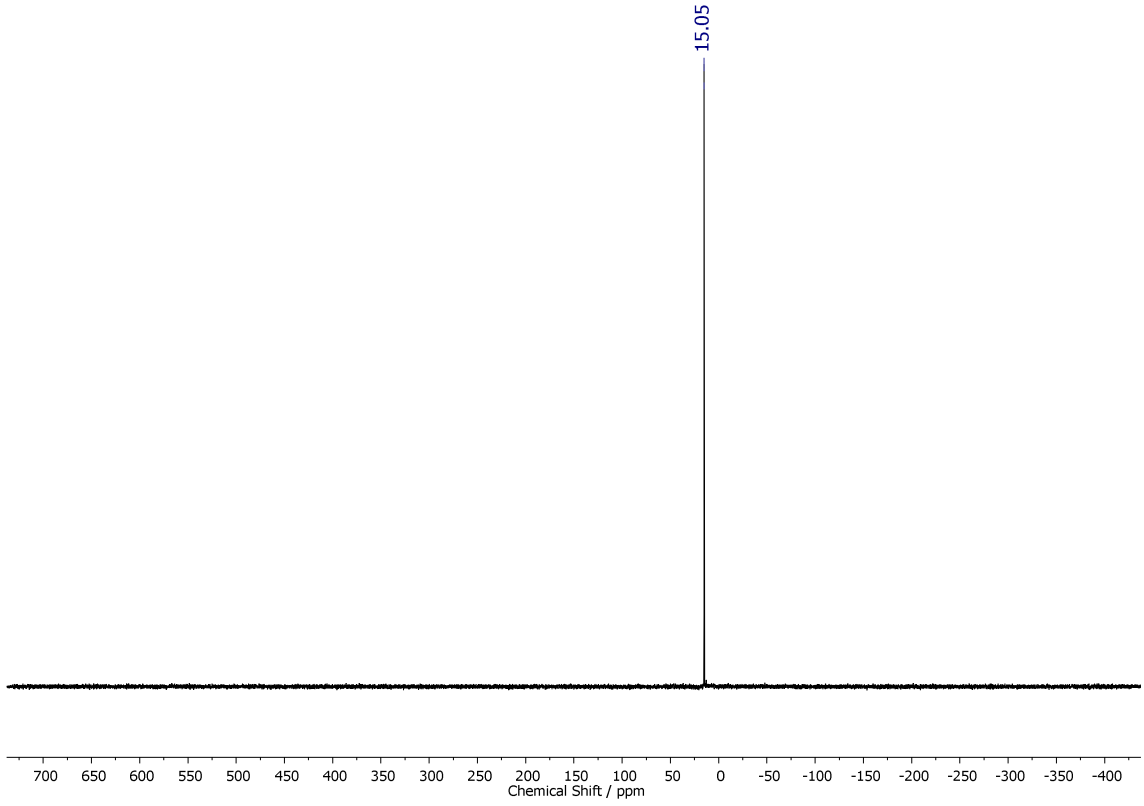

Figure S22. ^{31}P NMR of **3c** in CD_2Cl_2.

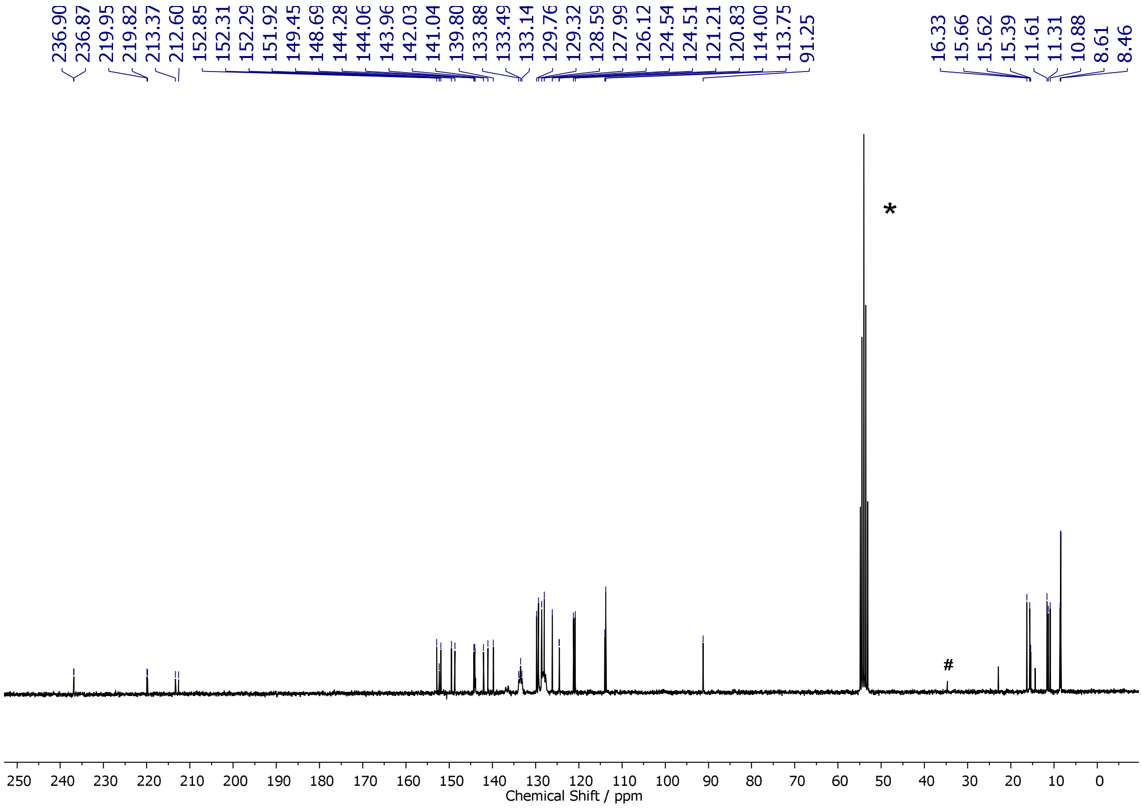

Figure S23. ^{13}C NMR of **3c** in $CDCl_3$ (*) # Pentane

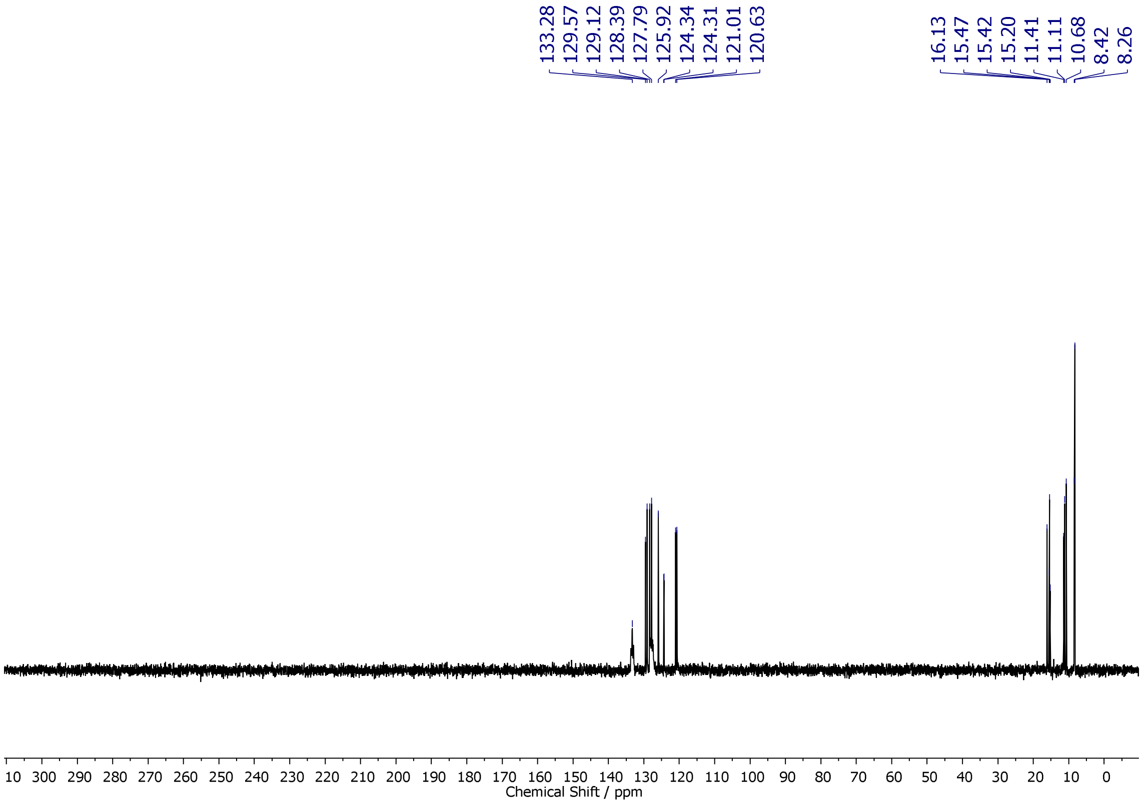

Figure S24. DEPT135 NMR spectrum of **3c** in CD_2Cl_2

6.4. NMR-spectra of compound **3d**

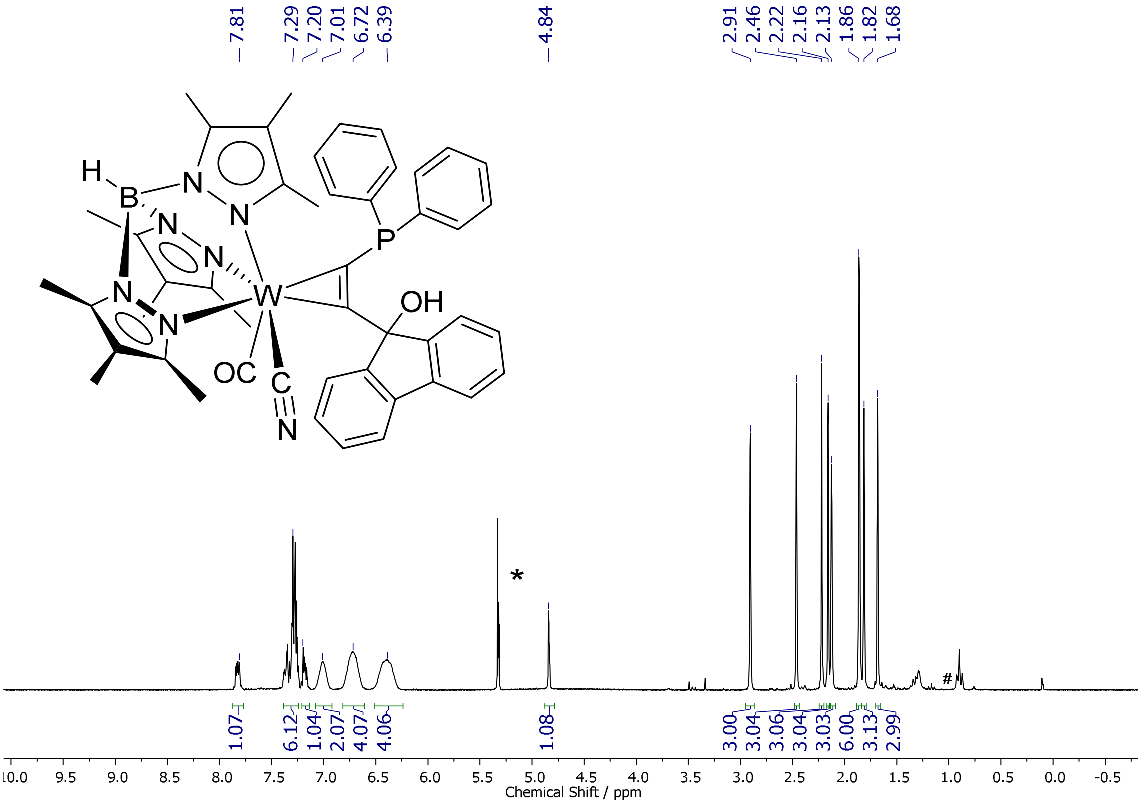

Figure S25. ^{1}H NMR of **3d** in CD_2Cl_2 (*);# *n*-pentane.

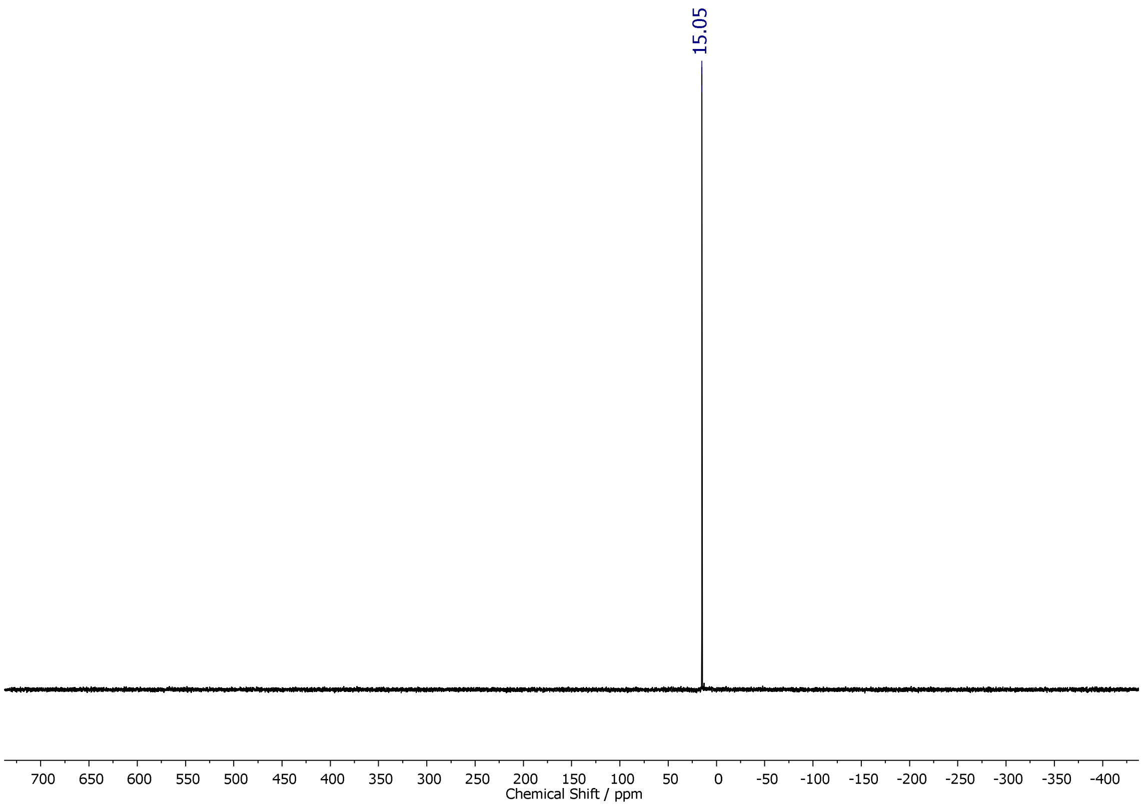

Figure S26. ^{31}H NMR of **3d** in CD_2Cl_2.

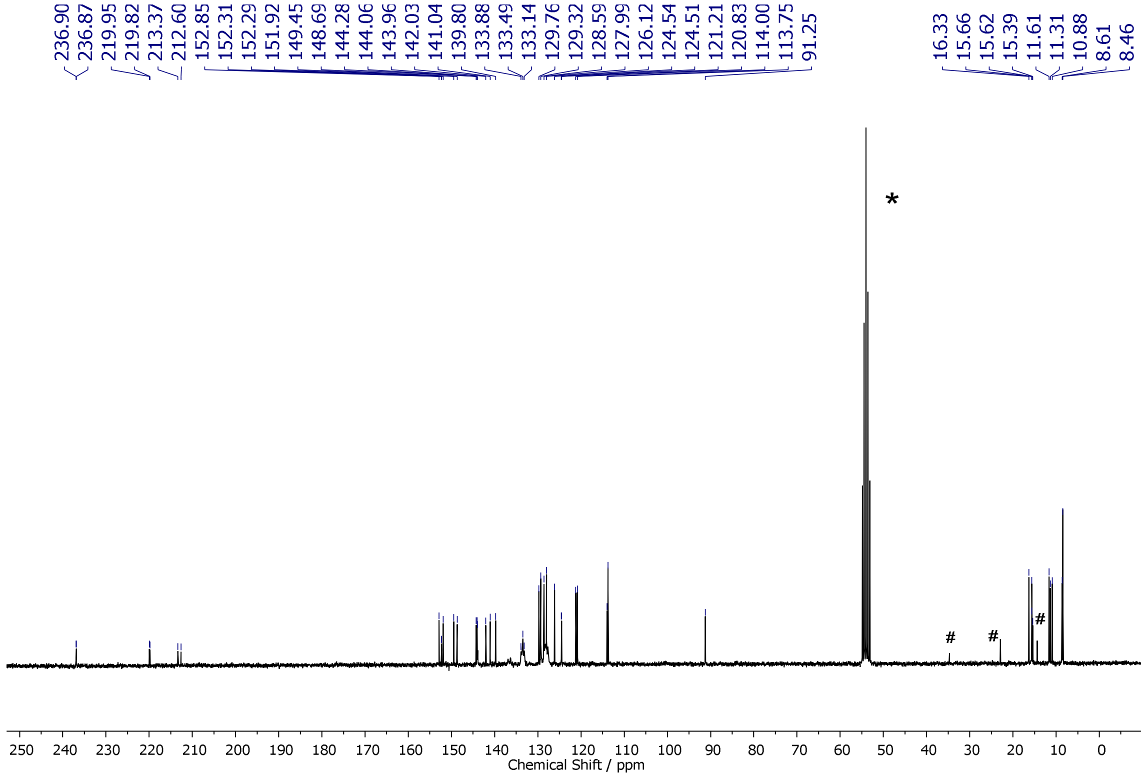

Figure S27. ^{13}C NMR of **3d** in CD_2Cl_2 (*) # *n*-Pentane.

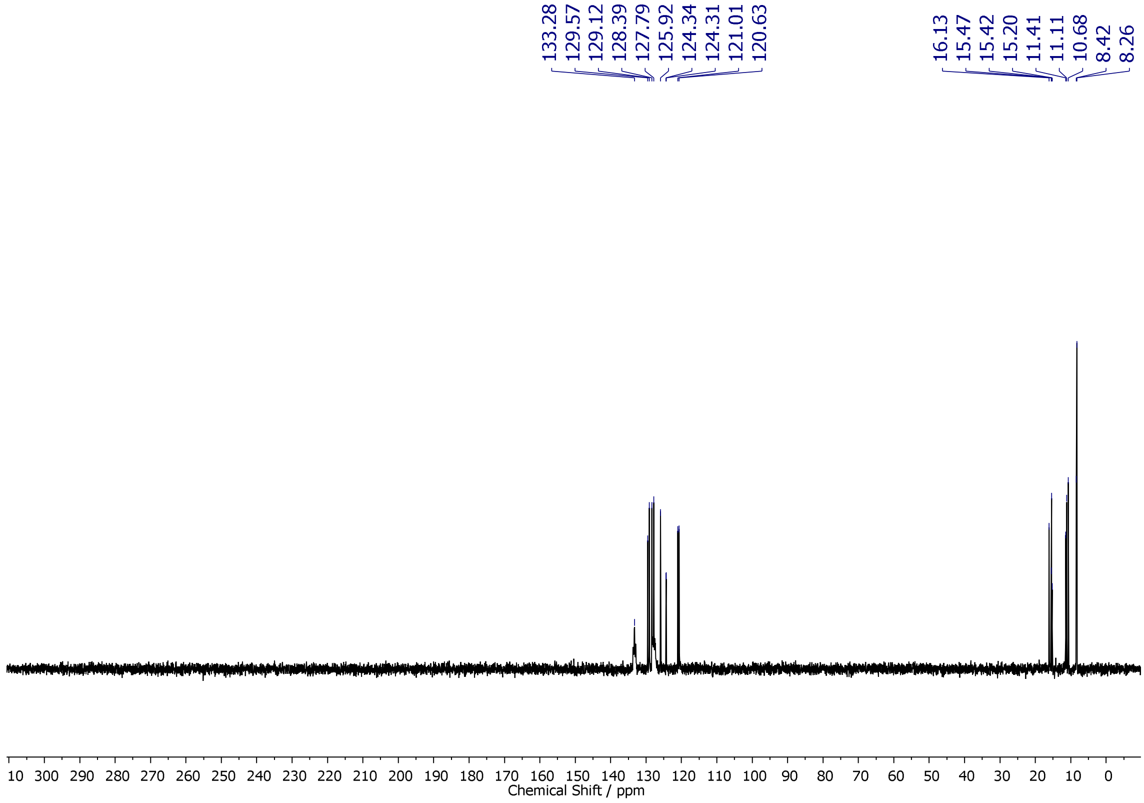

Figure S28. DEPT135 NMR spectrum of **3d** in CD_2Cl_2NMR-spectra of compound **4b**

6.5. NMR-spectra of compound **4a**

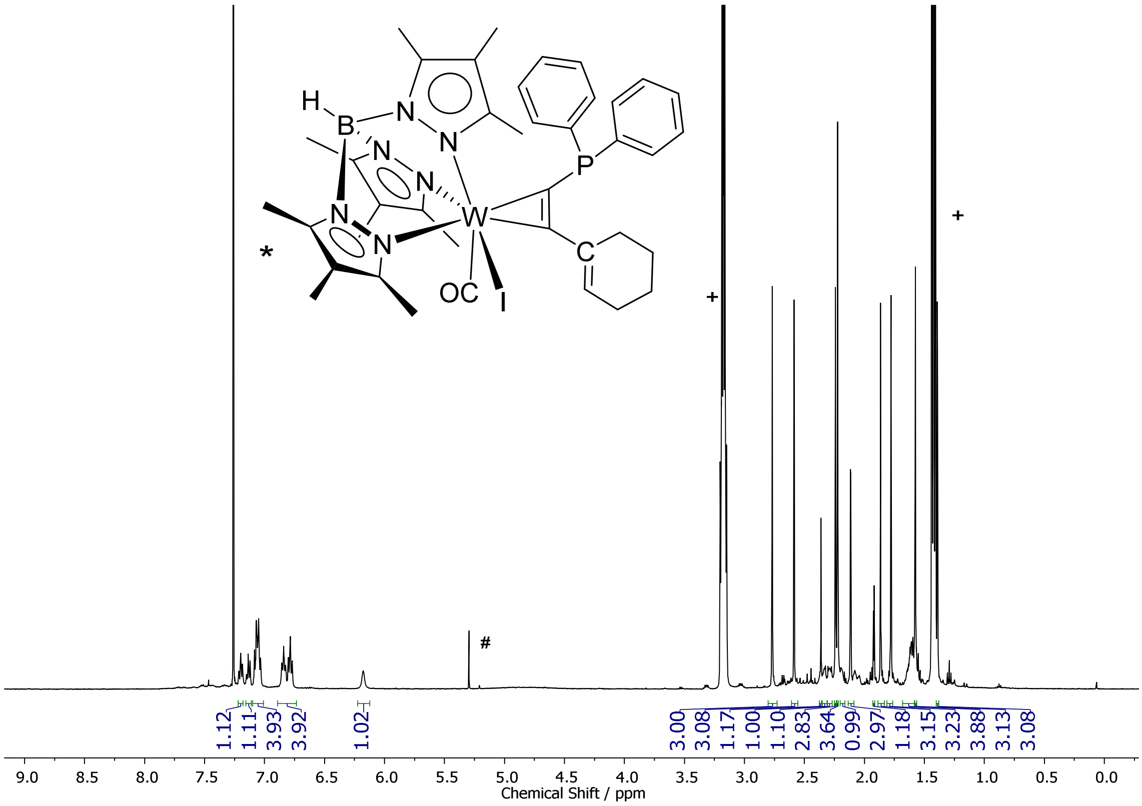

Figure S29. ^{1}H NMR of **4a** in $CDCl_3$ (∗), # CH_2Cl_2, + Et_3N.

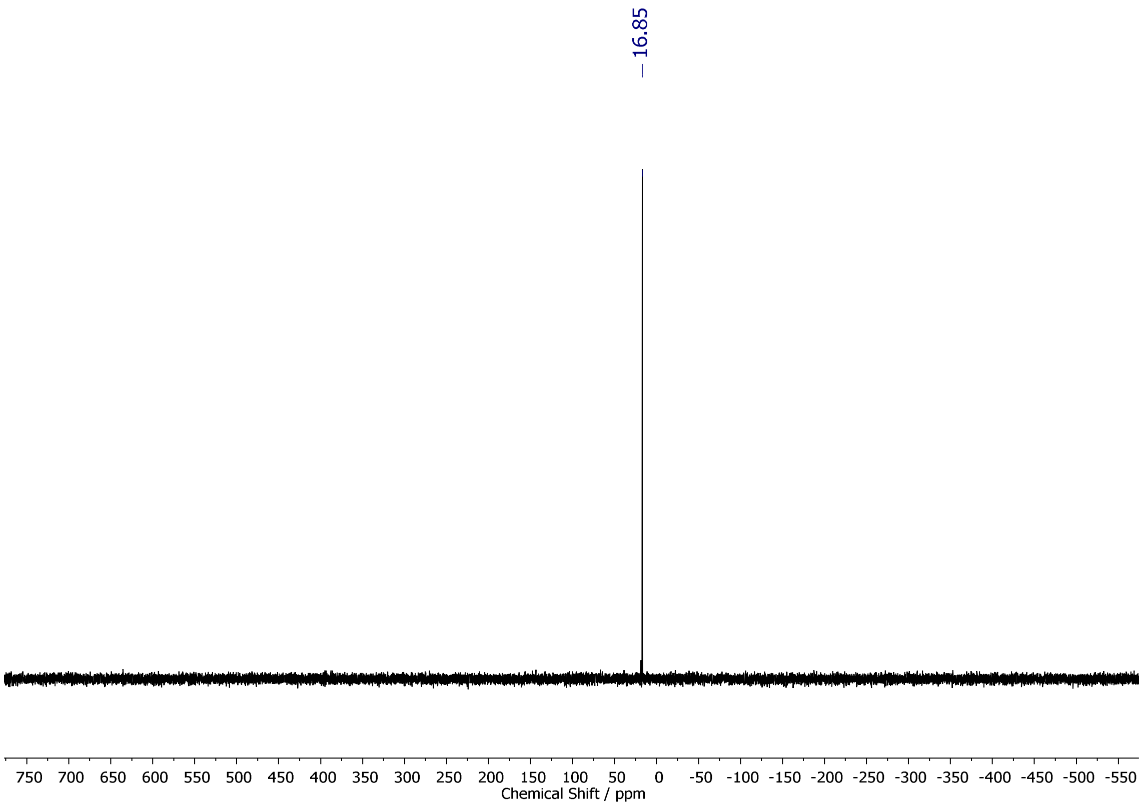

Figure S30. ^{31}P NMR of **4a** in $CDCl_3$.

6.6. NMR-spectra of compound **4b**-[(B(C_6F_5)$_4$]

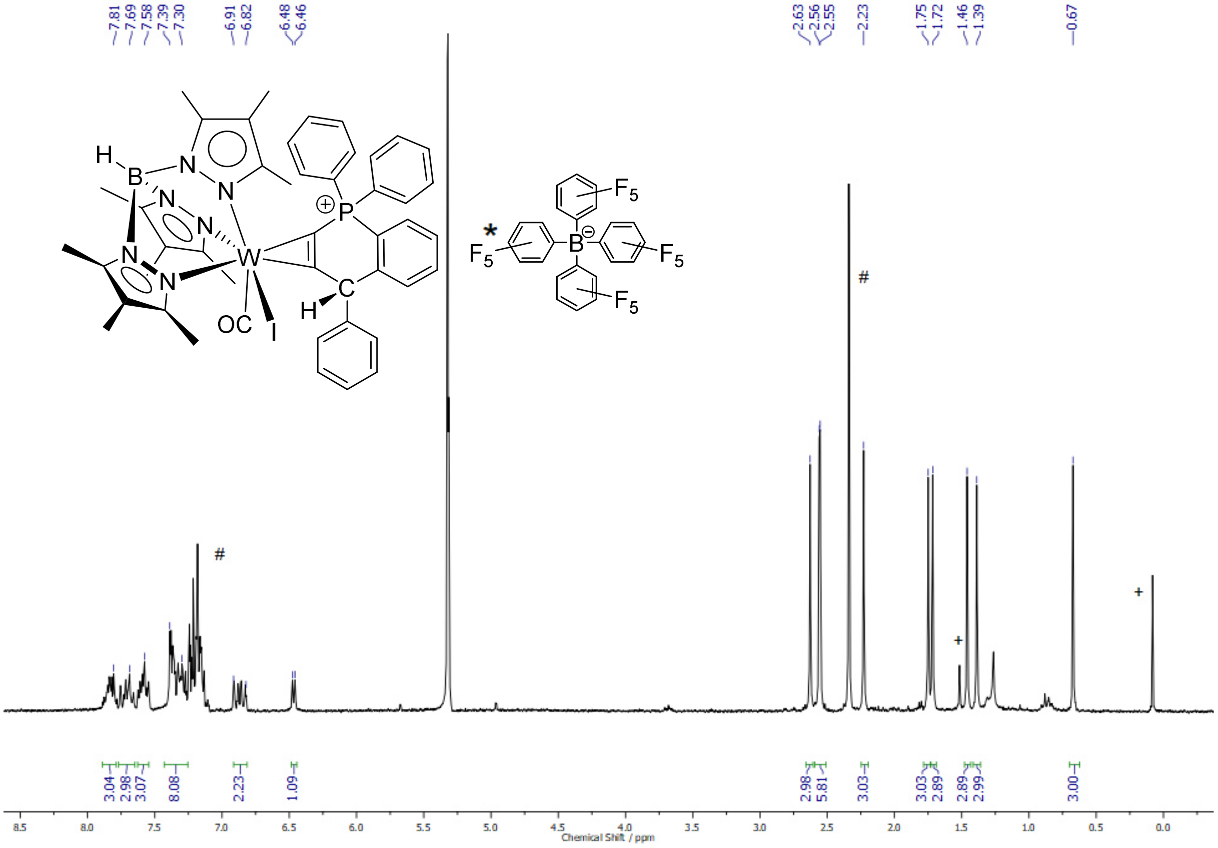

Figure S31. ^{1}H NMR of **4b**-[(B(C_6F_5)$_4$] in CD_2Cl_2 (*), # toluene, + impurity

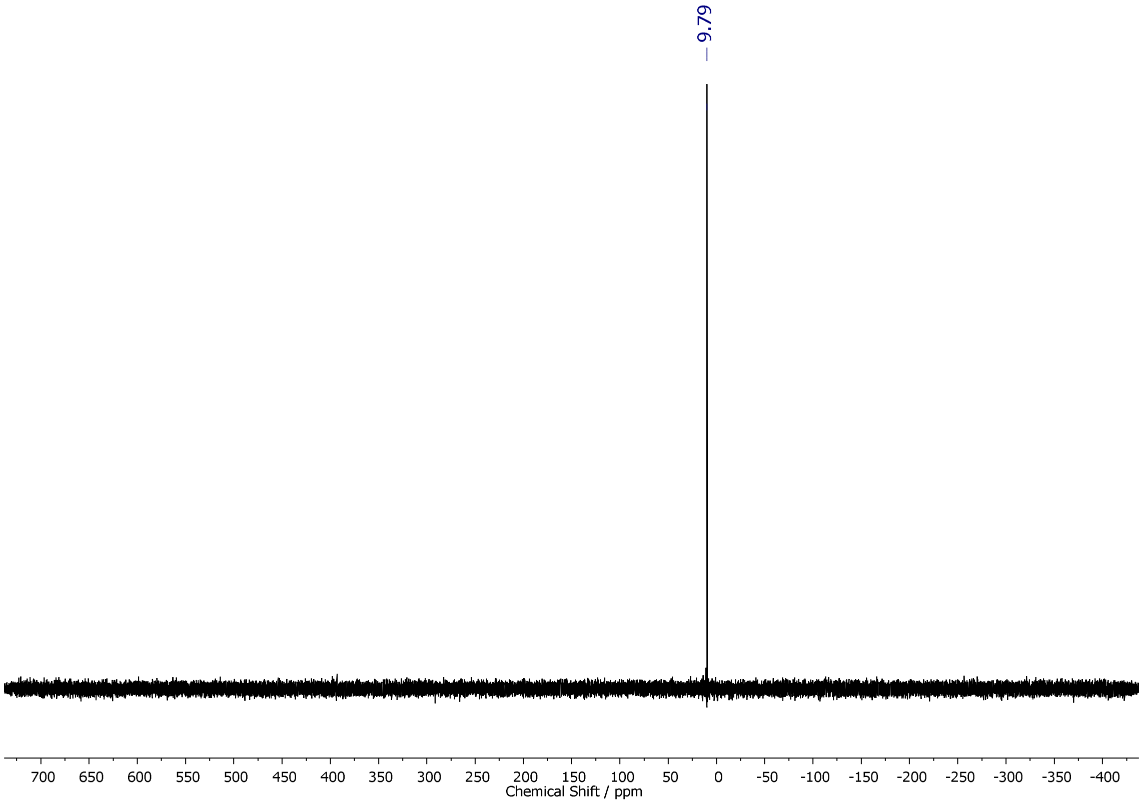

Figure S32. ^{31}P NMR of **4b**-[(B(C_6F_5)$_4$] in CD_2Cl_2.

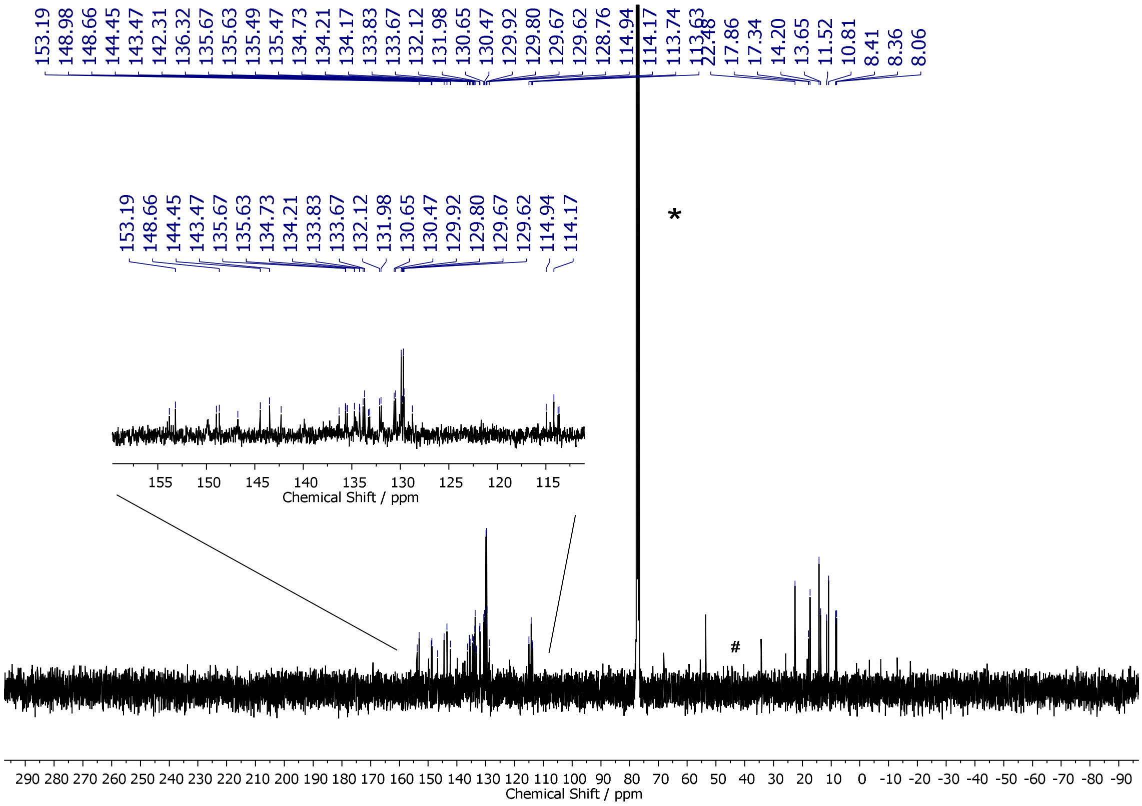

Figure S33. ^{13}C NMR of **4b**-[(B(C_6F_5)$_4$] in $CDCl_3$ (*) # impurity

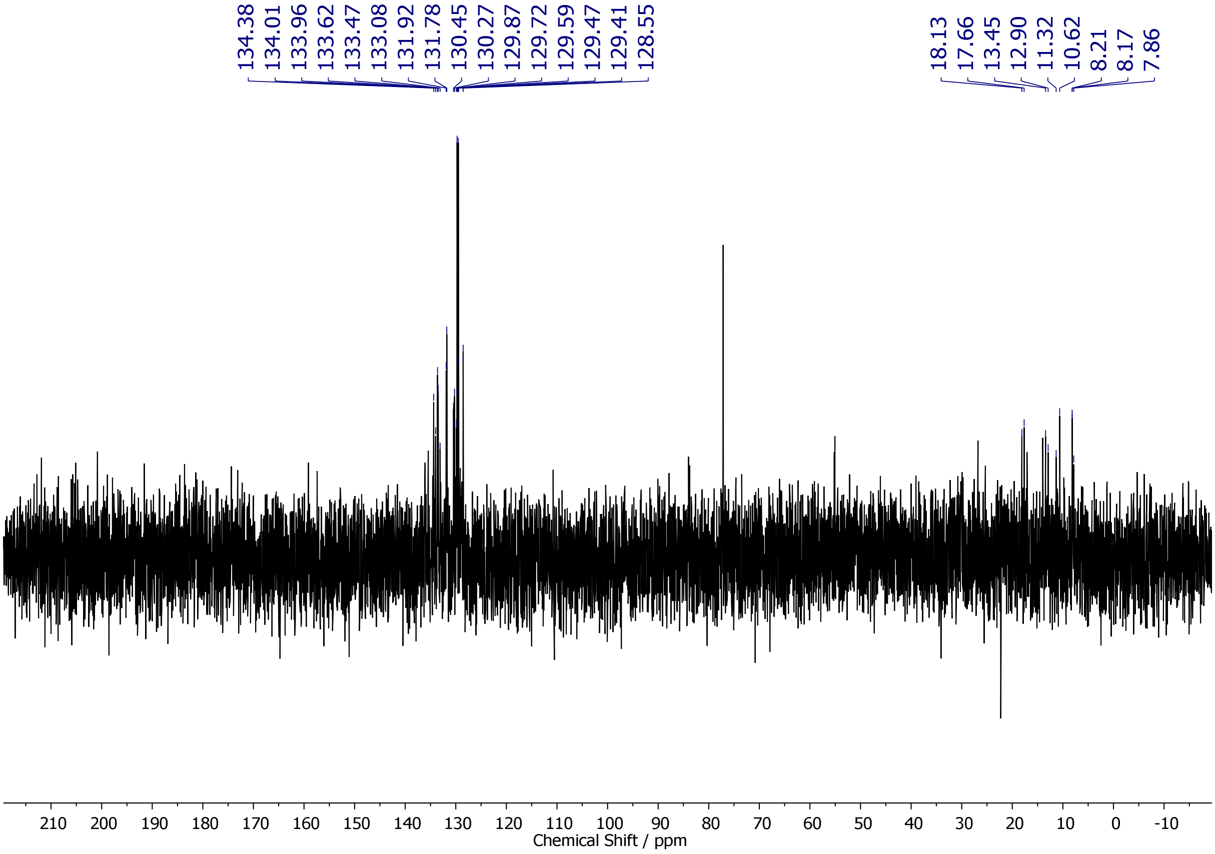

Figure S34. DEPT135 NMR spectrum of **4b**-[(B(C_6F_5)$_4$] in $CDCl_3$

6.7. NMR-spectra of compound **4c**-OTf

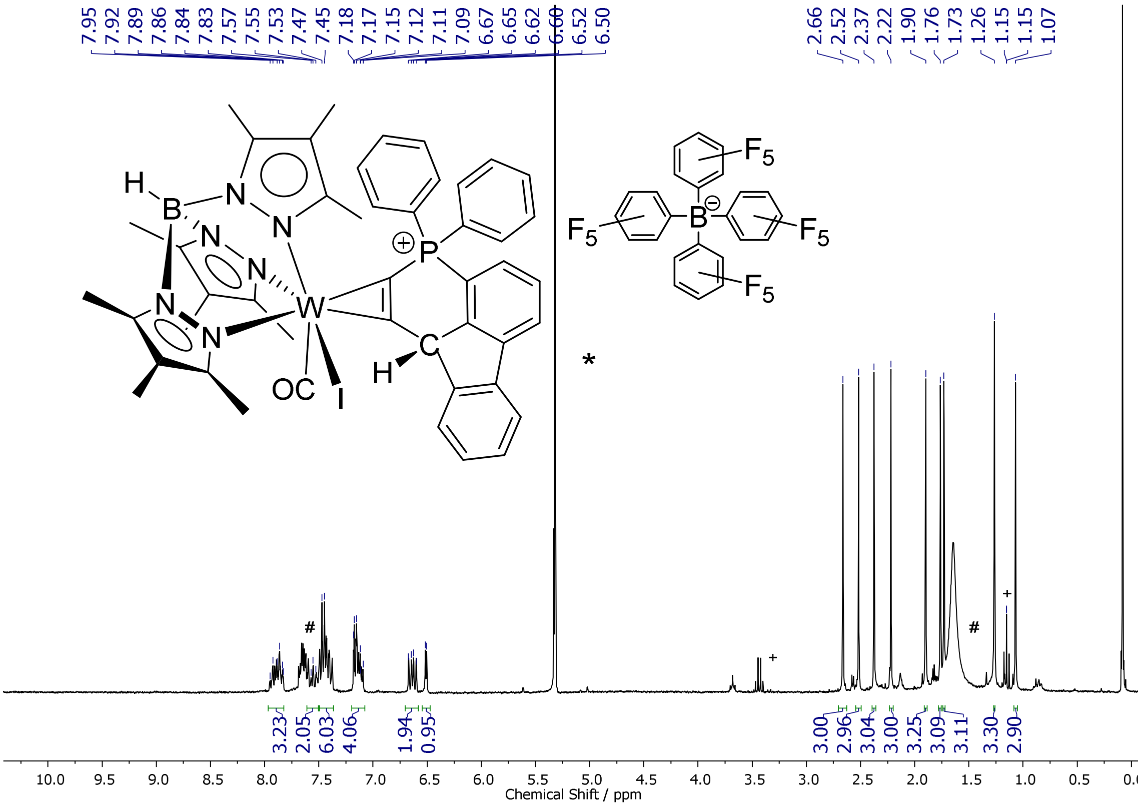

Figure S35. ^{1}H NMR of **4c**-OTf in CD_2Cl_2 (*); + Et_2O # impurity

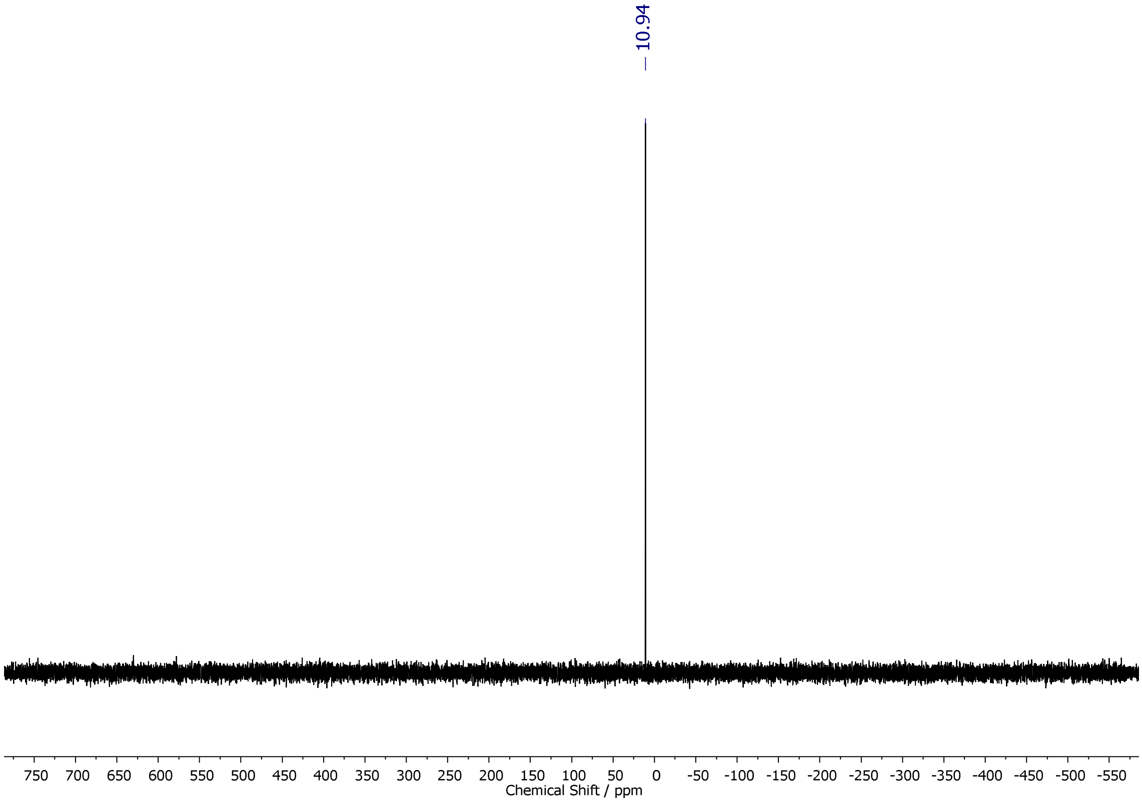

Figure S36. ^{31}P NMR of **4c**-OTf in CD_2Cl_2

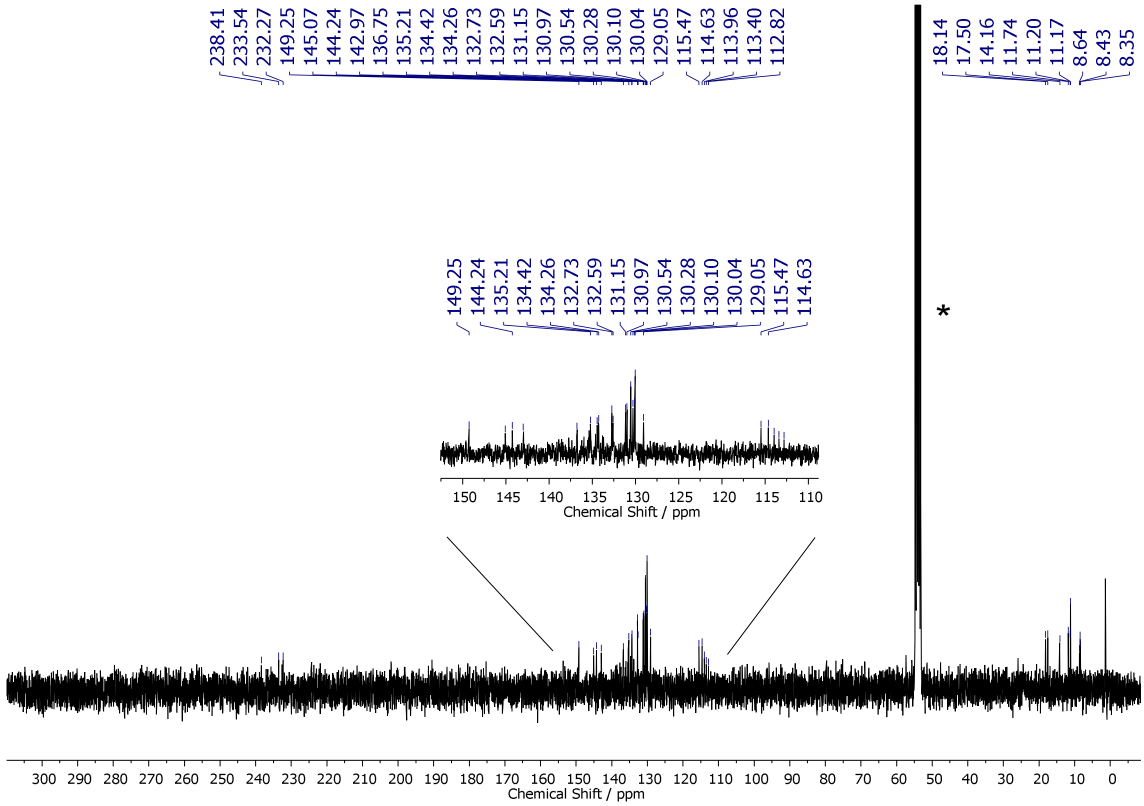

Figure S37. ^{13}C NMR of **4c**-OTf in CD_2Cl_2 (∗)

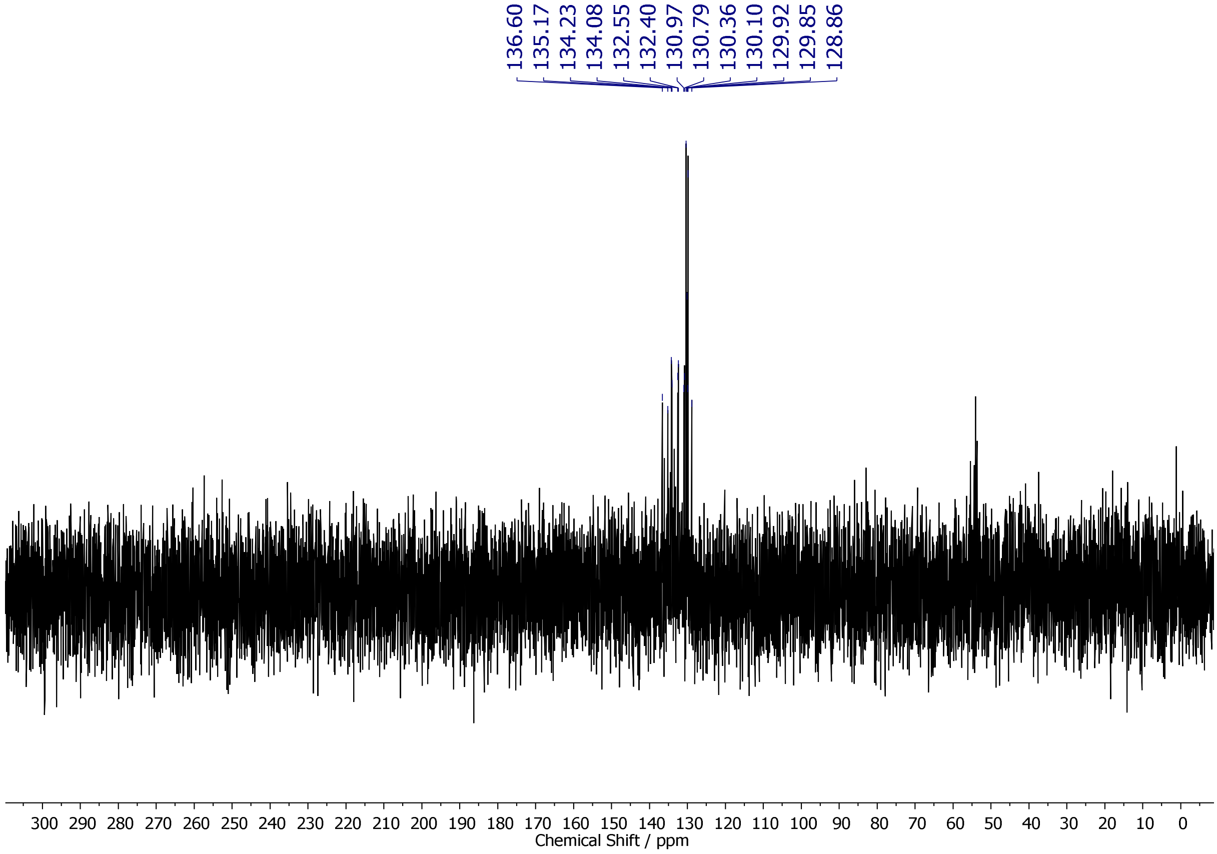

Figure S38. DEPT135 NMR spectrum of **4c**-OTf in CD_2Cl_2

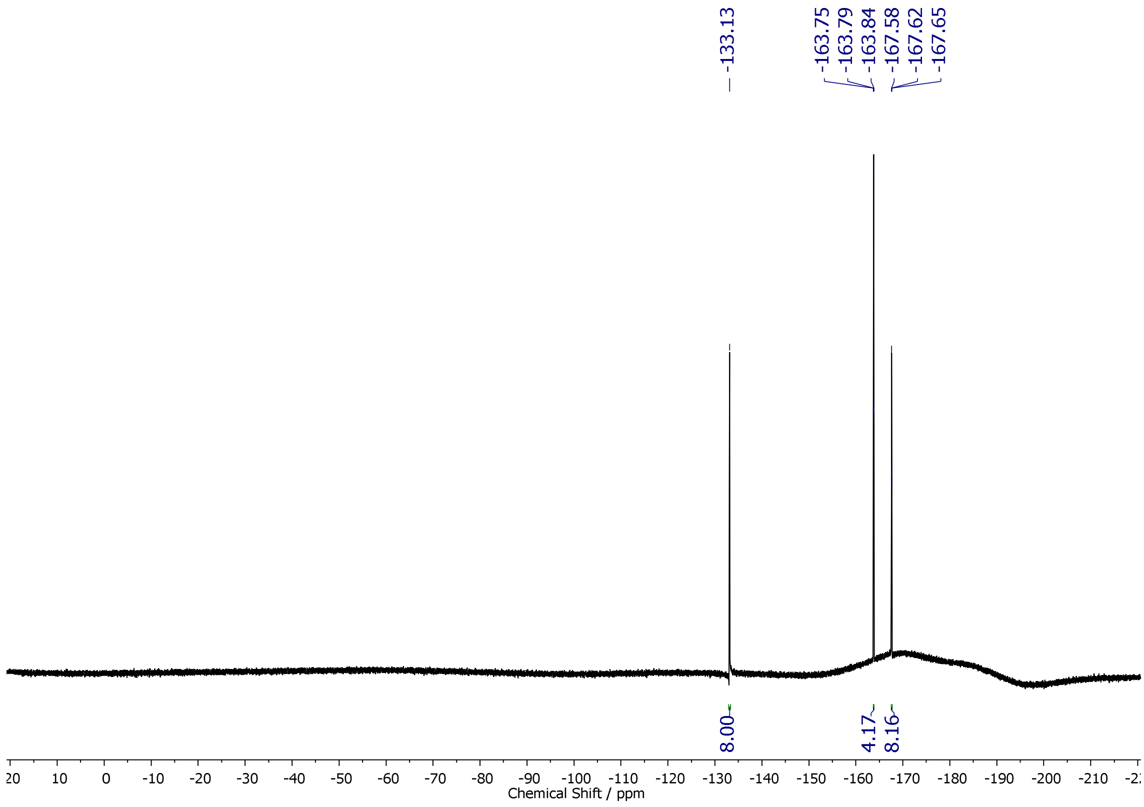

Figure S39. ^{19}F NMR of **4c**-OTf in CD_2Cl_2.

6.8. NMR-spectra of compound **5**

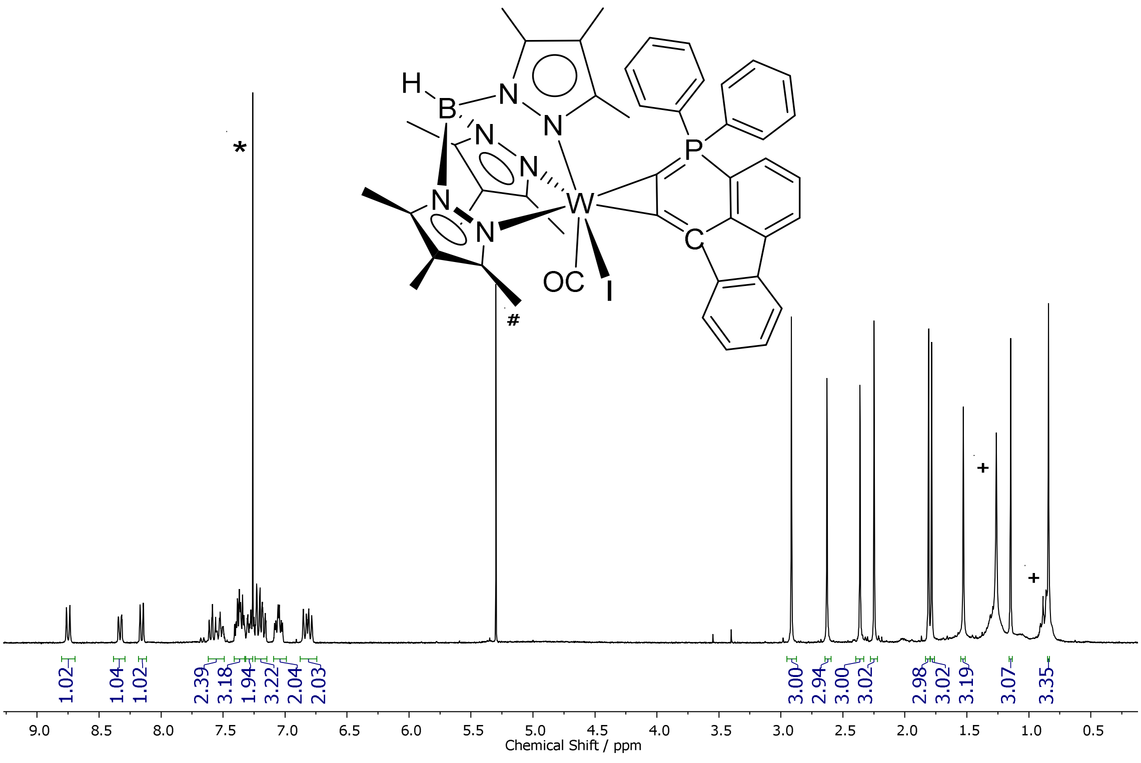

Figure S40. ^{1}H NMR of **5** $CDCl_3$ (∗), # CH_2Cl_2, + impurity

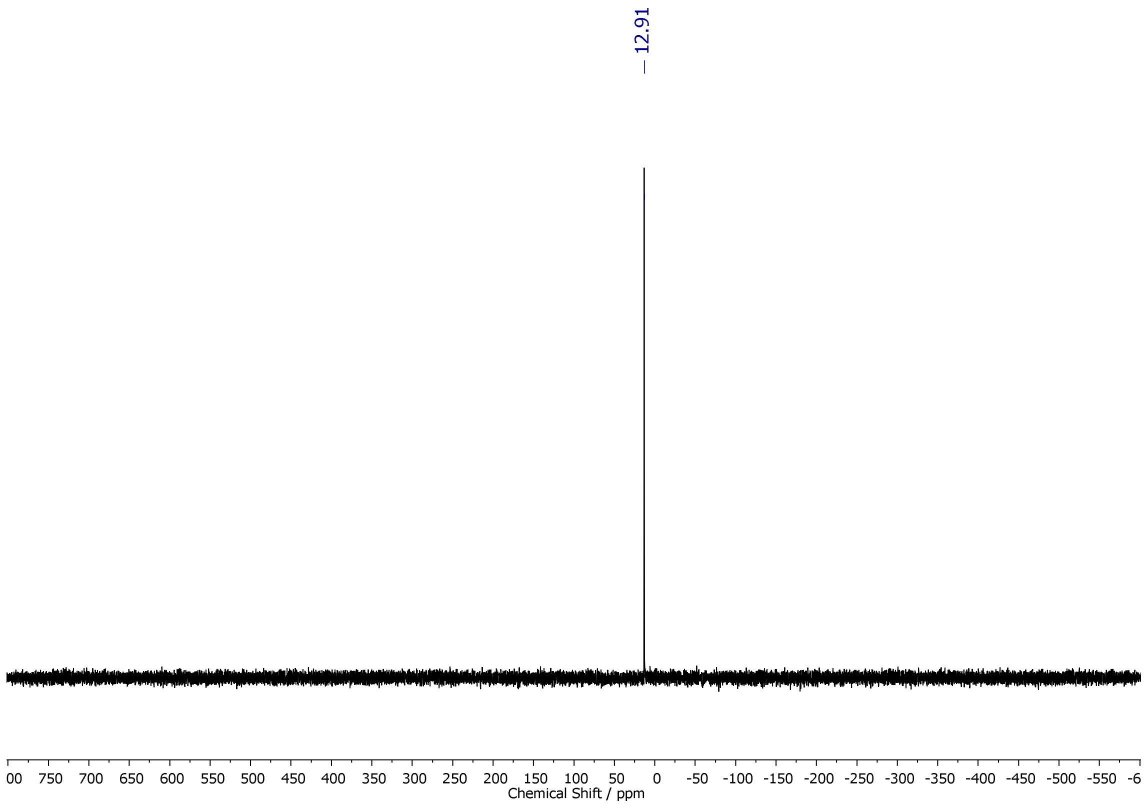

Figure S41. ^{31}P NMR of **5** in $CDCl_3$.

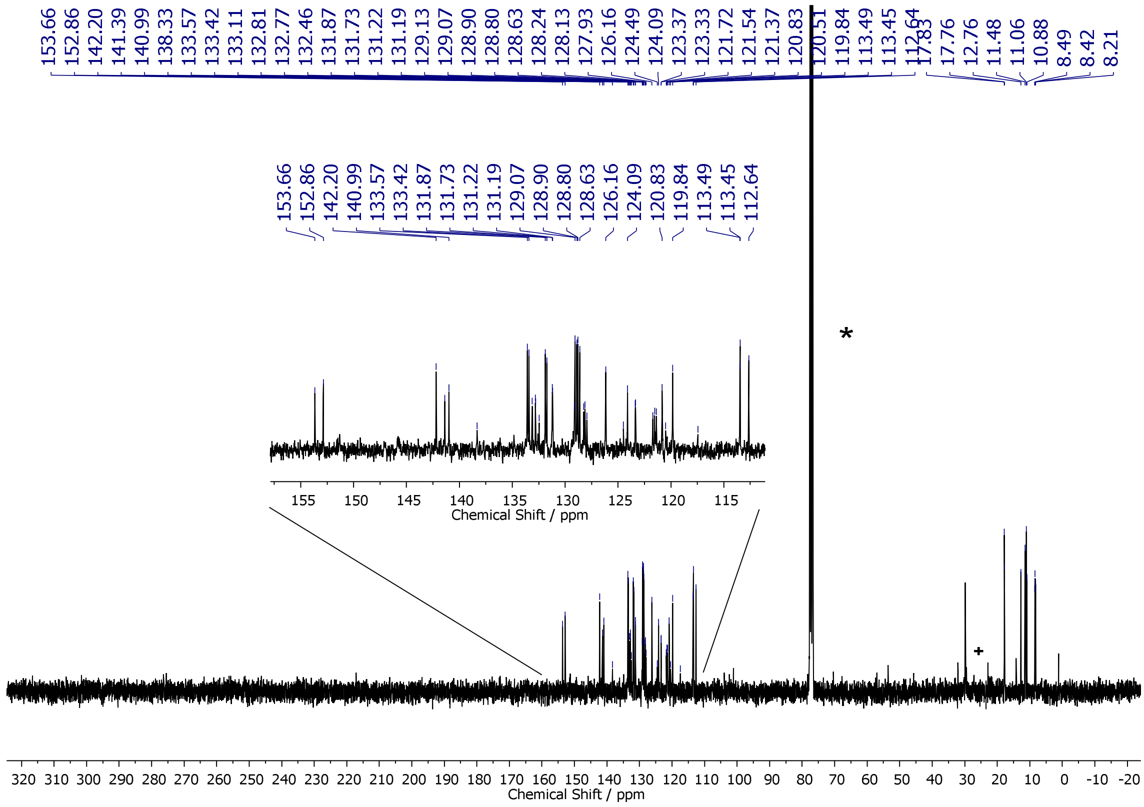

Figure S42. ^{13}C NMR of **5** in $CDCl_3$ (*) + impurity

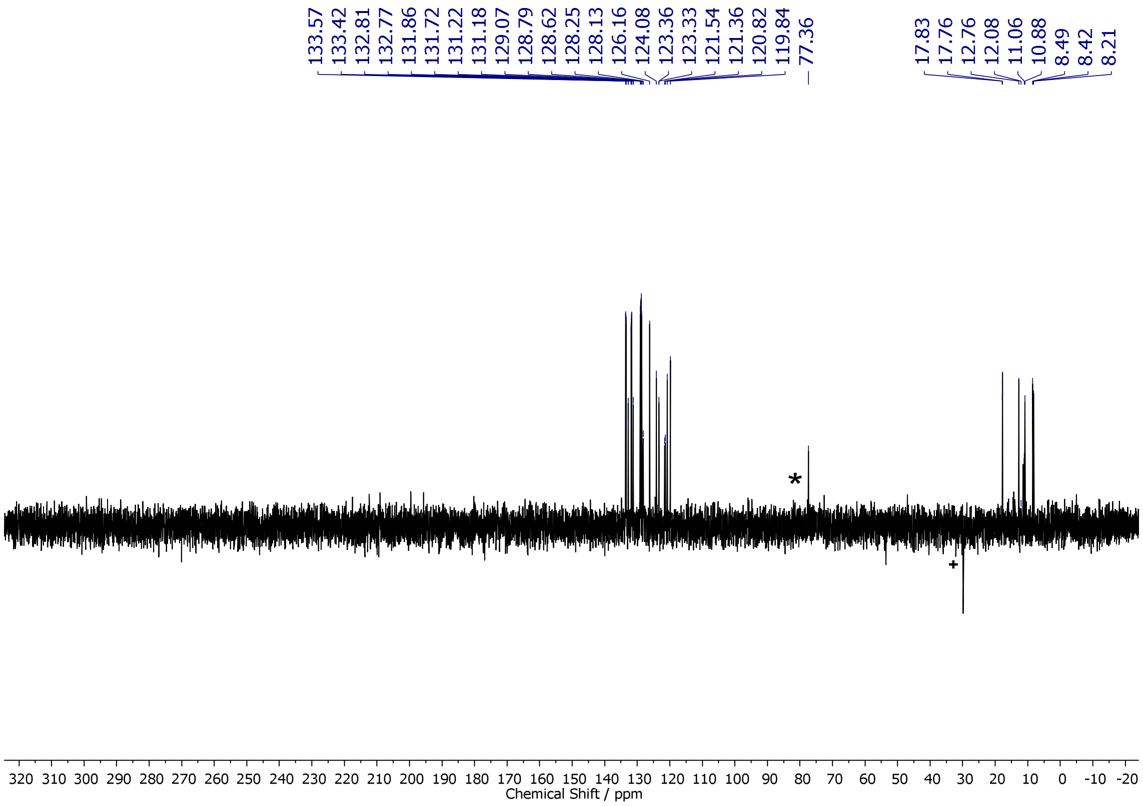

Figure S43. DEPT135 NMR spectrum of **5** in $CDCl_3$ (*), # CH_2Cl_2, + impurity

7. DFT calculation details

The calculations were carried out using either the G09RevE.01[S9] or the ORCA 4.11[S10] program package applying DFT. The molecular geometries of complex cation **4c**$^+$ and neutral **5** as well as of the potential intermediates **IM1**$^+$ (open-shell triplet and closed shell singlet), *syn*-**IM2**$^+$ and *anti*-**IM2**$^+$ (which were not isolated) were optimized without truncation and symmetry constraints in the gas phase using either the PBE0 functional[S11] or the long-range corrected CAM-b3lyp[S12] Quasi-relativistic effective core potentials of the Stuttgart/Cologne group were used for W (ECP60) and I (ECP46) in combination with (8s7p6d2f1g)/[6s5p3d2f1g] basis set for W and a (4s5p)/[2s3p] basis set for I.[S13] Split valence triple ζ-basis sets (def2-TZVP) of the Ahlrich group were used for the other elements.[S14] Dispersion was accounted for by the atom-pairwise dispersion correction with the Becke-Johnson damping scheme (D3BJ).[S15] In doing so a reasonable match between the calculated and the experimentally determined structures for the cyclization product **4c**$^+$ was achieved. A comparison of calculated and experimentally determined metric parameters for **4c**$^+$ is given in Table S4. The strongest deviations (1.29 %, 1.22 %) arise for the coordinative bonds W–I and W–N with CAM-b3lyp, while most deviations are well below 1 %. Frequency calculations were performed with a smaller def2-SVP basis set in order to identify

all stationary points as minima. The final enthalpies (ΔH) were calculated using the total electronic energy from the higher level calculation and the thermal correction to enthalpy from the frequency calculation. TD-DFT calculations for complex **5** and extraction of the difference densities were performed with Orca (Figure S44).

Table S4. Comparison of essential bond lengths [Å] for **4c**$^+$ obtained by X-ray structure analysis and single molecule geometry optimization in the gas phase, the calculated bond lengths were rounded for clarity.

	**4c**$^+$ (calc., PBE0)	**4c**$^+$ (calc., CAM-b3lyp)	**4c**$^+$ (exp. in **4c**-OTf)
W1–C1	2.002	2.006	2.009(4)
W1–C2	2.044	2.050	2.050(4)
W1–I1	2.7894	2.8112	2.7773(3)
W1–N1	2.259	2.274	2.245(3)
W1–N3	2.187	2.206	2.184(3)
W1–N5	2.215	2.227	2.221(3)
W1–C3	1.981	1.997	1.980(4)
C1–C2	1.340	2.328	1.341(6)
C1–P1	1.732	1.735	1.753(4)
C2–C16	1.489	1.492	1.501(6)

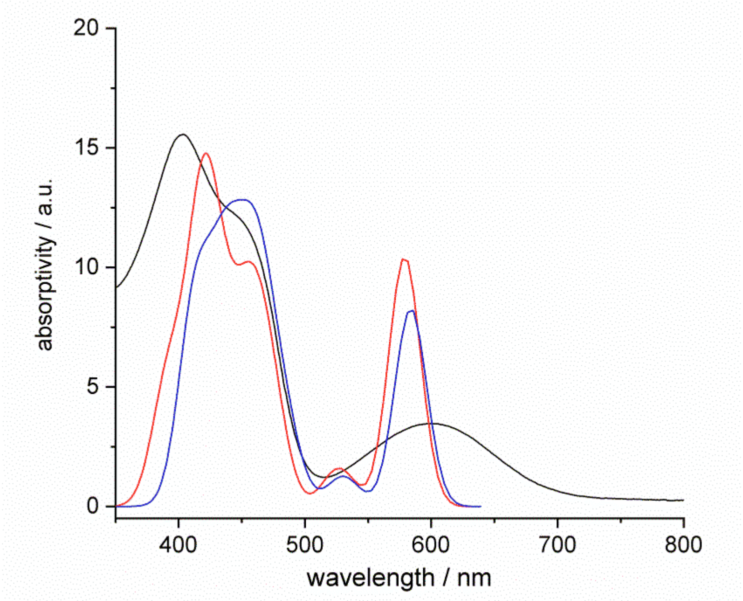

Figure S44. Comparison calculated and experimental absorption spectrum of **5**; experimental (black), calculated PBE0 (blue), calculated b3lyp (red); Gaussian line shape with 30 nm peak width, energy shift –16 nm for b3lyp and +31 nm for PBE0.

Natural resonance theory analysis (NRT):

The implementation of NRT to transition metal complexes frequently poses problems, which arise from the orthogonalization procedure for the d-orbitals. The identification of a starting reference resonance structure for complex **5** turned out to be impossible. Hence, a simplified, truncated model complex bearing NH_3/NH_2^- ligands was used for the analysis. NBO6, the standard functional b3lyp and a SDD basis set were used. Due to the strong delocalization full density matrix (FDM) and a stabilization threshold of 30 kcal/mol were applied. Nine leading resonance structures for the optimized structure (Figure S45) are depicted in Figure S46.

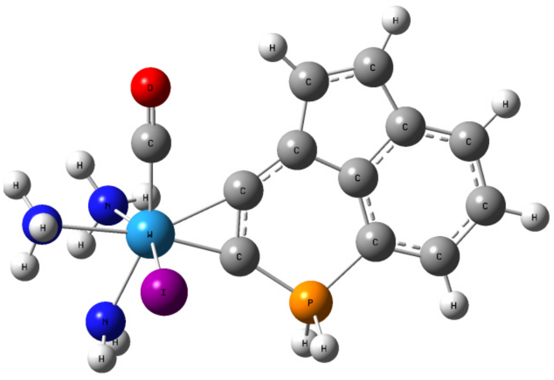

Figure S45. Optimized molecular structure of the model phosphinyne complex.

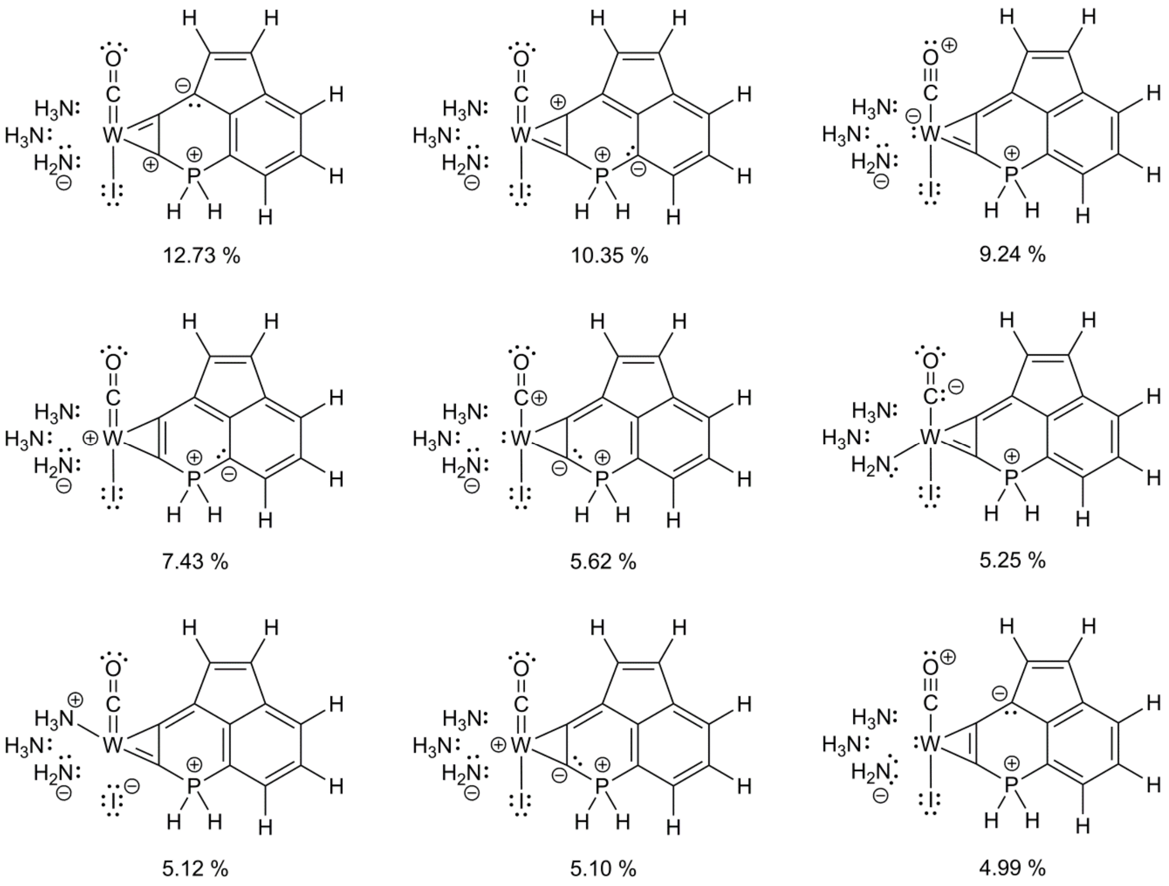

Figure S46. Leading resonance structures derived from natural resonance theory.

Table S5. Cartesian coordinates of the geometry optimized structures of [1]**IM1⁺** in the gas phase; PBE (left) CAM-b3lyp (right), def2-TZVP/ECP(W, I).

C	-3.582220	-4.222217	-1.321570	C	-3.746132	-3.939055	-1.680504
C	-2.829426	-3.109307	-0.965308	C	-2.943592	-2.902391	-1.220435
C	-4.877378	-4.376747	-0.852154	C	-5.024918	-4.107629	-1.184594
C	-3.400614	-2.157711	-0.126736	C	-3.458943	-2.046108	-0.259888
C	-5.466752	-3.417501	-0.017907	C	-5.554610	-3.242936	-0.215988
C	-2.873254	-0.916989	0.386348	C	-2.865458	-0.897625	0.377737
C	-4.729551	-2.315625	0.337268	C	-4.770387	-2.220242	0.236495
C	-1.588671	-0.404429	0.160272	C	-1.569543	-0.412528	0.163968
C	-3.924203	-0.299354	1.173200	C	-3.861383	-0.348630	1.283482
C	-5.054484	-1.151058	1.162743	C	-5.021211	-1.149510	1.216932
W	0.424084	-0.766088	-0.025214	W	0.453956	-0.787438	-0.016779
C	-0.903329	0.750816	0.060132	C	-0.889017	0.731895	0.065927
C	-3.957989	0.895662	1.881202	C	-3.821648	0.760484	2.111134
C	-6.194986	-0.812807	1.850523	C	-6.120408	-0.853885	1.975790
I	0.267339	-1.779283	-2.606473	I	0.411859	-1.850947	-2.629299
N	1.737157	0.740238	-0.917299	N	1.760979	0.760068	-0.930011
N	1.169219	-0.063661	1.891965	N	1.163120	-0.086802	1.929569
N	2.315034	-1.951462	0.149374	N	2.397170	-1.886013	0.219287
C	-0.307375	-2.467511	0.817817	C	-0.310068	-2.518989	0.700184
P	-1.221741	2.428056	0.465620	P	-1.257012	2.409536	0.468465
C	-2.657769	2.903328	-0.526740	C	-2.769397	2.776329	-0.464107
C	-3.309550	4.089508	-0.184700	C	-3.568288	3.818837	-0.002350
C	-3.169312	2.116439	-1.555604	C	-3.183819	2.048854	-1.574734
C	-4.442156	4.488630	-0.873744	C	-4.751878	4.134941	-0.644486
C	-4.312914	2.511177	-2.232053	C	-4.374827	2.356881	-2.207945
C	-4.948593	3.697003	-1.895381	C	-5.159278	3.400626	-1.745673
C	0.442677	1.640687	-2.876215	C	0.497674	1.568898	-2.953713
C	1.638084	1.515799	-2.012610	C	1.675757	1.485897	-2.054605
C	2.839730	2.196086	-2.211044	C	2.862746	2.192297	-2.247641
N	2.977145	0.932218	-0.407705	N	2.978588	1.015989	-0.390157
C	3.142256	3.135149	-3.321996	C	3.166276	3.097746	-3.390896
N	3.444970	-1.315869	0.541116	N	3.484806	-1.187184	0.642960
C	2.631246	-3.253762	0.003431	C	2.781093	-3.170336	0.111491
C	4.461691	-2.184732	0.639724	C	4.531773	-2.008645	0.794487
C	0.140808	3.511604	-0.013533	C	0.019989	3.537935	-0.134556
C	0.048605	4.448415	-1.040111	C	-0.166755	4.403897	-1.205240
C	1.267495	3.501374	0.809283	C	1.178358	3.639002	0.631587
C	1.085233	5.341729	-1.258157	C	0.803589	5.339109	-1.519454
C	2.308403	4.381728	0.573908	C	2.152379	4.561744	0.304297
C	2.218942	5.304362	-0.459080	C	1.965696	5.415255	-0.770636
C	-5.112890	1.231669	2.577756	C	-4.937459	1.056323	2.884366
C	-6.215971	0.391697	2.561423	C	-6.066837	0.262403	2.817616
O	-0.665424	-3.374611	1.405339	O	-0.748500	-3.470493	1.142601
N	2.427776	0.416143	1.981005	N	2.357222	0.549284	2.001022

C	0.572060	0.141194	3.075630	C	0.587277	0.005844	3.132110
C	3.981790	-3.441972	0.300807	C	4.124060	-3.289479	0.463553
C	1.707079	-4.340269	-0.400370	C	1.922822	-4.311048	-0.300697
C	5.829262	-1.776517	1.043843	C	5.867994	-1.533672	1.241706
B	3.427665	0.182533	0.839960	B	3.405873	0.323499	0.894575
C	2.639759	0.924447	3.207596	C	2.533119	1.048076	3.235664
C	1.469978	0.776870	3.939993	C	1.414359	0.736378	3.989321
C	-0.822867	-0.249203	3.381103	C	-0.727585	-0.591842	3.472209
C	3.940414	1.512417	3.611775	C	3.764909	1.781465	3.630259
C	1.201560	1.185410	5.345528	C	1.132999	1.075991	5.414490
C	3.663537	1.799132	-1.166562	C	3.662838	1.867216	-1.167563
C	5.054815	2.210955	-0.862606	C	5.034944	2.333892	-0.838319
C	4.717190	-4.733487	0.247842	C	4.920810	-4.549846	0.466307
H	-3.152639	-4.971800	-1.974404	H	-3.364010	-4.617283	-2.430062
H	-1.824826	-2.982902	-1.345497	H	-1.946127	-2.767997	-1.611000
H	-5.448381	-5.252300	-1.138119	H	-5.633739	-4.923666	-1.550402
H	-6.483501	-3.552553	0.331673	H	-6.559351	-3.393439	0.154566
H	-7.065862	-1.457642	1.846338	H	-7.015968	-1.458396	1.930737
H	-0.447231	1.229167	-2.409737	H	-0.390595	1.135304	-2.512132
H	0.828081	-3.972876	-0.922090	H	1.089841	-4.007102	-0.922821
H	-2.926034	4.702500	0.624567	H	-3.261524	4.386922	0.867315
H	-2.677193	1.192175	-1.833821	H	-2.578361	1.237589	-1.954120
H	-4.937111	5.414522	-0.605803	H	-5.360967	4.950703	-0.279479
H	-4.703432	1.892247	-3.031237	H	-4.686116	1.784776	-3.071571
H	-5.840241	4.004821	-2.428452	H	-6.087968	3.642320	-2.244550
H	0.594648	1.120959	-3.824368	H	0.689929	1.047615	-3.889992
H	0.266761	2.694215	-3.100101	H	0.298313	2.613478	-3.187692
H	4.035302	3.725810	-3.116257	H	4.066805	3.681482	-3.210662
H	2.317815	3.833930	-3.479490	H	2.351515	3.801420	-3.562305
H	3.306367	2.605736	-4.264847	H	3.316267	2.539818	-4.316928
H	-0.837580	4.483120	-1.662771	H	-1.074193	4.353780	-1.791111
H	1.320281	2.806770	1.640203	H	1.312492	2.993434	1.489500
H	1.006791	6.072264	-2.054974	H	0.649788	6.013265	-2.351401
H	3.183315	4.366252	1.213579	H	3.052579	4.630116	0.900334
H	3.026328	6.006307	-0.631744	H	2.721193	6.148779	-1.018032
H	-5.153353	2.162732	3.129212	H	-4.924125	1.919427	3.534592
H	-7.110847	0.673604	3.103554	H	-6.929228	0.511342	3.421237
H	2.227613	-5.025126	-1.072158	H	2.519620	-5.019429	-0.873085
H	1.385527	-4.920312	0.469435	H	1.536991	-4.841753	0.571492
H	6.495258	-2.637487	1.025749	H	6.565953	-2.365523	1.277268
H	5.841305	-1.361614	2.053828	H	5.821389	-1.089721	2.235567
H	6.236668	-1.019394	0.371360	H	6.271836	-0.783204	0.563396
H	4.511546	0.549939	1.171382	H	4.459234	0.734435	1.252220
H	-1.056409	-1.254533	3.030803	H	-0.817855	-1.609459	3.099171
H	-1.536788	0.439751	2.926209	H	-1.551733	-0.009462	3.066921
H	-0.983155	-0.223626	4.458433	H	-0.849074	-0.619589	4.552566
H	4.727921	0.756547	3.649616	H	4.621358	1.111391	3.706181
H	3.856374	1.959701	4.600843	H	3.624248	2.256482	4.597536

H	4.263915	2.287562	2.914804	H	4.016618	2.554426	2.905815
H	0.952915	0.325889	5.973560	H	1.044466	0.180381	6.031455
H	0.368163	1.889547	5.407967	H	0.204701	1.639257	5.518231
H	2.070921	1.668983	5.790610	H	1.929445	1.682012	5.840728
H	5.751402	1.375318	-0.961522	H	5.754959	1.516326	-0.874647
H	5.146831	2.597549	0.153797	H	5.081503	2.769385	0.158898
H	5.369742	2.991098	-1.553810	H	5.352135	3.088417	-1.553201
H	4.278359	-5.473290	0.922288	H	4.478266	-5.302146	1.120667
H	5.761841	-4.610623	0.532973	H	5.938009	-4.377277	0.810833
H	4.701526	-5.161820	-0.758127	H	4.984966	-4.984553	-0.532624
H	-3.110162	1.569028	1.889649	H	-2.952237	1.399621	2.153214

Table S6. Cartesian coordinates of the geometry optimized structures of ^{3}**IM1^{+}** in the gas phase; PBE (left) CAM-b3lyp (right), def2-TZVP/ECP(W, I).

C	-3.604881	-4.377046	-1.087788	C	-3.658476	-4.306682	-1.189903
C	-2.837339	-3.264727	-0.773064	C	-2.881553	-3.208230	-0.858281
C	-4.940159	-4.448587	-0.711927	C	-4.983652	-4.385556	-0.790769
C	-3.417211	-2.216211	-0.066040	C	-3.441454	-2.180362	-0.110386
C	-5.538600	-3.402317	-0.010579	C	-5.561906	-3.359893	-0.047727
C	-2.892095	-0.937859	0.376705	C	-2.902134	-0.917752	0.364674
C	-4.780182	-2.297581	0.309349	C	-4.794204	-2.270479	0.287528
C	-1.596544	-0.420588	0.189296	C	-1.599074	-0.402816	0.172841
C	-3.971958	-0.257968	1.074066	C	-3.968782	-0.256348	1.103583
C	-5.124878	-1.076380	1.027812	C	-5.122609	-1.067669	1.052024
W	0.454168	-0.769187	-0.002703	W	0.444872	-0.764205	-0.009142
C	-0.922080	0.736673	0.148464	C	-0.924883	0.742443	0.141580
C	-4.011850	0.946760	1.767348	C	-3.998139	0.926013	1.830777
C	-6.301049	-0.675674	1.623510	C	-6.286595	-0.686277	1.675873
I	0.191401	-1.906500	-2.483271	I	0.240574	-1.928165	-2.498793
N	1.712075	0.713904	-0.988315	N	1.718951	0.721281	-0.986024
N	1.261424	0.005128	1.838707	N	1.234489	-0.004476	1.861468
N	2.323141	-1.958305	0.177671	N	2.332541	-1.952547	0.207614
C	-0.332443	-2.351364	1.041628	C	-0.342915	-2.400313	1.016392
P	-1.199957	2.419477	0.516638	P	-1.215669	2.433298	0.537922
C	-2.637562	2.927545	-0.454220	C	-2.646766	2.913297	-0.466689
C	-3.231520	4.146532	-0.123080	C	-3.293339	4.096367	-0.116597
C	-3.205691	2.138062	-1.450124	C	-3.156614	2.146915	-1.507270
C	-4.365385	4.574190	-0.791883	C	-4.417441	4.511259	-0.804979
C	-4.350689	2.562605	-2.105433	C	-4.291857	2.555724	-2.185161
C	-4.929761	3.779889	-1.780702	C	-4.921562	3.738206	-1.838513
C	0.307945	1.560752	-2.884583	C	0.359214	1.550417	-2.926891
C	1.548476	1.476481	-2.082642	C	1.582942	1.465350	-2.093664
C	2.730477	2.171251	-2.344495	C	2.770366	2.150198	-2.342950
N	2.971977	0.922310	-0.540421	N	2.974620	0.935002	-0.514082
C	2.968902	3.104351	-3.475932	C	3.030563	3.066263	-3.488162
N	3.487686	-1.314741	0.426428	N	3.489373	-1.293338	0.477017

C	2.610519	-3.272836	0.099555	C	2.640923	-3.259942	0.128711
C	4.500804	-2.190536	0.494517	C	4.508438	-2.158254	0.555752
C	0.178296	3.476567	0.014339	C	0.159446	3.486001	-0.000547
C	0.082011	4.389469	-1.033350	C	0.065280	4.368549	-1.070126
C	1.310324	3.482903	0.828508	C	1.288752	3.522657	0.812170
C	1.118417	5.276499	-1.277939	C	1.094610	5.255032	-1.334730
C	2.351784	4.355536	0.565503	C	2.324153	4.393760	0.532665
C	2.257388	5.255620	-0.486617	C	2.228260	5.264271	-0.540547
C	-5.198562	1.344496	2.367572	C	-5.173502	1.306094	2.459423
C	-6.334371	0.549135	2.289373	C	-6.309059	0.515667	2.377846
O	-0.758430	-3.175763	1.691765	O	-0.770987	-3.236760	1.635534
N	2.541349	0.439536	1.878020	N	2.507280	0.463099	1.906467
C	0.704168	0.271245	3.037166	C	0.673091	0.239625	3.059518
C	3.980364	-3.461259	0.286452	C	4.007486	-3.431242	0.336494
C	1.632871	-4.363513	-0.129154	C	1.685666	-4.372273	-0.115435
C	5.901725	-1.776018	0.749758	C	5.904590	-1.729792	0.833544
B	3.488320	0.189771	0.691078	B	3.474645	0.214917	0.733133
C	2.802858	0.983160	3.075028	C	2.748494	1.004007	3.106457
C	1.647889	0.900077	3.849074	C	1.597130	0.885852	3.875592
C	-0.680127	-0.097034	3.414643	C	-0.705936	-0.163512	3.434834
C	4.128851	1.548971	3.422116	C	4.060269	1.605518	3.462343
C	1.445526	1.359922	5.249830	C	1.377301	1.334238	5.281503
C	3.608109	1.794405	-1.337983	C	3.625054	1.790120	-1.315776
C	5.005827	2.228282	-1.102219	C	5.021125	2.229483	-1.059890
C	4.697462	-4.763981	0.261214	C	4.741068	-4.729183	0.319044
H	-3.157143	-5.195403	-1.638447	H	-3.225301	-5.107507	-1.773189
H	-1.807738	-3.215230	-1.096287	H	-1.862566	-3.157010	-1.202177
H	-5.524290	-5.324158	-0.969106	H	-5.575193	-5.249377	-1.061867
H	-6.582275	-3.463890	0.275211	H	-6.597783	-3.425650	0.257308
H	-7.186416	-1.299705	1.585061	H	-7.171236	-1.307432	1.631078
H	-0.562506	1.209497	-2.337578	H	-0.524335	1.204857	-2.404587
H	0.734686	-4.025103	-0.638706	H	0.789373	-4.057375	-0.635848
H	-2.804186	4.761353	0.662355	H	-2.916099	4.695559	0.703100
H	-2.765449	1.183870	-1.711969	H	-2.681654	1.218860	-1.790835
H	-4.817298	5.524042	-0.532051	H	-4.908542	5.433666	-0.526419
H	-4.789169	1.939048	-2.875448	H	-4.683176	1.946745	-2.988732
H	-5.823944	4.109699	-2.296016	H	-5.807826	4.056400	-2.370390
H	0.390896	0.960759	-3.793952	H	0.464787	0.949350	-3.829767
H	0.137739	2.596019	-3.184198	H	0.199463	2.583121	-3.231461
H	3.854569	3.717855	-3.309068	H	3.937425	3.647858	-3.336330
H	2.123648	3.782784	-3.609209	H	2.210973	3.772423	-3.619755
H	3.110531	2.568943	-4.418900	H	3.143871	2.517763	-4.424805
H	-0.809196	4.414282	-1.648845	H	-0.819987	4.374394	-1.690366
H	1.364372	2.813591	1.679550	H	1.347492	2.877159	1.678112
H	1.034118	5.990099	-2.089352	H	1.007930	5.945086	-2.163356
H	3.229368	4.354903	1.201880	H	3.197965	4.414445	1.170480
H	3.063736	5.953711	-0.678549	H	3.029133	5.961274	-0.747543
H	-5.237480	2.286539	2.900803	H	-5.202686	2.230979	3.018728

H	-7.253347	0.878248	2.759622	H	-7.218188	0.830358	2.871842
H	2.090599	-5.137390	-0.747400	H	2.166479	-5.132894	-0.728507
H	1.345321	-4.833525	0.815864	H	1.399602	-4.849417	0.823913
H	6.561597	-2.640256	0.698226	H	6.571664	-2.586513	0.798926
H	6.012826	-1.325501	1.738356	H	5.993264	-1.273381	1.818993
H	6.242490	-1.045595	0.013837	H	6.250621	-1.003057	0.099869
H	4.585956	0.562357	0.965160	H	4.559331	0.595479	1.020606
H	-0.791455	-1.177954	3.527896	H	-0.803840	-1.247331	3.498782
H	-1.412514	0.241376	2.683616	H	-1.448381	0.201746	2.732203
H	-0.930099	0.357921	4.371915	H	-0.950793	0.241593	4.413012
H	4.893409	0.770537	3.471347	H	4.842938	0.848900	3.513129
H	4.084477	2.038093	4.393568	H	3.999048	2.091026	4.432203
H	4.456088	2.282835	2.683883	H	4.368544	2.348158	2.728199
H	1.287120	0.520027	5.931024	H	1.210781	0.489782	5.951458
H	0.578001	2.018699	5.332667	H	0.511661	1.992595	5.359534
H	2.309977	1.914612	5.613959	H	2.236336	1.883040	5.660490
H	5.708687	1.399932	-1.215944	H	5.721505	1.397269	-1.127101
H	5.135467	2.636111	-0.098281	H	5.124820	2.670557	-0.069591
H	5.280321	2.999309	-1.820210	H	5.316291	2.973618	-1.794426
H	4.330443	-5.439870	1.037824	H	4.356899	-5.416815	1.073724
H	5.767565	-4.633920	0.420889	H	5.801555	-4.589949	0.516115
H	4.570095	-5.271706	-0.698632	H	4.652256	-5.224816	-0.648968
H	-3.136969	1.577356	1.854835	H	-3.127042	1.554782	1.923745

Table S7. Cartesian coordinates of the geometry optimized structures of *syn*-**IM2**+ in the gas phase; PBE (left) CAM-b3lyp (right), def2-TZVP/ECP(W, I).

C	-4.18669	-4.678728	0.461957	C	-4.167562	-4.707014	0.468408
C	-3.17393	-3.732614	0.348714	C	-3.172663	-3.747238	0.339711
C	-5.41153	-4.358884	1.036202	C	-5.388545	-4.401851	1.052568
C	-3.40706	-2.456808	0.832127	C	-3.420908	-2.475188	0.816727
C	-5.66227	-3.073714	1.501239	C	-5.652388	-3.119969	1.513782
C	-2.58252	-1.247491	0.837161	C	-2.609004	-1.251244	0.810529
C	-4.66295	-2.123557	1.393817	C	-4.670142	-2.158559	1.390850
C	-1.28224	-0.914141	0.338204	C	-1.303703	-0.914890	0.304930
C	-3.32037	-0.230495	1.386908	C	-3.343108	-0.249270	1.362014
C	-4.63987	-0.704317	1.727733	C	-4.660953	-0.732635	1.723153
W	0.69891	-0.845769	-0.154290	W	0.688440	-0.856056	-0.171659
C	-0.91537	0.317894	-0.078994	C	-0.936538	0.306842	-0.093625
C	-2.91745	1.187929	1.576779	C	-2.943150	1.175049	1.556542
C	-5.58507	0.174040	2.132303	C	-5.602121	0.131334	2.133564
I	0.51762	-1.826333	-2.750006	I	0.523671	-1.808235	-2.802960
N	1.63906	1.010924	-1.023603	N	1.640033	1.029032	-1.007622
N	1.37544	-0.127677	1.800861	N	1.346314	-0.159177	1.816715
N	2.79876	-1.534489	-0.091267	N	2.806168	-1.528466	-0.099796
C	0.25242	-2.635099	0.575187	C	0.277661	-2.682791	0.523648
P	-1.98442	1.669634	0.057578	P	-1.993742	1.671628	0.061424

C	-3.13823	1.721133	-1.297763	C	-3.124408	1.772005	-1.311344
C	-4.11072	2.721164	-1.356258	C	-4.073804	2.791506	-1.364140
C	-3.13932	0.679419	-2.224281	C	-3.130735	0.760233	-2.266708
C	-5.06109	2.689733	-2.361552	C	-5.003975	2.808407	-2.384641
C	-4.09776	0.657078	-3.223032	C	-4.067963	0.784883	-3.282449
C	-5.05182	1.661343	-3.294593	C	-4.998608	1.807723	-3.344076
C	0.11146	1.729410	-2.887185	C	0.155208	1.765463	-2.901762
C	1.27143	1.874890	-1.980169	C	1.294458	1.899812	-1.961651
C	2.15931	2.957362	-2.007746	C	2.175644	2.985868	-1.956521
N	2.74809	1.522198	-0.446942	N	2.735569	1.541331	-0.396805
C	2.08772	4.118079	-2.933625	C	2.119336	4.156891	-2.875990
N	3.76766	-0.646990	0.245312	N	3.762944	-0.633988	0.272547
C	3.40321	-2.717227	-0.308394	C	3.430872	-2.692984	-0.340528
C	4.96823	-1.247236	0.229156	C	4.968258	-1.220789	0.252644
C	-1.06765	3.176150	0.316597	C	-1.057529	3.160434	0.359246
C	-1.24412	4.304641	-0.478697	C	-1.187030	4.294291	-0.433040
C	-0.08079	3.155306	1.304871	C	-0.099359	3.119971	1.371762
C	-0.44061	5.415307	-0.275537	C	-0.369902	5.387066	-0.204251
C	0.71492	4.268953	1.497354	C	0.711479	4.214279	1.590717
C	0.53553	5.397955	0.708950	C	0.576573	5.347735	0.804182
C	-4.04147	2.072815	1.987661	C	-4.082875	2.050632	1.967248
C	-5.25989	1.577528	2.256504	C	-5.286418	1.546611	2.248819
O	-0.02177	-3.655122	1.019402	O	0.021459	-3.710774	0.943674
N	2.45736	0.676830	1.881139	N	2.422222	0.656726	1.918988
C	0.84824	-0.209951	3.034648	C	0.815442	-0.267663	3.043391
C	4.77996	-2.575478	-0.125535	C	4.801058	-2.538336	-0.137292
C	2.71810	-3.981365	-0.664594	C	2.772328	-3.964610	-0.734664
C	6.22773	-0.530670	0.546574	C	6.219912	-0.498430	0.602744
B	3.42510	0.777198	0.688528	B	3.403123	0.782025	0.736775
C	2.61734	1.119727	3.137349	C	2.565621	1.077081	3.183282
C	1.59130	0.589158	3.910120	C	1.542697	0.522429	3.936905
C	-0.31653	-1.048678	3.405907	C	-0.350335	-1.119238	3.397087
C	3.74559	1.994698	3.541440	C	3.681171	1.961268	3.614109
C	1.34664	0.775454	5.366891	C	1.282236	0.679011	5.398043
C	3.08678	2.691973	-1.015645	C	3.078278	2.716983	-0.947616
C	4.26521	3.485190	-0.587164	C	4.242513	3.517199	-0.483419
C	5.80030	-3.646110	-0.284705	C	5.837993	-3.595680	-0.312121
H	-4.01733	-5.683475	0.093566	H	-3.987427	-5.709461	0.104850
H	-2.23089	-3.995102	-0.111201	H	-2.232067	-3.998141	-0.127106
H	-6.18096	-5.117296	1.114124	H	-6.144773	-5.169405	1.142309
H	-6.62423	-2.826823	1.936029	H	-6.611287	-2.885657	1.957286
H	-6.60021	-0.146132	2.337081	H	-6.610150	-0.194463	2.353370
H	-0.49379	0.865172	-2.642262	H	-0.464065	0.909519	-2.681075
H	1.79917	-3.815307	-1.221816	H	1.854907	-3.807756	-1.290009
H	-4.13554	3.513849	-0.617836	H	-4.096524	3.566183	-0.610080
H	-2.39790	-0.109904	-2.168073	H	-2.410103	-0.044698	-2.224035
H	-5.81475	3.465938	-2.413600	H	-5.738394	3.600522	-2.428975
H	-4.09742	-0.150041	-3.945109	H	-4.068715	-0.000042	-4.025637

H	-5.79877	1.640588	-4.079256	H	-5.729207	1.823950	-4.141376
H	0.44978	1.605774	-3.918985	H	0.520955	1.642126	-3.921610
H	-0.50735	2.630803	-2.857284	H	-0.453765	2.670960	-2.885096
H	2.85605	4.858394	-2.709551	H	2.868960	4.903119	-2.620606
H	1.11880	4.621334	-2.866893	H	1.144709	4.646939	-2.836919
H	2.22081	3.812493	-3.975140	H	2.290704	3.863141	-3.913044
H	-1.98788	4.311475	-1.265639	H	-1.905897	4.322716	-1.238758
H	0.09038	2.264796	1.900275	H	0.039825	2.228936	1.969659
H	-0.57380	6.294129	-0.894636	H	-0.469138	6.268510	-0.822347
H	1.48398	4.250224	2.259972	H	1.456989	4.177639	2.372816
H	1.16485	6.267122	0.859095	H	1.217119	6.202267	0.975037
H	-3.83045	3.126984	2.127493	H	-3.885186	3.106487	2.097846
H	-6.03943	2.252952	2.589629	H	-6.069761	2.212990	2.584398
H	3.37978	-4.590742	-1.281441	H	3.447491	-4.540763	-1.364776
H	2.48175	-4.561363	0.231892	H	2.549283	-4.571599	0.144527
H	7.07826	-1.196321	0.409868	H	7.076998	-1.151608	0.464649
H	6.23945	-0.176175	1.579398	H	6.213185	-0.165976	1.640368
H	6.36832	0.335901	-0.101984	H	6.360461	0.380082	-0.025539
H	4.41456	1.349986	1.025960	H	4.379657	1.352912	1.093087
H	-0.23548	-2.056010	3.000150	H	-0.310507	-2.088696	2.909906
H	-1.26658	-0.639394	3.059104	H	-1.302034	-0.661323	3.132691
H	-0.37173	-1.127265	4.491399	H	-0.360078	-1.286303	4.471847
H	4.69218	1.449148	3.543099	H	4.632479	1.428995	3.612471
H	3.58083	2.377941	4.547533	H	3.504209	2.323203	4.623642
H	3.86152	2.842824	2.865046	H	3.789275	2.821527	2.955739
H	1.53606	-0.143413	5.928649	H	1.475151	-0.246770	5.942794
H	0.31569	1.074502	5.570025	H	0.248365	0.963639	5.596010
H	1.99613	1.546142	5.782291	H	1.918456	1.446718	5.833361
H	5.19330	2.920740	-0.695573	H	5.176218	2.965700	-0.586555
H	4.18825	3.791341	0.458706	H	4.143137	3.801008	0.564281
H	4.34787	4.385024	-1.195056	H	4.325670	4.427586	-1.071249
H	5.62807	-4.472099	0.410039	H	5.650142	-4.451966	0.336982
H	6.80637	-3.269397	-0.100934	H	6.832042	-3.221672	-0.077268
H	5.78772	-4.063075	-1.295223	H	5.862984	-3.964423	-1.338730
H	-2.12350	1.260545	2.343555	H	-2.173437	1.235594	2.343037

Table S8. Cartesian coordinates of the geometry optimized structures of *anti*-**IM2**$^+$ in the gas phase; PBE (left) CAM-b3lyp (right), def2-TZVP/ECP(W, I).

C	-3.491591	-5.317079	-0.157462	C	-3.519910	-5.283796	-0.128509
C	-2.694539	-4.202032	-0.396182	C	-2.722067	-4.171306	-0.363018
C	-4.796626	-5.190339	0.299656	C	-4.833092	-5.153356	0.295321
C	-3.218222	-2.942139	-0.161266	C	-3.253838	-2.912737	-0.158037
C	-5.347947	-3.931932	0.500151	C	-5.389334	-3.895332	0.469358
C	-2.673506	-1.583088	-0.342047	C	-2.703634	-1.550466	-0.339613
C	-4.564140	-2.818642	0.257947	C	-4.603455	-2.786369	0.232151
C	-1.358789	-1.032522	-0.500529	C	-1.374688	-1.011148	-0.498980

C	-3.709265	-0.703111	-0.142237	C	-3.728775	-0.672748	-0.159852
C	-4.890327	-1.401639	0.300113	C	-4.929306	-1.363459	0.263036
W	0.689360	-0.760714	-0.440967	W	0.681514	-0.768861	-0.440939
C	-1.027648	0.275368	-0.572780	C	-1.034535	0.281221	-0.559499
C	-3.754030	0.758873	-0.381853	C	-3.758675	0.793070	-0.404883
C	-5.978831	-0.709918	0.712422	C	-6.012184	-0.670191	0.652910
I	1.251342	-1.244860	-3.114096	I	1.223910	-1.243590	-3.145834
N	1.659349	1.254052	-0.686498	N	1.668618	1.259465	-0.668050
N	0.779948	-0.389048	1.721427	N	0.781414	-0.423168	1.741853
N	2.777820	-1.356703	0.028629	N	2.786783	-1.363303	0.018947
C	0.363673	-2.675609	-0.091957	C	0.385608	-2.707777	-0.112797
P	-2.119356	1.565228	-0.196580	P	-2.122442	1.582562	-0.193796
C	-2.163297	2.954552	-1.324740	C	-2.130664	2.975150	-1.318719
C	-1.914831	4.251330	-0.884435	C	-1.883738	4.268429	-0.874940
C	-2.488118	2.721380	-2.662459	C	-2.434160	2.749406	-2.660577
C	-1.986511	5.306667	-1.781137	C	-1.935390	5.324635	-1.768556
C	-2.567948	3.779950	-3.547747	C	-2.493749	3.807364	-3.543699
C	-2.314499	5.072955	-3.107636	C	-2.242092	5.096249	-3.098066
C	0.718729	2.206687	-2.811570	C	0.781371	2.231982	-2.809657
C	1.487781	2.266187	-1.549091	C	1.527931	2.273062	-1.529270
C	2.201152	3.388659	-1.106047	C	2.244886	3.385545	-1.072577
N	2.487607	1.702289	0.282459	N	2.486663	1.698619	0.320116
C	2.271760	4.707426	-1.790295	C	2.342003	4.706130	-1.757973
N	3.558522	-0.501152	0.734256	N	3.561218	-0.510851	0.745490
C	3.540566	-2.424637	-0.275731	C	3.561801	-2.413404	-0.304019
C	4.795556	-1.004128	0.867689	C	4.800549	-1.006921	0.866830
C	-1.754356	2.130396	1.454414	C	-1.769271	2.127857	1.467165
C	-0.500850	2.701844	1.684671	C	-0.520778	2.696120	1.717687
C	-2.655890	1.956006	2.501601	C	-2.674511	1.939456	2.505246
C	-0.171198	3.120714	2.959824	C	-0.200521	3.097694	2.997634
C	-2.308721	2.373825	3.776429	C	-2.338057	2.338582	3.786797
C	-1.072544	2.959032	4.003512	C	-1.107403	2.921391	4.030999
C	-4.947904	1.444252	0.193547	C	-4.961138	1.482621	0.161494
C	-5.980586	0.733443	0.673596	C	-6.001468	0.781507	0.617716
O	0.227121	-3.774920	0.199822	O	0.266814	-3.808535	0.156072
N	1.793254	0.331428	2.251953	N	1.791754	0.303074	2.278237
C	-0.010451	-0.765870	2.741902	C	-0.000970	-0.813976	2.758844
C	4.827951	-2.235638	0.231058	C	4.844422	-2.222266	0.207746
C	3.101921	-3.628701	-1.019001	C	3.140775	-3.616425	-1.066909
C	5.878569	-0.290130	1.587453	C	5.881725	-0.298632	1.602060
B	2.973008	0.756462	1.374418	B	2.971383	0.739420	1.402806
C	1.663108	0.412210	3.585192	C	1.658360	0.371258	3.610162
C	0.506008	-0.264824	3.940526	C	0.510532	-0.318091	3.959407
C	-1.245390	-1.572935	2.615270	C	-1.231130	-1.635088	2.628987
C	2.648253	1.109834	4.447693	C	2.635285	1.076989	4.481133
C	-0.080016	-0.441462	5.296293	C	-0.077251	-0.509794	5.316359
C	2.833472	2.981756	0.058400	C	2.849189	2.972918	0.099921
C	3.752937	3.735808	0.946677	C	3.760904	3.720830	1.006871

C	5.968779	-3.180190	0.095433	C	5.995499	-3.157458	0.054496
H	-3.084514	-6.304132	-0.341952	H	-3.107523	-6.270521	-0.289653
H	-1.695044	-4.335920	-0.777934	H	-1.715901	-4.308244	-0.718510
H	-5.393937	-6.076106	0.477872	H	-5.432106	-6.036227	0.470359
H	-6.378338	-3.829101	0.821357	H	-6.424248	-3.790945	0.768056
H	-6.853237	-1.222052	1.097558	H	-6.897084	-1.173244	1.019910
H	-0.032643	1.422921	-2.801498	H	0.019000	1.464321	-2.823074
H	2.267726	-3.431900	-1.686668	H	2.298744	-3.431452	-1.721883
H	-1.671540	4.439966	0.153243	H	-1.656528	4.458390	0.163335
H	-2.649104	1.712304	-3.027636	H	-2.597049	1.745371	-3.031423
H	-1.792667	6.315455	-1.437406	H	-1.742388	6.329629	-1.419771
H	-2.815925	3.595168	-4.585670	H	-2.725013	3.625799	-4.583935
H	-2.373502	5.900767	-3.804164	H	-2.285538	5.924256	-3.792404
H	1.388377	1.996523	-3.648936	H	1.463788	2.015767	-3.631636
H	0.241581	3.169630	-2.998850	H	0.325783	3.201707	-3.000298
H	2.788565	5.448389	-1.179821	H	2.885728	5.430323	-1.154716
H	1.273132	5.097483	-2.006501	H	1.354025	5.123595	-1.960277
H	2.803922	4.639816	-2.743096	H	2.859595	4.624874	-2.715159
H	0.212173	2.811920	0.875195	H	0.199205	2.817473	0.919863
H	-3.624092	1.503005	2.326040	H	-3.639375	1.489352	2.321614
H	0.797239	3.570452	3.140809	H	0.764782	3.543777	3.191472
H	-3.008908	2.244229	4.592730	H	-3.042550	2.195585	4.594357
H	-0.806597	3.286320	5.001693	H	-0.849587	3.234747	5.033585
H	-4.993521	2.525683	0.136375	H	-4.997788	2.562857	0.115179
H	-6.858715	1.255805	1.034981	H	-6.880132	1.306238	0.967907
H	3.931574	-4.001254	-1.621688	H	3.970123	-3.957839	-1.684278
H	2.815743	-4.426015	-0.327384	H	2.883443	-4.429516	-0.385836
H	6.808795	-0.850186	1.508356	H	6.813473	-0.850519	1.514709
H	5.646335	-0.170241	2.647806	H	5.647120	-0.199202	2.661501
H	6.046295	0.704918	1.171419	H	6.043827	0.701966	1.203590
H	3.797936	1.298452	2.042891	H	3.791367	1.271594	2.074365
H	-1.128421	-2.412485	1.933178	H	-1.109571	-2.462723	1.937616
H	-2.086959	-0.973473	2.261056	H	-2.079352	-1.040721	2.291162
H	-1.517789	-1.970381	3.592808	H	-1.491927	-2.049671	3.600039
H	3.598264	0.571797	4.486097	H	3.593249	0.557752	4.507557
H	2.267076	1.184071	5.465238	H	2.258071	1.130471	5.499050
H	2.860777	2.117844	4.087996	H	2.825034	2.091625	4.134513
H	-0.116382	-1.494487	5.587906	H	-0.100971	-1.562537	5.601838
H	-1.101096	-0.053852	5.345087	H	-1.100899	-0.135334	5.365185
H	0.504791	0.081676	6.053163	H	0.498267	0.015600	6.075639
H	4.761712	3.317594	0.926258	H	4.764613	3.296354	1.001771
H	3.417773	3.724046	1.985615	H	3.407680	3.708053	2.037303
H	3.815644	4.773663	0.622617	H	3.835514	4.757249	0.688245
H	5.747193	-4.146255	0.556187	H	5.771362	-4.139634	0.472907
H	6.868946	-2.790392	0.570495	H	6.883230	-2.783447	0.559769
H	6.208898	-3.368789	-0.954379	H	6.254654	-3.302891	-0.995422
H	-3.858328	0.874581	-1.478956	H	-3.855684	0.899479	-1.498313

Table S9. Cartesian coordinates of the geometry optimized structures of **4c**$^{+}$ in the gas phase; PBE (left) CAM-b3lyp (right), def2-TZVP/ECP(W, I).

C	-3.495830	-5.210900	-0.174243	C	-3.453385	-5.221432	-0.172357
C	-2.899891	-4.036257	-0.616401	C	-2.875025	-4.041942	-0.618274
C	-4.388782	-5.201269	0.891715	C	-4.350874	-5.217899	0.886374
C	-3.191907	-2.857630	0.041086	C	-3.190327	-2.865373	0.026878
C	-4.713190	-4.015229	1.532233	C	-4.695156	-4.033633	1.516384
C	-2.763224	-1.447568	-0.281567	C	-2.776959	-1.449553	-0.302526
C	-4.113953	-2.841831	1.098958	C	-4.113291	-2.857090	1.078875
C	-1.363061	-0.949363	-0.379789	C	-1.375424	-0.948043	-0.400115
C	-3.474533	-0.639332	0.785622	C	-3.493839	-0.647381	0.769276
C	-4.275164	-1.468103	1.570180	C	-4.288920	-1.479087	1.549193
W	0.664119	-0.702279	-0.466919	W	0.659499	-0.708452	-0.475826
C	-1.036238	0.349639	-0.367679	C	-1.048679	0.338967	-0.371564
C	-3.309652	0.702205	1.092493	C	-3.330778	0.691285	1.079922
C	-4.978478	-0.923308	2.637734	C	-4.994685	-0.942976	2.614540
I	0.959746	-0.841108	-3.237134	I	0.957075	-0.836575	-3.268340
N	1.613299	1.345250	-0.559397	N	1.608019	1.357105	-0.552460
N	0.963068	-0.602402	1.697112	N	0.951259	-0.618968	1.708560
N	2.773858	-1.344845	-0.264627	N	2.787594	-1.330517	-0.264932
C	0.287566	-2.645415	-0.394994	C	0.325676	-2.676525	-0.415167
P	-2.162991	1.599046	0.044796	P	-2.174249	1.591839	0.045701
C	-3.061276	2.137587	-1.403762	C	-3.060379	2.152272	-1.401742
C	-4.065021	3.098199	-1.274457	C	-4.059170	3.114148	-1.264957
C	-2.832625	1.516592	-2.629602	C	-2.830223	1.554238	-2.635618
C	-4.813610	3.456590	-2.381300	C	-4.799780	3.494250	-2.366380
C	-3.593638	1.877374	-3.729692	C	-3.581769	1.936590	-3.732325
C	-4.576033	2.848497	-3.607465	C	-4.558982	2.907462	-3.599782
C	0.387465	2.586833	-2.369166	C	0.416766	2.605827	-2.384417
C	1.365318	2.456234	-1.267556	C	1.374274	2.466795	-1.261498
C	2.180380	3.500480	-0.814754	C	2.177089	3.510929	-0.792366
N	2.578848	1.664470	0.329821	N	2.558937	1.677860	0.358486
C	2.202593	4.884441	-1.356309	C	2.202855	4.898922	-1.332901
N	3.612036	-0.597865	0.496990	N	3.611285	-0.578834	0.517013
C	3.490005	-2.383415	-0.736747	C	3.524654	-2.347704	-0.743509
C	4.839510	-1.140117	0.500839	C	4.844027	-1.104929	0.520207
C	-1.364960	2.933178	0.915812	C	-1.369626	2.911296	0.933772
C	-1.387027	4.246610	0.457399	C	-1.373858	4.225901	0.485717
C	-0.612352	2.586185	2.039427	C	-0.631796	2.555597	2.061765
C	-0.660340	5.215708	1.130346	C	-0.647641	5.184171	1.170028
C	0.113462	3.560321	2.698742	C	0.093675	3.517320	2.733742
C	0.089635	4.872802	2.245062	C	0.085792	4.830444	2.288751
C	-4.002393	1.244092	2.173769	C	-4.027389	1.225017	2.159740
C	-4.841777	0.430194	2.924316	C	-4.865235	0.408736	2.904512
O	0.074844	-3.767811	-0.322450	O	0.135879	-3.797084	-0.350589
N	1.972295	0.140925	2.203762	N	1.952245	0.135696	2.223840
C	0.251663	-1.065055	2.739789	C	0.245680	-1.093999	2.745147

C	4.805814	-2.285319	-0.281715	C	4.832711	-2.236842	-0.276155
C	2.973014	-3.476463	-1.592664	C	3.035787	-3.445520	-1.616577
C	5.978544	-0.543810	1.240897	C	5.973594	-0.503718	1.277637
B	3.109461	0.613243	1.286042	B	3.091152	0.623704	1.313005
C	1.904921	0.169990	3.543514	C	1.876268	0.155555	3.561841
C	0.800177	-0.576434	3.930109	C	0.782666	-0.605970	3.938546
C	-0.931284	-1.950026	2.653084	C	-0.928571	-1.996357	2.653365
C	2.903029	0.879016	4.381726	C	2.860328	0.876827	4.412099
C	0.299300	-0.850059	5.304223	C	0.277709	-0.896327	5.311605
C	2.940472	2.952261	0.203080	C	2.917152	2.965799	0.236921
C	3.981521	3.584752	1.050143	C	3.940214	3.605203	1.106196
C	5.912178	-3.229800	-0.592037	C	5.956408	-3.165057	-0.591010
H	-3.264300	-6.145856	-0.669987	H	-3.204593	-6.154373	-0.659091
H	-2.216347	-4.054340	-1.455570	H	-2.187774	-4.055202	-1.452071
H	-4.843984	-6.129150	1.216548	H	-4.792855	-6.148446	1.215151
H	-5.427432	-4.009214	2.347493	H	-5.410295	-4.033675	2.328316
H	-3.205112	-1.187363	-1.258874	H	-3.221940	-1.198452	-1.276061
H	-5.607169	-1.546732	3.262824	H	-5.619845	-1.570051	3.236107
H	-0.161168	1.668683	-2.536111	H	-0.149320	1.703832	-2.555387
H	2.123915	-3.170215	-2.198229	H	2.181601	-3.158888	-2.217953
H	-4.269828	3.557583	-0.313578	H	-4.267115	3.560732	-0.301147
H	-2.062827	0.758606	-2.729078	H	-2.067606	0.795556	-2.748578
H	-5.589932	4.205947	-2.286292	H	-5.571747	4.243977	-2.262454
H	-3.414261	1.397879	-4.684168	H	-3.398836	1.473330	-4.691811
H	-5.166701	3.130089	-4.471024	H	-5.142628	3.205750	-4.460005
H	0.900097	2.832165	-3.302639	H	0.951631	2.828905	-3.308197
H	-0.312301	3.401867	-2.163155	H	-0.265089	3.437400	-2.198563
H	2.842202	5.537930	-0.762475	H	2.845663	5.547531	-0.741152
H	1.201586	5.324930	-1.364372	H	1.205762	5.343161	-1.336821
H	2.572926	4.910021	-2.385118	H	2.570069	4.924252	-2.360423
H	-1.954531	4.510085	-0.426670	H	-1.926927	4.502441	-0.400274
H	-0.577988	1.558623	2.385338	H	-0.607498	1.529471	2.404367
H	-0.674723	6.239803	0.777861	H	-0.649576	6.207961	0.822773
H	0.701746	3.291828	3.567783	H	0.669319	3.239436	3.605640
H	0.661354	5.633362	2.763658	H	0.657026	5.581861	2.816917
H	-3.872978	2.286430	2.441170	H	-3.903605	2.264347	2.432980
H	-5.378350	0.855043	3.764035	H	-5.404772	0.828633	3.742139
H	3.762741	-3.810220	-2.267233	H	3.832070	-3.747044	-2.295033
H	2.676566	-4.336836	-0.986216	H	2.766769	-4.319378	-1.020624
H	6.883179	-1.121093	1.057663	H	6.883856	-1.068921	1.097945
H	5.797130	-0.533318	2.317634	H	5.781888	-0.505176	2.350175
H	6.164110	0.484963	0.926947	H	6.151222	0.527215	0.974444
H	3.993319	1.064808	1.946092	H	3.962675	1.076214	1.977782
H	-0.933566	-2.562657	1.756173	H	-0.935455	-2.587579	1.745835
H	-1.859706	-1.374831	2.677175	H	-1.861438	-1.435425	2.704446
H	-0.948983	-2.617559	3.516023	H	-0.924433	-2.683389	3.498205
H	3.870317	0.371527	4.364967	H	3.833371	0.385841	4.396646
H	2.564117	0.915945	5.416175	H	2.516450	0.902256	5.442945

H	3.067237	1.901051	4.036829	H	3.008107	1.901382	4.074727
H	0.396938	-1.907806	5.564708	H	0.401266	-1.949424	5.570701
H	-0.756550	-0.587323	5.407656	H	-0.783310	-0.661489	5.406730
H	0.853312	-0.281312	6.051142	H	0.809083	-0.315083	6.062247
H	4.950040	3.094388	0.934307	H	4.911707	3.122635	1.006367
H	3.718859	3.545308	2.109690	H	3.657771	3.560085	2.157920
H	4.101166	4.630843	0.771713	H	4.057334	4.650986	0.834303
H	5.686146	-4.241254	-0.244969	H	5.730244	-4.186483	-0.282081
H	6.844432	-2.922613	-0.118364	H	6.872661	-2.867788	-0.085777
H	6.096978	-3.289221	-1.667985	H	6.167324	-3.187996	-1.661355

Table S10. Cartesian coordinates of the geometry optimized structures of **5** in the gas phase; PBE (left) CAM-b3lyp (right), def2-TZVP/ECP(W, I).

W	0.239651	10.508002	3.418770	W	0.253243	10.535992	3.344187
I	1.356124	10.487750	0.883444	I	1.452508	10.506928	0.816749
N	2.109435	11.520092	4.091288	N	2.123160	11.533502	4.086219
N	-0.026900	10.552574	5.597673	N	-0.073332	10.627899	5.527556
N	1.435812	8.638387	3.813476	N	1.442201	8.645972	3.789035
C	-1.366884	9.318163	3.319453	C	-1.339903	9.325908	3.256110
C	-1.759371	10.528329	2.817413	C	-1.749402	10.512397	2.737354
C	-0.437225	12.331871	3.250505	C	-0.380920	12.385804	3.118310
P	-2.503668	8.103401	3.772837	P	-2.444970	8.103052	3.761990
C	-3.011074	10.942891	2.423965	C	-3.014954	10.909881	2.375273
O	-0.852222	13.408866	3.242785	O	-0.760144	13.467495	3.065863
H	1.667219	13.088130	1.907430	H	1.775296	13.136703	1.904469
N	2.868166	10.948220	5.054622	N	2.844670	10.930492	5.065706
C	2.726608	12.650195	3.714096	C	2.791105	12.634942	3.722570
N	0.963400	10.148057	6.421899	N	0.874433	10.197555	6.397709
C	-1.082387	10.862619	6.361450	C	-1.152886	10.960499	6.240395
N	2.172160	8.537963	4.940120	N	2.114568	8.539455	4.958838
C	1.508531	7.458739	3.184707	C	1.554867	7.472936	3.160461
C	-4.086601	8.822447	3.397852	C	-4.041528	8.812853	3.439533
C	-2.354980	6.554285	2.857256	C	-2.313415	6.549361	2.845610
C	-2.285134	7.672985	5.511583	C	-2.168335	7.665281	5.493837
C	-3.590599	12.093564	1.756574	C	-3.627796	12.069591	1.748694
C	-4.095298	10.076596	2.766742	C	-4.080874	10.050145	2.779413
C	2.229999	13.596797	2.688364	C	2.338856	13.612289	2.700262
C	3.962131	11.693195	5.280220	C	3.966795	11.627027	5.296911
B	2.330984	9.754914	5.854247	B	2.252657	9.763484	5.871136
C	3.910165	12.796801	4.444136	C	3.967712	12.732775	4.468011
C	0.555921	10.209425	7.702692	C	0.397552	10.268527	7.653861
C	-0.760728	10.639984	7.707114	C	-0.904160	10.721506	7.593313
C	-2.376978	11.356419	5.837876	C	-2.414448	11.503677	5.675867
C	2.705130	7.306101	5.044382	C	2.647746	7.310135	5.079188
C	2.300308	6.578014	3.937682	C	2.311385	6.591369	3.947326
C	0.871498	7.142813	1.883716	C	0.973198	7.153075	1.829686

C	-5.306656	8.168261	3.613079	C	-5.252856	8.178103	3.740501
C	-2.629541	6.590176	1.490392	C	-2.669972	6.564802	1.499797
C	-2.009181	5.352579	3.465039	C	-1.911345	5.359290	3.435642
C	-3.351958	7.668186	6.404185	C	-3.194817	7.648084	6.428966
C	-0.998444	7.369621	5.956822	C	-0.873613	7.340519	5.887303
C	-3.024417	13.183762	1.095733	C	-3.097915	13.173557	1.084233
C	-5.004885	11.943582	1.755104	C	-5.035618	11.923374	1.821057
C	-5.328796	10.681177	2.391550	C	-5.329670	10.655440	2.469597
H	3.074258	14.109075	2.223677	H	3.202444	14.103653	2.253480
H	1.585129	14.354443	3.141288	H	1.715006	14.381124	3.158415
C	5.004698	11.324638	6.267755	C	4.996399	11.204293	6.280851
H	3.076454	9.494454	6.750212	H	2.963786	9.497609	6.784149
C	4.880303	13.918492	4.339266	C	4.975149	13.828940	4.399198
C	1.446089	9.882476	8.844386	C	1.173267	9.922326	8.871587
C	-1.673416	10.811212	8.869102	C	-1.867507	10.885972	8.718598
H	-2.247019	12.072260	5.028357	H	-2.235422	12.231804	4.891315
H	-2.934290	11.841389	6.639946	H	-2.979735	11.993417	6.466449
H	-2.995744	10.543823	5.447438	H	-3.043824	10.722277	5.250955
C	3.549034	6.8804870	6.189170	C	3.430679	6.881062	6.270354
C	2.629839	5.172349	3.576716	C	2.666954	5.187199	3.587349
H	1.608355	7.202329	1.079515	H	1.728338	7.258872	1.051731
H	0.075569	7.842261	1.645264	H	0.155949	7.817737	1.577512
H	0.465654	6.128333	1.897801	H	0.611309	6.124567	1.813841
C	-6.501716	8.771743	3.257159	C	-6.459583	8.775005	3.434560
H	-5.321664	7.171989	4.041858	H	-5.251846	7.198824	4.201827
H	-2.913056	7.526254	1.020371	H	-2.996489	7.487818	1.036926
C	-2.549858	5.430228	0.740685	C	-2.627910	5.399362	0.760067
C	-1.928816	4.193414	2.707561	C	-1.859367	4.195731	2.687397
H	-1.804717	5.318028	4.527861	H	-1.642048	5.330519	4.480722
H	-4.346204	7.931051	6.063503	H	-4.199330	7.921853	6.140168
C	-3.133083	7.345565	7.734817	C	-2.926612	7.294476	7.741113
C	-0.787634	7.057085	7.287976	C	-0.609885	7.000952	7.199019
H	-0.165029	7.391651	5.265271	H	-0.070343	7.358859	5.168025
H	-1.951130	13.300203	1.030556	H	-2.031920	13.296760	0.966948
C	-3.855299	14.119965	0.505126	C	-3.956778	14.120132	0.559860
C	-5.824292	12.896560	1.163054	C	-5.884431	12.887325	1.299335
C	-6.517622	10.028240	2.645523	C	-6.502122	10.017403	2.795058
H	5.260293	10.266337	6.200979	H	5.181391	10.133286	6.220601
H	4.681792	11.530647	7.292241	H	4.699809	11.443957	7.303606
H	5.908644	11.902265	6.077930	H	5.932098	11.716597	6.071035
H	5.234223	14.054828	3.313586	H	5.369455	13.960708	3.390062
H	5.754305	13.744683	4.968331	H	5.818913	13.629144	5.057607
H	4.431898	14.864521	4.655572	H	4.540050	14.781546	4.707045
H	1.687712	8.816666	8.892358	H	1.089037	8.858543	9.105108
H	0.961704	10.164131	9.779083	H	0.781680	10.472836	9.723810
H	2.391266	10.422966	8.778196	H	2.226483	10.171559	8.765619
H	-2.001390	11.847782	8.990700	H	-2.182236	11.922424	8.856280
H	-1.189515	10.507104	9.798390	H	-1.430226	10.543766	9.655545

H	-2.571141	10.198323	8.753981	H	-2.767351	10.294390	8.546278
H	4.310759	7.622356	6.430184	H	4.211039	7.596195	6.521488
H	4.051829	5.943710	5.949991	H	3.904813	5.921612	6.075762
H	2.953522	6.720993	7.092818	H	2.796714	6.772152	7.151449
H	1.734822	4.624460	3.268619	H	1.785908	4.625138	3.271464
H	3.077122	4.635067	4.414174	H	3.120180	4.657484	4.423435
H	3.334323	5.121381	2.741089	H	3.376010	5.148751	2.757983
H	-7.434173	8.252556	3.443452	H	-7.380704	8.268865	3.689325
H	-2.775135	5.458146	-0.318818	H	-2.926673	5.412225	-0.279365
C	-2.200228	4.230777	1.348414	C	-2.222275	4.213484	1.352703
H	-1.664308	3.257732	3.185848	H	-1.548305	3.272131	3.156628
H	-3.965336	7.335868	8.428869	H	-3.730573	7.271702	8.464237
C	-1.853623	7.043416	8.177098	C	-1.636294	6.976867	8.128725
H	0.213495	6.827794	7.633564	H	0.400656	6.755635	7.496289
H	-3.414804	14.967988	-0.007099	H	-3.543160	14.978296	0.046608
C	-5.245139	13.991887	0.545096	C	-5.340478	13.992010	0.671292
H	-6.902924	12.777880	1.174356	H	-6.958354	12.772123	1.383437
H	-7.463939	10.476831	2.361527	H	-7.457249	10.466758	2.552054
H	-5.869182	14.742369	0.074266	H	-5.986052	14.752595	0.253601
H	-2.146759	3.322402	0.759813	H	-2.200172	3.300685	0.772678
H	-1.685321	6.799127	9.219726	H	-1.428465	6.707894	9.156063

8. References

[S1] T. Spałek, P. Pietrzyk, Z. Sojka, *J. Chem. Inf. Model.* **2005**, *45*, 18.

[S2] a) K. Helmdach, S. Ludwig, A. Villinger, D. Hollmann, J. Kösters, W. W. Seidel, *Chem. Commun.* **2017**, *53*, 5894; b) C. J. Adams, I. M. Bartlett, S. Carlton, N. G. Connelly, D. J. Harding, O. D. Hayward, A. G. Orpen, E. Patron, C. D. Ray, P. H. Rieger, *Dalton Trans.* **2007**, 62; c) I. M. Bartlett, S. Carlton, N. G. Connelly, D. J. Harding, O. D. Hayward, A. Guy Orpen, C. D. Ray, P. H. Rieger, *Chem. Commun.* **1999**, 2403.

[S3] a) M. Krejčik, M. Daněk, F. Hartl, *J. Electroanal. Chem.* **1991**, *317*, 179; b) S. Oßwald, S. Breimaier, M. Linseis, R. F. Winter, *Organometallics* **2017**, *36*, 1993.

[S4] G. M. Sheldrick, *SHELXS-97: Program for the Solution of Crystal Structures*, University of Göttingen, Germany, **1997**.

[S5] G. M.Sheldrick, *SHELXL-97: Program for the Refinement of Crystal Structures*, University of Göttingen, Germany, **1997**.

[S6] H. Schröder, M. Sawall, C. Kubis, D. Selent, D. Hess, R. Franke, A. Börner, K. Neymeyr, *Anal. Chim. Acta* **2016**, *927*, 21.

[S7] M. Sawall, C. Kubis, E. Barsch, D. Selent, A. Börner, K. Neymeyr, *J. Iran. Chem. Soc.* **2016**, *13*, 191.

[S8] D. S. Frohnapfel, S. Reinartz, P. S. White, J. L. Templeton, *Organometallics* **1998**, *17*, 3759.

[S9] M. J. Frisch, G. W. Trucks, H. B. Schlegel, G. E. Scuseria, M. A. Robb, J. R. Cheeseman, G. Scalmani, V. Barone, B. Mennucci, G. A. Petersson, H. Nakatsuji, M. Caricato, X. Li, H. P. Hratchian, A. F. Izmaylov, J. Bloino, G. Zheng, J. L. Sonnenberg, M. Hada, M. Ehara, K. Toyota, R. Fukuda, J. Hasegawa, M. Ishida, T. Nakajima, Y. Honda, O. Kitao, H. Nakai, T. Vreven, J. A. Montgomery, Jr., J. E. Peralta, F. Ogliaro, M. Bearpark, J. J. Heyd, E. Brothers, K. N. Kudin, V. N. Staroverov, T. Keith, R. Kobayashi, J. Normand, K. Raghavachari, A. Rendell, J. C. Burant, S. S. Iyengar, J. Tomasi, M. Cossi, N. Rega, J. M. Millam, M. Klene, J. E. Knox, J. B. Cross, V. Bakken, C. Adamo, J. Jaramillo, R. Gomperts, R. E. Stratmann, O. Yazyev, A. J. Austin, R. Cammi, C. Pomelli, J. W. Ochterski, R. L. Martin, K. Morokuma, V. G. Zakrzewski, G. A. Voth, P. Salvador, J. J. Dannenberg, S. Dapprich, A. D. Daniels, O. Farkas, J. B. Foresman, J. V. Ortiz, J. Cioslowski, and D. J. Fox, *Gaussian 09, Revision E.01*, Gaussian, Inc., Wallingford CT, **2013.**

[S10] a) *ORCA – An ab initio, DFT, and semi-empirical SCF-MO package*, version 4.1.1, Max Planck Institute für Kohlenforschung, Muelheim/Ruhr, Germany; b) F. Neese, *Wiley Interdiscip. Rev.: Comput. Mol. Sci.* **2012**, *2*, 73.

[S11] a) J. P. Perdew, M. Ernzerhof, K. Burke, *J. Chem. Phys.* **1996**, *105*, 9982; b) C. Adamo, V. Barone, *J. Chem. Phys.* **1999**, *110*, 6158.

[S12] T. Yanai, D. P. Tew, N. C. Handy, *Chem. Phys. Lett.* **2004**, *393*, 51.

[S13] a) J. M. L. Martin, A. Sundermann, *J. Chem. Phys.* **2001**, *114*, 3408; b) A. Bergner, M. Dolg, W. Küchle, H. Stoll, H. Preuß, *Mol. Phys.* **1993**, *80*, 1431; c) D. Andrae, U. Huermann, M. Dolg, H. Stoll, H. Preu, *Theor. Chim. Acta* **1990**, *77*, 123.

[S14] F. Weigend, R. Ahlrichs, *Phys. Chem. Chem. Phys.* **2005**, *7*, 3297.

[S15] a) S. Grimme, J. Antony, S. Ehrlich, H. Krieg, *J. Chem. Phys.* **2010**, *132*, 154104; b) S. Grimme, S. Ehrlich, L. Goerigk, *J. Comput. Chem.* **2011**, *32*, 1456.
